# Ten decadal advances in fungal biology leading towards human well-being

**DOI:** 10.1007/s13225-022-00510-3

**Published:** 2022-09-15

**Authors:** Ausana Mapook, Kevin D. Hyde, Khadija Hassan, Blondelle Matio Kemkuignou, Adéla Čmoková, Frank Surup, Eric Kuhnert, Pathompong Paomephan, Tian Cheng, Sybren de Hoog, Yinggai Song, Ruvishika S. Jayawardena, Abdullah M. S. Al-Hatmi, Tokameh Mahmoudi, Nadia Ponts, Lena Studt-Reinhold, Florence Richard-Forget, K. W. Thilini Chethana, Dulanjalee L. Harishchandra, Peter E. Mortimer, Huili Li, Saisamorm Lumyong, Worawoot Aiduang, Jaturong Kumla, Nakarin Suwannarach, Chitrabhanu S. Bhunjun, Feng-Ming Yu, Qi Zhao, Doug Schaefer, Marc Stadler

**Affiliations:** 1 Center of Excellence in Fungal Research, Mae Fah Luang University, Chiang Rai, 57100 Thailand; 2 School of Science, Mae Fah Luang University, Chiang Rai, 57100 Thailand; 3 Department Microbial Drugs, Helmholtz Centre for Infection Research (HZI), and German Centre for Infection Research (DZIF), Partner Site Hannover-Braunschweig, Inhoffenstrasse 7, 38124 Brunswick, Germany; 4 Institute of Microbiology, Technische Universität Braunschweig, Spielmannstraße 7, 38106 Brunswick, Germany; 5 Laboratory of Fungal Genetics and Metabolism, Institute of Microbiology, Czech Academy of Sciences, Prague, Czech Republic; 6 Centre of Biomolecular Drug Research (BMWZ), Institute for Organic Chemistry, Leibniz University Hannover, Schneiderberg 38, 30167 Hannover, Germany; 7 Department of Biotechnology, Faculty of Science, Mahidol University, 272 Rama VI Road, Ratchathewi, Bangkok, 10400 Thailand; 8 Center of Expertise in Mycology, Radboud University Medical Center / Canisius Wilhelmina Hospital, Nijmegen, The Netherlands; 9 Key Laboratory of Environmental Pollution Monitoring and Disease Control, Guizhou Medical University, Guiyang, China; 10 Microbiology, Parasitology and Pathology Graduate Program, Federal University of Paraná, Curitiba, Brazil; 11 Department of Dermatology, Peking University First Hospital, Peking University, Beijing, China; 12 Natural and Medical Sciences Research Center, University of Nizwa, Nizwa, Oman; 13 Department of Biochemistry, Erasmus University Medical Center, Rotterdam, The Netherlands; 14 INRAE, UR1264 Mycology and Food Safety (MycSA), 33882 Villenave d’Ornon, France; 15 Department of Applied Genetics and Cell Biology, Institute of Microbial Genetics, University of Natural Resources and Life Sciences, Vienna (BOKU), Tulln an der Donau, Austria; 16 Beijing Key Laboratory of Environment Friendly Management on Fruit Diseases and Pests in North China, Institute of Plant Protection, Beijing Academy of Agriculture and Forestry Sciences, Beijing, 100097 China; 17 Key Laboratory for Plant Diversity and Biogeography of East Asia, Kunming Institute of Botany, Chinese Academy of Sciences, Kunming, 650201 Yunnan China; 18 Centre for Mountain Futures (CMF), Kunming Institute of Botany, Chinese Academy of Science, Kunming, 650201 Yunnan China; 19 Research Center of Microbial Diversity and Sustainable Utilization, Chiang Mai University, Chiang Mai, 50200 Thailand; 20 Department of Biology, Faculty of Science, Chiang Mai University, Chiang Mai, 50200 Thailand; 21 Academy of Science, The Royal Society of Thailand, Bangkok, 10300 Thailand; 22 Yunnan Key Laboratory of Fungal Diversity and Green Development, Key Laboratory for Plant Diversity and Biogeography of East Asia, Kunming Institute of Botany, Chinese Academy of Sciences, Kunming, 650201 Yunnan China; 23 Innovative Institute of Plant Health, Zhongkai University of Agriculture and Engineering, Haizhu District, Guangzhou, 510225 China

**Keywords:** Biomaterial, CRISPR, Drug development, Morel cultivation, Mushroom cultivation, Mycotoxin biosynthesis, Plastic degradation

## Abstract

Fungi are an understudied resource possessing huge potential for developing products that can greatly improve human well-being. In the current paper, we highlight some important discoveries and developments in applied mycology and interdisciplinary Life Science research. These examples concern recently introduced drugs for the treatment of infections and neurological diseases; application of –OMICS techniques and genetic tools in medical mycology and the regulation of mycotoxin production; as well as some highlights of mushroom cultivaton in Asia. Examples for new diagnostic tools in medical mycology and the exploitation of new candidates for therapeutic drugs, are also given. In addition, two entries illustrating the latest developments in the use of fungi for biodegradation and fungal biomaterial production are provided. Some other areas where there have been and/or will be significant developments are also included. It is our hope that this paper will help realise the importance of fungi as a potential industrial resource and see the next two decades bring forward many new fungal and fungus-derived products.

- **Table of contents**
- **Introduction** F
  1. **Fingolimod, a drug derived by mimetic synthesis from a fungal metabolite as template as a promising immunosuppressive drug for treatment of neurodegenerative diseases** (contributed by Khadija Hassan, Blondelle Matio, Marc Stadler**)**
  2. **From enfumafungin to ibrexafungerp—Development of the first pharmaceutical drug from a fungal endophyte for use in humans** (contributed by Adéla Čmoková, Frank Surup, Marc Stadler, Eric Kuhnert).
  3. **The pleuromutilins, the latest antibacterial drug class that made it to the market, can now be produced by a sustainable biotechnological production process using a heterologous host!** (contributed by Pathompong Paomephan, Tian Cheng, Marc Stadler)
  4. **A newly discovered immune disorder explaining severe mycoses** (contributed by Sybren de Hoog, Yinggai Song)
  5. **Advances in the molecular regulation of the biosynthesis of mycotoxins in *Fusarium*: focus on chromatin structure** (contributed by Nadia Ponts, Lena Studt, Florence Richard-Forget)
  6. **Successful application of CRISPR-Cas9 in medical mycology** (contributed by K.W. Thilini Chethana, Dulanjalee Harishchandra)
  7. **Bioeconomy of mushrooms** (contributed by Peter E. Mortimer, Huili Li)
  8. **Mycelium-based technology** (contributed by Saisamorm Lumyong, Worawoot Aiduang, Jaturong Kumla, Nakarin Suwannarach, Chitrabhanu S. Bhunjun)
  9. **Growing morels in China** (contributed by Feng-Ming Yu, Qi Zhao)
  10. **Fungal genera degrading synthetic plastic polymers** (contributed by Doug Schaefer)
- **Discussion**
- **Acknowledgements**
- **References**

## Introduction

Fungi have been important resources for humankind, starting from the stages of early civilization. Even the most ancient human beings, who were gatherers and hunters, have probably already picked mushrooms and learned the hard way to discriminate between the good and bad choices among their daily diet, which consisted of a mixture of animals, plants, and mushrooms (Beyer 2003; Hyde et al. 2019; Svanberg and Lindh 2019). The earliest human civilizations were founded because the people deliberately cultivated certain types of plants or bred certain types of animals and could thus create a sustainable source of food to support the foundation of larger cities (Ackerman et al. 2014; Raimi et al. 2021). Microscopic fungi, such as certain yeasts and “moulds” have also been used for millennia for production of food and beverages, based on empirical knowledge, even though the early civilisations did not have any scientific background about fermentation processes that result in the production of, e.g., bread, beer, and wine (Hyde et al. 2019). Likewise, certain mushrooms were used traditionally as remedies to treat and cure various kinds of diseases (De Silva et al. 2012). This becomes evident in particular from the ancient Asian pharmacopoeias (Leong et al. 2020; Xu et al. 2021), where ca. one third of the listed ingredients that accound for the “herbal” medicines is actually represented by fungal sources (Yuan et al. 2016; Hyde et al. 2019; Howes et al. 2020; Newman and Cragg 2020).

However, the true beneficial potential of fungi has only come about in the past century, due to the development of sophisticated biotechnological methodology that allows for sustainable production of various products that are highly beneficial to mankind (Hyde et al. 2019). Starting from the discovery of penicillins, large scale fermentation processes were developed for many drug candidates that can now be produced at the kilogram scale, thus marking the starting point of the Golden era of antibiotics (Mohr 2016). Examples such as statins and cyclosporin illustrate that fungal metabolites can also be used efficiently to treat other diseases or make it possible to perform complicated surgeries such as organ transplants (Hyde et al. 2019; Devaux et al. 2021). Biotechnological production processes involving fungal work horses have also been established for the production of enzymes, flavour components, pigments and various commodity chemicals. In recent years, fungi have also been increasinly employed in biodegradation and bioeconomy, e.g., to treat organic waste and gain energy (Filiatrault-Chastel et al. 2021). Last but not least, the importance of fungi as food sources has increased dramatically, and especially in China, the mushroom breeding and production industries (Meyer et al. 2020a, b; Alam et al. 2021; Barzee et al. 2021; Zhang et al. 2021) has made tremendous progress regarding the production of various medicinal and edible species at multi-ton scales (Hyde et al. 2019; Wu et al. 2019).

Many of these accomplishments were facilitated by the availability of various powerful screening systems for detection of enzymatic activities and biological effects at high throughput that are now available in the White Biotechnology, Agro and Pharma Industries. However, in particular the newly arising –OMICS technologies and the corresponding progress in molecular genetics and biochemistry have even facilitated the development of new products and processes in all the aforementioned areas and offer new diagnostic tools (Wibberg et al. 2021). As shown in the latter study and the follow-up work by Kuhnert et al. (2021), third generation genome sequencing technologies provide high quality sequence data, and important loci such as biosynthetic gene clusters can easily be made out in the almost complete genomes and exploited subsequently by methods of synthetic biotechnology. As of recently, innovative methods such as synthetic biology or gene editing that are based on genome data have become routine in various fields of unnecessary the Life Sciences (Khalil 2020; Li et al. 2020; Martin et al. 2020) and are essential to the proposed circular bioeconomies (Pan 2017; Meyer et al. 2020a, b; Lange et al. 2021; Venkatesh 2022).

In the current paper, we summarize what we consider to be ten important decadal advances in fungal biology that will improve human well-being and, in some cases, alleviate climate change and reduce polluting the planet.

## Fingolimod, a drug derived by mimetic synthesis from a fungal metabolite as template as a promising immunosuppressive drug for treatment of neurodegenerative diseases

Autoimmune disorders of the central nervous system (CNS) like chronic multiple sclerosis (MS) have prompted an intensive search for new immunomodulatory drugs against neurological disorders of the central nervous system in last 20 years. An estimation of up to 2.5 million people in the world are said to have multiple sclerosis, making it the leading cause of neurological disorders (Rosati 2001).

Current treatment strategies in multiple sclerosis involve management of symptoms and use of disease-modifying drugs like; intramuscular (IM) interferon beta-1a (IFNβ-1a), subcutaneous (SC) IFNβ-1a, SC IFNβ-1b, and glatiramer acetate (GA), all of which must be injected (O'Rourke and Hutchinson 2005; Haas and Firzlaff 2005). However, severe injection-site reaction incidence in addition to flu-like symptoms, depression, and fatigue has led to the discontinuation of the therapy (Stewart and Tran 2012). The introduction of oral therapies has been a huge step forward in the treatment of relapsing–remitting multiple sclerosis, firstly due to ease of their administration in addition to parameters such as clinical efficacy, ability to reduce lesions, safety, and tolerability.

### Immune-mediated disorders affecting the central nervous system

The body’s immune system plays a major role combating different diseases and infections by recognizing foreign disease-causing pathogens and tumors and eliminating them. In some cases, the immune system may have abnormal responses and start attacking the body in what is known as an immune-mediated disease (García et al. 2020). Immune-mediated diseases are those whose cause is thought to be modulated by an inappropriate immune response (David et al. 2018). In such an abnormal response, the immune system attacks and destroys healthy and normal cells such as the red blood cells or platelets. In the case of immune-mediated disorders affecting the central nervous system, the immune system attacks a particular location in the central nervous system.

This attack by the immune system alters the cellular homeostasis and causes injury to the affected organs since there is an excessive inflammatory reaction in response to the attack (Groves et al. 2013). The response is the uncontrolled production of antigens as an inflammatory response, with the cytokines and the CD4+T lymphocytes being the most common response, although other lymphocyte types such as TH1 or TH2 may also be produced in response to the attack (García et al. 2020; Ghasemi et al. 2017). The occurrence of immune medicated diseases often has a complex etiology with genetic factors being the major component, and triggered by environmental, genetic, and infectious agents (David et al. 2018; García et al. 2020).

Immune mediated disorders are an issue of public health significance since patients with one autoimmune disorder tend to develop additional conditions, including both autoimmune disorders and other comorbid conditions (Reale et al. 2018). There is an increased susceptibility of patients with one autoimmune disease developing an additional syndrome, which increases the burden of treatment and management of the disease (Brinkmann et al. 2010). Conditions such as multiple sclerosis are also associated with a higher burden of cardiovascular disease, as the body’s inflammatory response serves to induce atherosclerosis (Reale et al. 2018). In other cases, the immune system reaction triggers the development of myocarditis due to the damage of cells both infected and uninfected by viruses, which can be fatal to the patient.

Apart from cardiovascular disorders, immune mediated diseases can also lead to neuroinflammation, causing degenerative disorders such as Parkinson’s and Alzheimer’s disease, multiple sclerosis, and stroke, among others. These diseases lead to progressive damage and degeneration of neurons due to host immune response. According to Sanford (2014), immune mediated diseases such as multiple sclerosis are associated with clinical and economic burdens due to the nature of the disease. Conditions such as multiple sclerosis are progressive and occur over many years, often over the individuals’ lifespan and require support from the family, caregivers, and the healthcare system, which often comes at a high cost (Owens 2016).

As mentioned, conditions such as multiple sclerosis are immune-mediated disease that occur due to the body’s immune system attacking the central nervous system and destroys the myelin, oligodendrocytes and nerve fibers (Ghasemi et al. 2017). The damage due to an abnormal immune response result in scarring on different areas, thus the name multiple sclerosis. This damage affects the ability of the central nervous system to conduct signals from the brain to different parts of the body. The result is the different physical, cognitive, and neurological symptoms that occur due to poor transmission of impulses within the central nervous system, which vary among patients depending on the type and severity of the condition (Ghasemi et al. 2017).

### New treatments and diagnostic methods under development

Testing and diagnosis for multiple sclerosis typically relies on ruling out other conditions that may present with similar signs and symptoms. However, the use of Magnetization Transfer imaging (MTR) based markers has been found to be more useful in detecting, monitoring, and understanding the progression of multiple sclerosis under treatment (Petracca et al. 2018). The use of advanced imaging techniques is helpful in detecting changes due to the enhanced sensitivity of the tests. However, being novel techniques, there are still gaps in understanding the sensitivity of the tests longitudinally. There is need to ensure the standardization of tests, processing, and the development of images in high resolution for this to be a viable testing and diagnostic tool (Petracca et al. 2018).

The treatment of autoimmune diseases has traditionally focused on the use of immuno-suppressive therapy that lowers the patients’ immune response. However, this approach requires long-term use of progressively increasing dosages to maintain disease control, often exposing the patients to opportunistic infections that may be fatal. In addition, the use of immunosuppressants is also associated with toxicity and adverse side effects that affect the quality of life for the patients (Rosenblum et al. 2014). This indicates the need for research and development into new treatment and diagnostic modalities.

One such novel approach to immune-medicated diseases is the use of costimulatory blockade. T-cells play a major role in the immune system, being involved in the killing of infected host cells, activating other immune cells and regulating the body’s immune responses. In the absence of pathogens and other disease-causing factors, the T-cells become fully activated, resulting in an autoimmune response. Blocking the pathways that result in this activation is the costimulatory blockade, where the costimulatory signals responsible for activating the T-cells are inhibited, reducing the effect of the autoimmune response (Rosenblum et al. 2014). The strategy proved to be useful in preventing disease, such as type I diabetes or rheumatoid arthritis. In contrast, the effectiveness of the approach on multiple sclerosis has been rather low, which may be attributed to the fact that the approach has less effect on the T-cells that were previously activated, with the costimulatory blockade being unable to suppress the cells (Rosenblum et al. 2014).

Apart from synthetic medication, natural products also play a major role in the treatment and management of autoimmune diseases such as multiple sclerosis. As mentioned, the use of immunosuppressive therapy is associated with the increased need of medication at higher dosages, which exposes the patient to opportunistic infections (Rosenblum et al. 2014). Natural products play a critical role in the discovery and development of drugs, due to their structural ability to regulate the body’s defense function and also competition with disease causing pathogens, thus their effectiveness in the treatment of cancerous cells and other infectious disease (Atanasov et al. 2021; Newman and Cragg 2016).

The use of natural products in the management of multiple sclerosis has made great strides in the last decade, with several biological immunomodulators derived and developed, and shown to be effective in suppressing the immune response and in slowing down the progression of the disease (Gharagozloo et al. 2018). Many of these compounds are secondary metabolites of fungi and bacteria, which are characterized by having complex, unique structures and relatively high molecular weights. These properties make the secondary metabolites highly suitable as candidates for pharmacological drug development (Atanasov et al. 2021).

Despite the potential benefits of natural products in the treatment and management of multiple sclerosis and other disorders, there are challenges faced in successfully developing and discovering drugs from these chemical entities. Those challenges range from the identification of the biologically active compounds in the extracts, to legal barriers in patenting the bioactive compounds (Atanasov et al. 2021). As mentioned, multiple sclerosis, is a progressive degenerative disease, and the goals of treatment are to improve the patients’ quality of life (Gil-González et al. 2020), thus the need and emphasis for products that will have an anti-inflammatory response, enhance immune regulation, and repair the damage to the myelin sheath in the central nervous system (Ghasemi et al. 2017).

### Immunosuppressive natural products

**Fig. 1:**
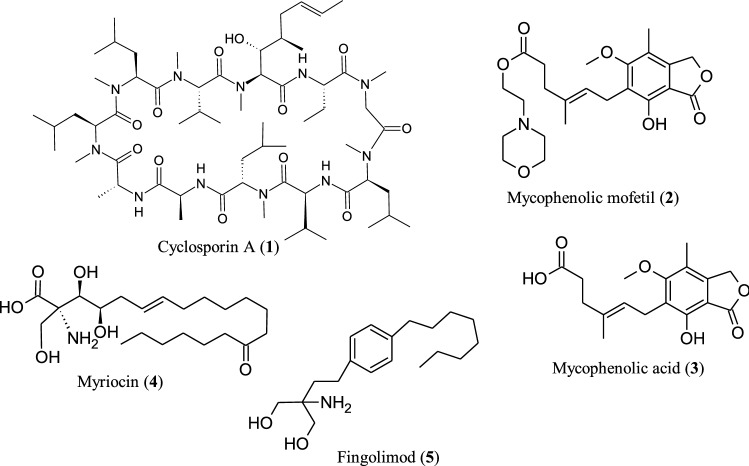
Fungi-derived natural immunosuppressants

Natural products historically have been an incredible source of new therapeutic agents both in their natural form and as template for semisynthetic and synthetic modification (Atanasov et al. 2021). They are representative of a very wide divergent structures and became a dynamic source of drug discovery for the treatment of various ailments including autoimmune diseases (Pham et al. 2019; Harvey et al. 2015). Autoimmune diseases (ADs) are pathological conditions, which occur due to loss of immunological tolerance towards self-antigens leading to damage and dysfunction of specific or multiple organs and tissues (Singh et al. 2016). Medication, especially the use of immunosuppressive drugs, is the primary therapy for treating autoimmune diseases (Guo et al. 2018). Immunosuppressant agents are used to prevent the immune system from acting against transplanted tissues and/or organs such as the heart, liver and kidneys (Holt 2017). Therefore, the development of clinical immunosuppressive agents for autoimmune diseases provided solutions for drug discovery and development. Fungi-derived natural products and their semisynthetic derivatives have made important contributions in providing potent immunosuppressants with unique modes of action (Fig. 1). Some of the important clinically used immunosuppressive agents produced by fungi during the 1980s and the early 2000s are Cyclosporin A (**1**) (Beekman and Barrow 2014) from the ascomycete *Tolypocladium inflatum* (Dreyfuss et al. 1976) and mycophenolic mofetil (**2**) the approved pro-drug for mycophenolic acid (**3**). The latter compound was originally isolated from *Penicillium* spp., including *Penicillium brevicompactum*, *P. stoloniferum* and *P. roqueforti* (Gosio 1896; Beekman and Barrow 2014; Patel et al. 2017). The discovery and development of these compounds supported and validated the screening of fungi in pursuit of lead compounds for the development of new immunosuppressive drugs with novel mode of action, improved efficacy, and reduced side effects.

In this context, the screening of extract from the ascomycete *Isaria sinclairii* by Fujita and colleagues and the evaluation process guided the isolation of a compound with significant immunosuppressant activity (Fujita et al. 1994a). The isolated compound was termed ISP-I and the structure seemed to be identical to that of myriocin (**4**), previously isolated from *Melanocarpus albomyces* in a screening for antifungal agents (Kluepfel et al. 1972). In a mixed lymphocyte reaction (MLR) assay, myriocin was shown to be more potent than cyclosporin A (Fujita et al. 1994a). However, it turned out to be more toxic compared to cyclosporin A (Fujita et al. 1996a), hence, researchers began to study myriocin in order to improve its biological properties (Fujita et al. 1994b, 1996b). A medicinal chemistry program to optimize the activity of myriocin based on mimetic synthesis (Adachi et al. 1995), subsequently led to the identification of a novel compound: Structure activity relationship (SAR) studies guided the discovery of the highly effective immunosuppressive agent fingolimod (**5**), also known as FTY720 or 2-amino-2-[2-(4-octylphenyl) ethyl]propane diol hydrochloride (Adachi et al. 1995; Kiuchi et al. 2000).

In part reflecting its origins in transplantation research, fingolimod has been extensively studied for its effects on immune system. The therapeutic activity of the drug has demonstrated improved efficacy compared to other oral treatment products such as teriflunomide and dimethyl fumarate (Stewart and Tran 2012). In addition, initial in vitro findings indicated that fingolimod has an effect on disability and reduction of brain atrophy. It also retainis central but not effector memory T cells in lymph nodes, which leads to a preferential reduction of multiple sclerosis-pathogenic immune responses and spares large parts of protective immunity (Foster et al. 2007; Miron et al. 2008). Furthermore, its lipophilic nature enables the drug to cross the blood–brain barrier, which may help restore gap-junctional communication of astrocytes with neurons and cells of the blood–brain barrier (Aktas et al. 2011). This phenomenon is associated with neurodegeneration in multiple sclerosis.

Fingolimod was finally approved in 2010 by the USA Food and Drug Administration (FDA) as the first oral drug used for the treatment of Relapsing Remitting Multiple Sclerosis (RRMS) under the trade name Gilenya® (Thomas et al. 2017). The drug is applied as a hard gelatin capsule including 0.56 mg fingolimod hydrochloride (Thomas et al. 2017). Since its discovery, several researchers have investigated in the mechanism of action of fingolimod in multiple sclerosis as well as its potential application as therapy for the treatment of other autoimmune-related diseases (Huwiler and Zangemeister-Wittke 2018).

Curiously, the taxonomy of the producer organisms of this important drug template has not been revised to date, and there is no other report on the discovery of myriocin from another member of the genus *Isaria* and allies, even though these fungi have been studied rather thoroughly during the past decades (Isaka et al. 2005; Zhang et al. 2020a, b, c, d).

**Fig. 2:**
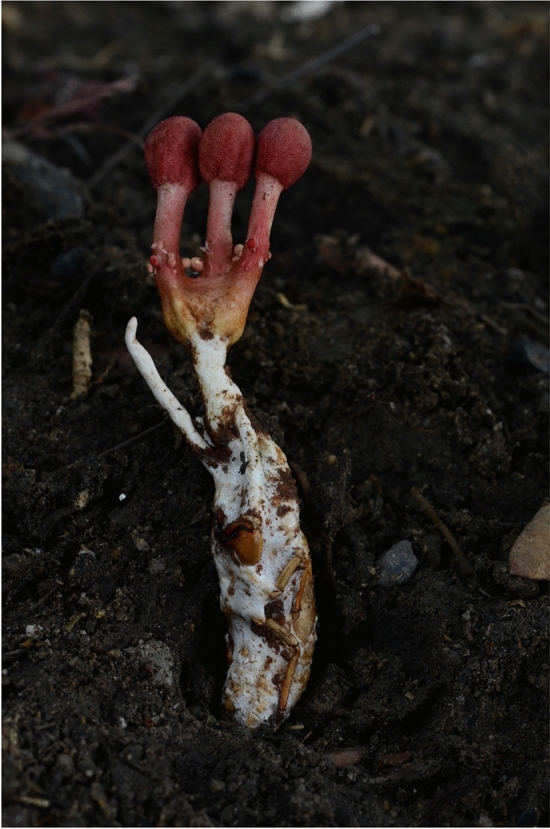
Stromata of *Ophiocordyceps khonkaenensis.* Kindly provided by Artit Khonsanit

We have researched the taxonomic history of the producer organism (and Hypocreales from cicada in general) and interestingly, we found that the taxonomy of the producer strain reported by Fujita et al. (1994a) is incorrect! The name *Isaria sinclairii* actually goes back to a species that was described by Berkeley (1855) based on a specimen from New Zealand, as *Cordyceps sinclarii* Berk. The American mycologist Lloyd (1923) later transferred the species to the genus *Isaria*, but did not study the type specimen that is housed in the Kew Botanical Gardens, UK. His observations were based on another specimen that was sent to him from New Zealand, and the transfer of a *Cordyceps* species to *Isaria* without any type studies would be regarded rather questionable today. However, the taxonomy of these insect associated fungi has changed drastically over the past decades (Sung et al. 2007; Kepler et al. 2017; Xiao et al. 2019, 2022). For instance, most of the insect-associated hypocealean species are now distributed over three different families, Cordycipitaceae, Clavicipitaceae and Ophiocordycipitaceae (Xiao et al. 2019, 2022; Zhang et al. 2020a, b, c, d; Wijayawardene et al. 2022). Various generic rearrangements have been proposed on the basis of large phylogenetic studies using multi-DNA locus genealogies and the One-Fungus-One Name Concept. The definition of genera like *Cordyceps* and *Isaria* has also changed accordingly, and many species that were formerly accommodated in “*Cordyceps *sensu lato” are now actually members of *Ophiocordyceps* or other genera of Ophiocordycipitaceae. This even includes the famous Chinese Caterpillar Fungus, which is widely used in Asian Traditional Medicine and now bears the name *Ophiocordyceps sinensis* (Zhang et al. 2013). Likewise, there are species of both *Cordyceps* and *Ophiocordyceps* that are known to be associated with Cicadae insects. The strain that was reported by Fujita et al. (1994a) as the original producer of myriocin is deposited with ATCC (strain number 24400) and sequence data released to GenBank also point toward its being a member of *Ophiocordyceps*. The closest relatives as inferred from a comparison of the sequence data are *Ophiocordyceps sobolifera* (Ban et al. 2015) and *O. khonkaenensis* (Crous et al. 2019), i.e., two species that were described from cicada in Asia (Fig. 2). Phylogenetically, this ATTC strain is far apart from all cicada parasites in *Cordyceps*. Even though the species *Isaria* (*Cordyceps*) *sinclairii* has apparently never been cultured and sequenced, the species that appear morphologically most similar to it, like *Cordyceps jakajanicola* (also reported by Crous et al. 2019) and grow on cicadae in Asia definitely differ from it. Interestingly, Lloyd (1923) had already remarked that the type of *Cordyceps/Isaria sinclarii* was similar to “*Cordyceps sobolifera*”, but this was never taken into account when the genera and families of the invertebrate-associated taxa were rearranged. We conclude that the information on the original producer strain of myriocin needs to be corrected to “*Ophiocordyceps* sp.”, even though the species cannot be narrowed down with certainty because the stromata from which the ATCC culture was obtained are not apparently available for taxonomic revision. Studies are presently ongoing to verify whether the phylogenetically close strains to “*Isaria sinclairii* ATCC 24,400” are able to produce myriocin, so this riddle can be solved.

### Synthetic methods for preparation of fingolimod

The first method for the synthesis of fingolimod (code name FTY720), with the IUPAC name 2-amino-2-[2-(4-octylphenyl) ethyl]propan-1,3-diol hydrochloride, was reported by Adachi et al (1995). From there on, several processes for the preparation of fingolimod free base and/or its hydrochloride have been developed (Chaturvedi et al. 2020). Although several literatures known synthetic strategies of fingolimod have been reported, most of them seem to be tedious and time consuming and therefore not industrially and economically feasible (Chaturvedi et al. 2020). The aim of devising efficient and viable routes amenable to scale-up associated with improved yield and quality to the small molecule drug for the treatment of multiple sclerosis therefore became a priority for organic chemists such as Chaturvedi AK, who provided a process for synthetic preparation of fingolimod hydrochloride with a purity greater that 99.8%. Basically, this process involves 4 or 5 steps to obtain fingolimod free base and fingolimod hydrochloride, respectively, starting from 2-acetamido-2-(4-octanoylphenethyl)propane-1,3-diyl diacetate via a Friedel–Crafts acylation using octanoyl chloride in the presence of a Lewis acid. A remarkable feature of this synthetic route, which was an improvement over prior disclosed methods, is the fact that it does not involve the use of column chromatography in the entire process.

### Mode of action and potential applications of fingolimod

**Fig. 3:**
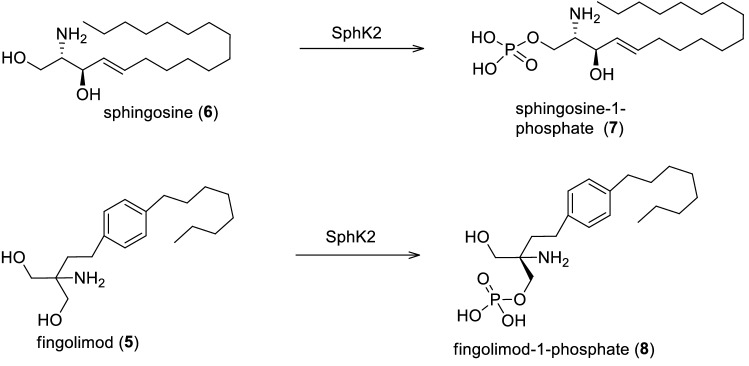
Mechanism of phosphorylation of sphingosine and fingolimod via sphingosine kinase 2 (Strader et al. 2011)

Fingolimod is a sphingosine-1-phosphate receptor modulator that is rapidly metabolized in vivo following phosphorylation by sphingosine Kinase 2 (SphK2) to produce the phosphorylated and active form of fingolimod-phosphate (Brinkmann 2009; Huwiler and Zangemeister-Wittke 2018) (Fig. 3). After phosphorylation, the active moiety fingolimod-P exerts its effects by mimicking sphingosine 1-phosphate (S1P) and binds to four G protein-coupled sphingosine-1-phosphates receptors (GPCRs). Up till now, five S1P receptors termed S1P_1-5_ have been identified and fingolimod-P binds with similar affinity as S1P to S1P_1_, S1P_3_, S1P_5_ and shows much better binding to S1P_4_ than S1P. Unlike S1P fingolimod-P is not a ligand for S1P_2_ (Huwiler and Zangemeister-Wittke 2018). Fingolimod-P binding to S1P receptors inhibits the egress of lymphocytes from lymph nodes thus preventing them from contributing to autoimmune processes including inflammatory injuries characteristic of multiple sclerosis (Brinkmann 2009). A significant reduction in the relapses was observed in patients treated with fingolimod. Moreover, fingolimod was also reported to contribute to neuroprotection in the central nervous system as it can easily cross the blood–brain-barrier (BBB) based on its high lipophilic nature and is thought to exert effects directly on resident central nervous system cells, which also express S1P receptors (Hunter et al. 2016).

Currently, there is a significant interest in the potential benefits of fingolimod on several other autoimmune disorders. Its mechanism of action and potency against those have therefore been extensively investigated in preclinical studies, some of which moved forward to clinical trials. This includes stroke for which fingolimod was tested in phase II clinical study in patients with acute ischemic stroke (Fu et al. 2014; Tian et al. 2018). In addition, fingolimod showed efficacy against Amyotrophic Lateral Sclerosis (ALS) and a clinical phase II study was performed to determine safety and tolerability of fingolimod in patient with ALS; the drug actually demonstrated acceptable acute safety and tolerability (Potenza et al. 2016; Berry et al. 2017). However, despite the potential benefits of fingolimod against the above mentioned autoimmune diseases, RRMS currently remains as the only approved indication for fingolimod. Another indication of interest for fingolimod is directed towards autoimmune disorders associated with neuroinflammatory processes such as Alzheimer’s Disease, Parkinson’s Disease, and cerebral malaria (Huwiler and Zangemeister-Wittke 2018).

## From enfumafungin to ibrexafungerp—Development of the first pharmaceutical drug from a fungal endophyte for use in humans

The current chapter deals with the discovery and development of the first drug for treatment of systemic infections from an endophytic fungus. Contrary to what has been frequently written in the literature and even in some renowned scientific publications, fungal endophytes have never been proven to be capable of sustainable biotechnological production of plant metabolites like taxol. For instance, the taxadiene synthetase, which is a key enzyme of taxol biosynthesis in the plant, could not be detected in the genome of “*Taxomyces andreanae*”, the fungus that was claimed to produce taxol in the study by Stierle et al. (1993), or in any other fungus (cf. Heinig et al. 2013). In fact, no one has even isolated a mg of taxol from a fungal source, while the compound can be produced at kg scale from needles or cell cultures of *Taxus* spp. Heinig et al. (2013) observed that traces of taxol were only detectable by HPLC–MS in primary cultures of yew endophytes they isolated themselves. The compound was not detected anymore after passaging the endophyte culture onto new media. The authors speculated that the traces of taxol may have been carried over from the plant tissue, but it is hard to prove a negative.

In any case, endophytic fungi have their own repertoire of biosynthesis genes that are not much different from those of their saprotrophic counterparts. In some cases, it has even been possible to relate the endophytic producers of developmental drugs or other endophytes that have high biotechnological potential to a certain teleomorphic state, thereby elucidating their life cycles (Bills et al. 2012; Samarakoon et al. 2020; Wittstein et al. 2020). The latter paper actually treats the producers of the cylodepsipeptide PF1022-A, whose derivative emodepsin was so far the only marketed drug from an endophyte, even though it is only used for treatment in veterinary medicine and was not yet approved for use in humans.

Below we will first highlight the problems and challenges associated with fungal infections, then summarize the current treatment options and finally report on the discovery of this first “endophytic drug” for human use and its preclinical and clinical development. We also deal with the taxonomy of the producer organism and the biosynthesis of the beneficial molecule.

### Importance of fungal infections to human health and overview of the limited treatment options available

**Fig. 4:**
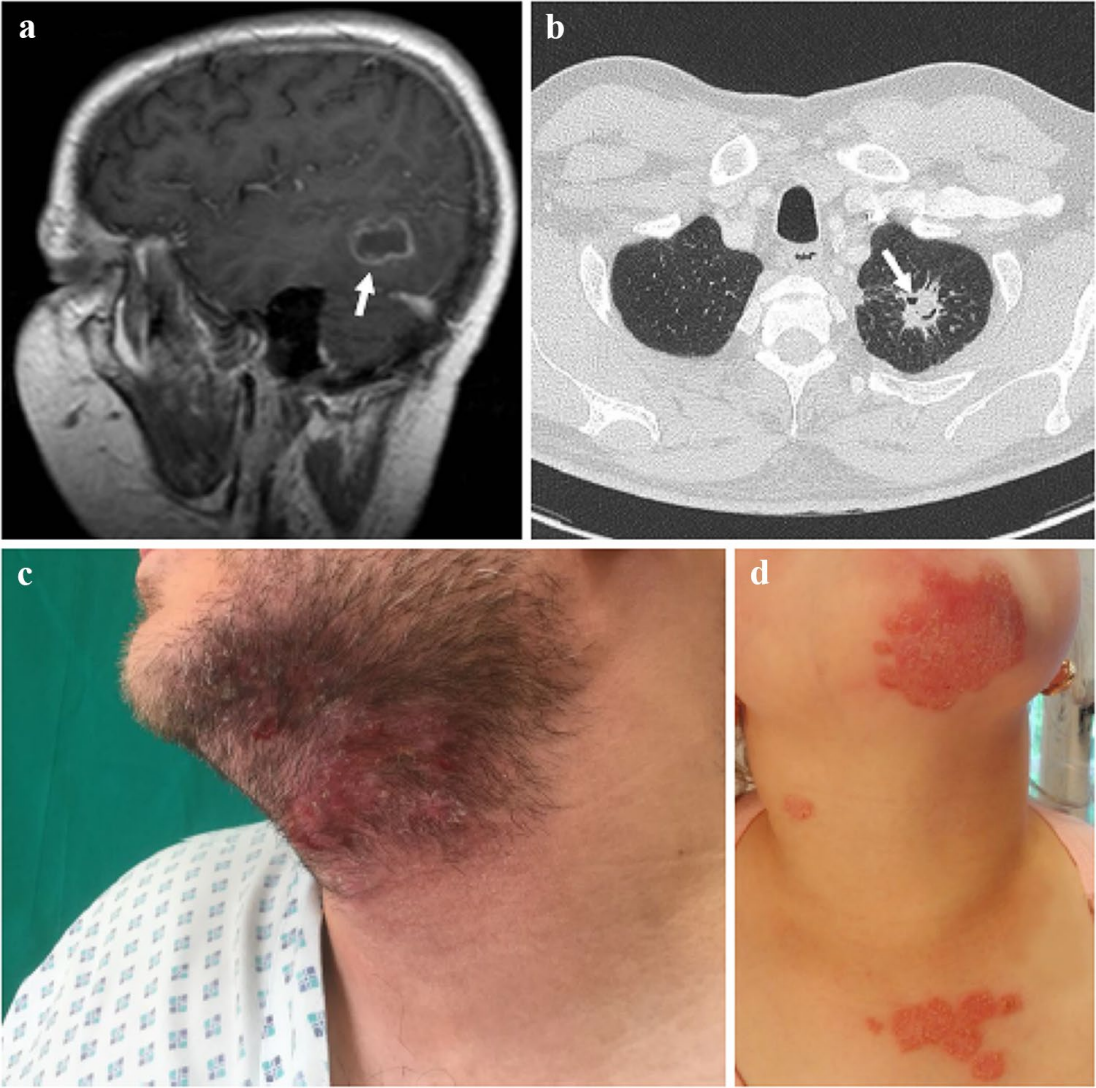
Clinical manifestation of **a** and **b** invasive fungal diseases caused by *Aspergillus* and **c** and **d** zoonotic dermatomycosis due to species from *Trichophyton benhamiae* clade. **a** A magnetic resonance image of the head showing an abscess (indicated with white arrow) in the left temporo-occipital area of 17-year-old boy with chronic granulomatous disease. **b** Chest computed tomography image showing lesion (white arrow) consistent with acute invasive pulmonary aspergillosis in a cancer patient. **c** Dermatophytosis localized on the bearded areas. **d** Infection localized on the neckline and face of 9-year-old girl contracted from guinea pig

Fungal pathogens significantly affect lives of more than 80% of the present human population (Bongomin et al. 2017). However, despite causing 1.5 million deaths worldwide annually, pathogenic fungi are rather neglected and understudied infectious agents with underfunded research when compared to the other pathogens (Almeida et al. 2019; Benedict et al. 2019). One of the main reasons is that it has long been believed that fungal pathogens have only a relatively low health impact in healthy people. Healthy individuals are endangered mostly by few primary fungal pathogens while the majority of fungal pathogens are opportunistic and affect primarily immunocompromised patients (Fig. 4). In addition, infections caused by the most dangerous (i.e. biosafety level 3 organisms) primary fungal pathogens are rare because their distribution is geographically restricted mainly to a few endemic areas (Benedict et al. 2015). Other primary pathogens, species of dermatophytes (Arthrodermataceae), causing skin infections (dermatophytoses), tend to have broader areas of distribution and are among the most common global human pathogens (Havlickova et al. 2008). For example, a recent outbreak of children skin infections caused by strains of the guinea-pig associated pathogenic fungus *Trichophyton benhamiae* shows that events such as the rapid spread of fungi in naïve host population driven by novel adaptation to host immune system may occasionally occur, and then represent a potential risk for the population (Čmoková et al. 2020). Rapid expansion of fungal pathogen in naïve hosts can be cause a significant reduction of populations of many animals (e.g. bats, snakes and amphibians) (Zukal et al. 2016; Rebollar et al. 2016; Allender et al. 2015).

In contrast to dermatophytoses, which mostly cause non-life-threatening infections and usually do not arouse considerable public interest, opportunistic pathogens such as *Aspergillus* spp. and yeasts of the genera *Candida*, *Cryptococcus* and *Pneumocystis* are responsible for most fatal infections, dramatically reducing the risk of survival of patients in hospitals (d’Enfert 2009) (Fig. 4). The risk of fungal infection is particularly high in those patients with low immunity response and also in patients whose treatment involves the use of artificial surfaces, such as plastic intravenous lines and cannulas (Poowanawittayakom et al. 2018). A current striking example of such “risk groups” are COVID-19-infected patients that are undergoing bronchoscopy (Bartoletti et al. 2020; Koehler et al. 2021). Some opportunistic pathogens may even overcome the immune system of healthy individuals and cause chronic or fatal infections. *Cladophialophora bantiana*, *Talaromyces marneffei* and *Candida auris* were chosen as examples of most feared opportunistic fungal pathogens responsible for increasing number of fatal infections in healthy individuals, particularly in a case of *C*. *auris* because of limited treatment options due to multidrug resistance (Hyde et al. 2018a). However, *C*. *auris* represents only one of many pathogens facing a threat of treatment failure due to more and more limited treatment options. Only three classes of antifungal drugs are currently available to treat invasive mycoses and one additional class is registered for treatment of non-systemic fungal infections (Walsh et al. 2008). Moreover, many of these pathogens have become resistant over time, including azole resistant *Aspergillus*, terbinafine resistant dermatophytes, fluconazole resistant *Coccidioides*, multidrug resistant strains of *Candida, Lomentospora*, *Microascus*, *Scedosporium*, and *Scopulariopsis* (Ebert et al. 2020; Du et al. 2020; Al-Hatmi et al. 2019; Mello et al. 2019; Pérez-Cantero and Guarro 2020). Thus, if the pathogens become resistant to one class, the therapeutic options are significantly reduced. This is challenging especially in patients undergoing invasive aspergillosis where already high mortalities (29–50%) (Nivoix et al. 2008; Webb et al. 2018) further increase up to 88% in case of infection by antimycotics resistant strains (van der Linden et al. 2011). The situation regarding the current state of the art in terms of the development of new antimycotics is unfortunately not ideal, also considering that the populations in the highly industrialized “rich” countries of the world is getting older on average. The pipelines for novel antimycotics are even more empty than the ones for new antibacterial antibiotics, which have fortunately been filled again to some extent, owing to massive funding for basic and translational research after over twenty years of negligence (Gintjee et al. 2020).

New antifungal agents are as badly needed as new antibacterials because the lack of remedies against fungal pathogens also affects agriculture, where bacteria do by far not cause as much damage. Instead of prevention, decisions and behaviour of the human society has inadvertently supported the emergence of new resistant strains in both of these areas. For instance, the azole antimycotics are also widely used in agriculture, although the majority of resistant strains may have originated in fields treated by azole antifungals (Berger et al. 2017; Cao et al. 2021b). Recently, some countries have replaced azole antifungals in agriculture by alternatives, but nevertheless, azole resistance has increased rapidly all over the world (Van der Linden et al. 2015; Howard et al. 2009). Importantly, there are not so many alternatives on the market neither in human medicine, nor in agriculture, where azoles represent the key fungicides, especially, when also other fungicides lost approval (succinate dehydrogenase inhibitors) or face resistance (e.g., quinone outside and sterol demethylation inhibitors) of important plant pathogens (Birr et al. 2021; Lammari et al. 2020; Pan et al. 2020) and fungicides in mixtures with azoles seems to be the only option to avoid famines. Such a situation is mainly because of lack of innovations caused by insufficient financial incentives due to undervaluation of the critical situation.

### Validated targets for antifungal therapy

**Fig. 5:**
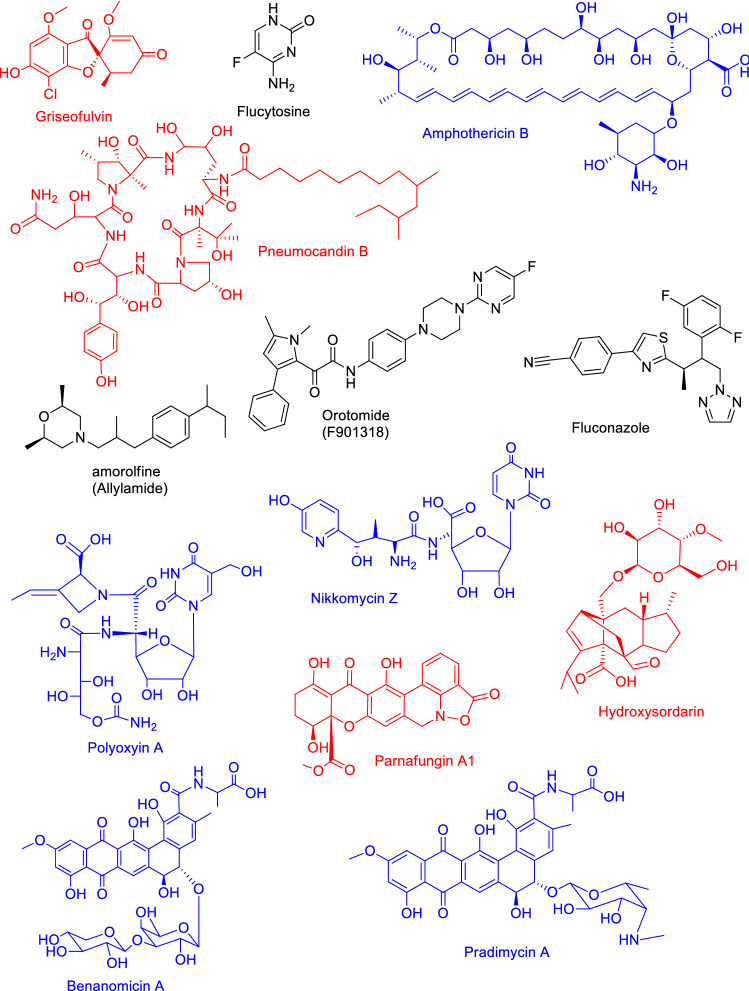
Antimyotic agents approved or under development for treatment of fungal infections. Fungal metabolites are printed in red and metabolites from Actinobacteria are printed in blue; the others are of synthetic origin

When compared to the other pathogens (in particular the prokaryotic bacteria), development of fungal drugs requires more effort also because of close relatedness between fungi and animals. Hence, the number of possible targets of clinically available drugs for invasive fungal infections is currently limited to merely three major classes (Fig. 5).

A) **Echinocandins**, which affect the biosynthesis of 1,3-β-d-glucan of the fungal cell wall,

B) **Azoles**, which inhibit the biosynthesis of ergosterol.

C) **Polyenes**, which bind this sterol, resulting in formation of pores in the fungal membrane.

Another class of antimycotics, represented by the antimetabolite **flucytosine**, has limited treatment options. After bioconversion in the human body, this compound interacts with DNA and RNA biosynthesis and thus disturbs the synthesis of several essential proteins. Two more options are available for dermatophytosis and other non-systemic fungal infections, i.e. the rarely used “old dog” antimycotic **griseofulvin**, which binds to microtubule and inferring its function (Rathinasamy et al. 2010), and **allylamines** which inhibit squalene epoxidase enzyme, resulting in low levels of available ergosterol and accumulation of squalene in the cells (Ryder and Frank 1992).

However, only a few antifungal compound classes are undergoing clinical trials or are in preclinical development. An example are the **orotomides**, which inhibit the fungal enzyme dihydroorotate dehydrogenase (Hope et al. 2017; Wiederhold 2020), **sordarins** (Domínguez et al. 1998) with unique mode of action resulting in protein synthesis inhibition, and **parnafungins** with inhibition of poly(A) polymerase activity (Parish et al. 2008; Jiang et al. 2008). The fungal cell wall in particularly represents an attractive antifungal drug target because of missing homological structure in human cells and thus its low toxicity in humans (Butts and Krysan 2012). Besides echinocandines, some antifungals target cell wall structures synthesis, such are inhibitors of chitin synthesis (e.g. **nikkomycins, polyoxins**), mannoprotein-binding antifungal agents (e.g. **pradimicins** and **benanomicins**), and finally, another inhibitor of β (1, 3)-D-glucan synthesis but with a unique mechanism of action as compared to echinocandins (**enfumafungins**) (Curto et al. 2021). The molecular interaction site of enfumafungins was proposed to be located at the same target site as the echinocandins, the fungus specific (1,3)-ß-glucan synthase component FKS1. However, enfumafungins exhibit only limited cross-resistance to echinocandin-resistant isolates, suggesting a difference in binding site (Jiménez-Ortigosa et al. 2014, 2017).

In view of the above situation, the following paragraphs highlight a very fortunate positive new development, namely the history of the discovery of the first endophyte-derived antimycotic drug launched on the market.

### Discovery and description of *Hormonema carpetanum* and enfumafungin

*Hormonema carpetanum* is a member of the black yeast-like fungi related to *Aureobasidium pullulans* in the order Dothideales (Bills et al. 2004). The characteristics of the genus are the melanized, but otherwise undifferentiated hyphae that produce slimy, yeast-like blastoconidia. The latter are formed basipetally from one or few loci directly on the vegetative hyphae.

Traditionally, the genus has been discriminated from the morphologically similar *Aureobasidium* and other genera of dematiaceous Dothideales by having percurrent conidiogenous loci (rather than the synchronous conidiogenesis observed in *Aureobasidium*; cf. de Hoog and Hermanides-Nijhof 1977; de Hoog and Yurlova 1994). In addition, some species have been shown to be able to form a pycnidial synanamorph.

*Hormonema* was introduced by Lagerberg (1927) and is typified by *H. dematioides* Lagerb. & Melin. The genus presently comprises eight species. It is included in the Dothideales (Class Dothideomycetes) and has been placed in the family Dothioraceae (Wijayawardene et al. 2020, 2022). These taxa belong to the “black yeasts”, which are considered to be among the most complicated groups of Ascomycota in terms of taxonomy. For instance, the taxonomy of *Hormonema* is complicated because the type species is regarded as the asexual state (and therefore, following the 1F1N rule, would constitute a later synonym) of *Sydowia polyspora* (Bref. & Tavel) E. Müll, which has the basionym *Dothidea polyspora* Bref. & Tavel and is therefore the much older name. On the other hand, the type species of *Sydowia* is *S. gregaria* Bres. It remains unclear whether *Sydowia*/*Hormonema dematiodes* is phylogenetically closely related, because *Sydowia gregaria* has never been subjected to molecular phylogenetic studies and like many other names in the Dothideales, needs an epitypification. This could be best accomplished by collecting *S. gregaria* from *Abies* in Germany (i.e., the host plant and country from where the fungus was initially reported) and by designating an epitype (Ariyawansa et al. 2014).

The few available phylogenetic studies (e.g., Bills et al. 2004; Humphries et al. 2017) have thus failed to provide a clear picture on the relationships of *Hormonema* and its allies. Anamorph-teleomorph relationships also remain to be established for most of the species of this group, albeit all teleomorphic taxa so far shown to have a hormonema-like asexual state also have depressed-globose, erumpent, solitary ascomata featuring bitunicate asci, as typically observed in the family and order. The aforementioned pycnidial synanamorphs can also be classified in several morphological genera and no correlations have so far become evident between the different morphs observed in the genus. Therefore*,* both, *Sydowia* and *Hormonema* remain in use *ad interim*. A large scale polythetic study, perhaps even employing chemotaxonomic methods in addition to morphological studies and a multilocus phylogeny, should be the best way to solve the problems with the taxonomy of these fungi (Thambugala et al. 2014; Wijayawardene et al. 2014; Humphries et al. 2017).

*Hormonema carpetanum*, the producer of enfumafungin, was first discovered as an endophyte from *Juniperus* in a mountain range near Madrid (Peláez et al. 2000) and seems to be frequently associated with this host plant in central Spain. However, subsequent studies revealed that this species is also present in other habitats. It was isolated from plant litter, and even from rock surfaces (Bills et al. 2004). This is a fair example that fungal endophytes are also capable of surviving outside their host and are only spending part of their life cycle in the plant host (Chethana et al. 2021a; Pem et al. 2021).

Metabolite extracts of the fungus exhibited very potent antifungal activity and the respective bioactive principle was soon identified as the triterpene glycoside enfumafungin. *In-vitro* studies of the compound demonstrated no antibacterial effects, but a broad-spectrum activity against fungi including clinically relevant pathogens such as *Candida albicans* and *Aspergillus fumigatus* in the range of the antifungal drug amphotericin B (Peláez et al. 2000).

### Biosynthesis of enfumafungin

**Fig. 6:**
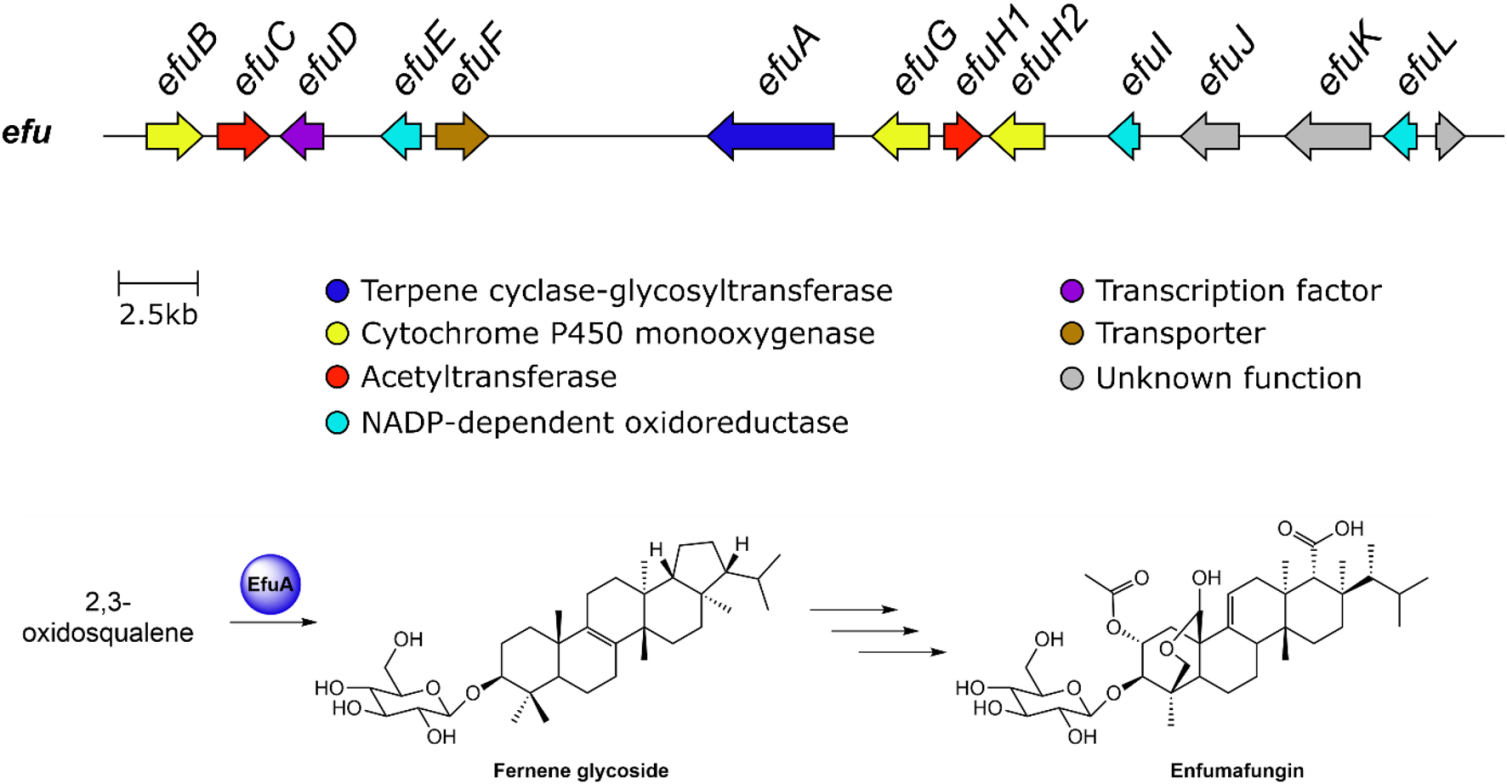
Reannotated enfumafungin biosynthetic gene cluster (*efu*) and hypothesized core step of the enfumafungin biosynthesis

Due to the medicinal relevance and unique structure of enfumafungin, the biosynthesis of the compound has been partially investigated (Kuhnert et al. 2018). For this purpose, the genome of producer strain ATCC 74,360 has been sequenced with Illumina technology and assembled into 129 contigs with a total lengths of 32.8 Mbp. Based on its structure enfumafungin was supposed to be derived from a triterpene cyclase with similarity to the lanosterol synthase. The presence of a sugar moiety, acetyl group, hemiacetal and carboxylic acid functionality further indicated that the biosynthetic locus should contain corresponding genes encoding for a glycosyltransferase, acetyltransferase, and multiple oxidative enzymes. Homology searches revealed a biosynthetic gene cluster (BGC) that contained all predicted genes, and which was termed *efu*. The core gene of the *efu* biosynthetic gene cluster (*efuA*) featured a very unusual triterpene cyclase that is fused to a glycosyltransferase Fig. 6. The structure of *efuA* was verified by cDNA sequencing. Phylogenetic analysis of the terpene synthase demonstrated that EfuA is distinct from known lanosterol synthases and forms an own lineage with homologs from a broad range of organism including bacterial squalene-hopene cyclases and uncharacterized fungal terpene cyclases. The latter are present across the major classes of the fungal kingdom (e.g., Agaricomycetes, Eurotiomycetes, Lecanoromycetes, Sordariomycetes). Most of the homologs did not feature a glycosyltransferase domain, but a subclade in the phylogeny containing EfuA included additional fusion proteins from unrelated fungi indicating that they are not rare (Kuhnert et al. 2018). The frequent occurrence of EfuA homologs in fungi is also in accordance with the structural diversity of glycosylated enfumafungin congeners isolated from various fungal sources. Examples are fuscosatroside from *Humicola fuscoatra* and *Chaetomium* sp., peniciside from *Penicillium* sp., hyalodendrosides from *Hyalodendron* sp., kolokosides from *Xylaria* sp. or unglycosylated congeners such as polytolypin from *Polytolypa hystricis*, and lobarialides and retigeric acids from *Lobaria* spp. All of these compounds share a common fernane core scaffold (sometimes difficult to recognize due to putative oxidative ring expansions) and are therefore also referred to as fernane-type triterpenoids (see discussion in Kuhnert et al. 2018).

The link of the *efu* biosynthetic gene cluster to the production of enfumafungin was shown by gene knockout studies of *efuA*, which led to abolishment of enfumafungin production and antifungal activity of the crude extracts. Based on the identified biosynthetic genes a biosynthetic route for enfumafungin was proposed. EfuA likely catalyzes the first reaction of the pathway by using 2,3-oxidosqualene to form the fernane core and subsequently also performs glycosylation via the glycosyltransferase domain. The fernene glycoside intermediate is further processed by a P450 monooxygenase and acetyltransferase to establish the acetyl moiety at C-2. The 5-membered ring is predicted to be expanded by another P450 monooxygenase and then cleaved by an undetermined enzyme to yield the carboxylic acid functionality. The hemiacetal part of the molecule is supposedly introduced by one or two P450 monooxygenases. The strong antifungal effects of enfumafungin also raised questions about the self-resistance of the producer organism. As the biosynthetic gene cluster encodes for a protein (*efuJ*) with homology to structural proteins of the fungal cell wall, the authors speculated that such an enzyme could be involved in a resistance mechanism (Kuhnert et al. 2018).

### Semisynthetic optimization

**Fig. 7:**
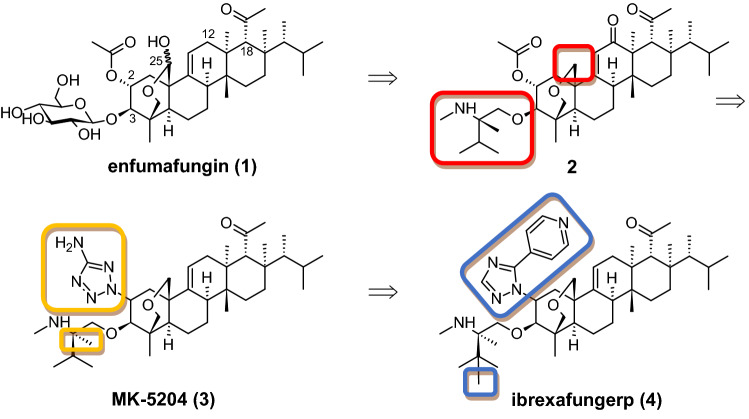
Development of ibrexafungerp (**4**) and comparison of chemical structures **1**–**4**. By modifying the chemical entities at C-2, C-3 and C-25 (illustrated by colored boxes), a series of semi-synthetic conversions culminated in the development of **4**

As already outlined above, enfumafungin (**1**) turned out to be the most potent out of four triterpenoid natural products with (1,3)-β-d-glucan synthase (GS) inhibitory activity (Onishi et al. 2000). The laboratories of Merck (initially) and (later) Scynexis established a medicinal chemistry program based on semisynthetic derivatization of the terpenoid natural product enfumafungin to optimize in vivo antifungal activity and oral absorption properties. Mainly chemical modification at C-2, C-3, C-12, C-18 and C-25 were accomplished, and the resulting analogs were evaluated for in vitro antifungal activity and for oral efficiency (Apgar et al. 2015). The chemical structures and the most important semisynthetic derivatives **1** – **4** are shown in Fig. 7.

A characteristic structural feature of enfumafungin (**1**) is the hemiacetal cyclisation of C-23/C-25 across the A ring on the β-face of the molecule, which gives rise to interconverting diastereomers at the anomeric carbon C-25. Besides hampering structure elucidation (Schwartz et al. 2000), this conversion can potentially cause chemical instability due to possible ring opening and oxidation reactions. Thus, the bridging hemiacetal of **1** was reduced to an ether moiety by ionic reduction with Et_3_SiH starting (Heasley et al. 2012). This conversion improved the stability of the bridging ring system and provided a single chemical entity, while at the same time a comparable antifungal activity was retained.

Since enfumafungin (**1**) did not exhibit an acceptable pharmacokinetic profile as a C-3 glycoside (Apgar et al. 2015), the β-d-glucose moiety was replaced by a chemically and metabolically stable system. After acidic methanolysis of the glycoside linkage, various chemical entities were installed and evaluated. 12-oxo-25-deoxy derivatives bearing an alcohol-amine-based side chain bound via an ether linkage to C-3 were efficacious in a candidiasis model when delivered orally. In course of the synthesis of derivative **2**, the incorporation of a quaternary stereocenter proximate to the basic amine of the C-3 enfumafungin side chain conferred improved oral activity in the target organ kidney assay (TOKA) murine model of disseminated candidiasis.

Lewis acid mediated nucleophilic displacements of the C-2 acetoxy group mediated by borontrifluoride diethyletherate proceeded with retention of stereochemistry at C-2. The observed stereochemistry of this reaction can be explained by stabilization of the intermediate carbocation by the proximal bridging ether exerting a neighboring group effect by blocking nucleophilic attacks from the upper hemisphere. The transformation proved to be versatile to displace the C-2 acetoxy group by various oxygen, carbon and nitrogen containing nucleophiles (Apgar et al. 2015). Combining an aminotetrazole substituent at C-2 with an aminoether substituent at C-3 produced a dramatic improvement in (1,3)-β-d-glucan synthase and antifungal potency, but resulted in a tenfold drop in oral exposure compared to the acetoxy group at C-2. This problem was solved by alkylating the amine of the aminoether substituent with a small alkyl group, which improved oral exposure and bioavailability while maintaining excellent (1,3)-β-d-glucan synthase and antifungal potency, culminating in the synthesis of MK-5204 (**3**) (Apgar et al. 2020).

During the course of further optimization (Apgar et al. 2021), an examination of various 3-alkyl and aryl-2-[1,2,4-triazole] substituents identified 3-(4-pyridyl)-2-[1,2,4-triazole] as the optimal replacement for the 3-carboxamide-2-[1,2,4-triazole] substituent of **3**. This 4-pyridyl substituent resulted in a fourfold improvement in antifungal activity in the presence of serum relative to MK-5204 (**3**) in conjunction with an 1.5 fold increase in oral exposure. Re-optimization of the alkyl substituents of the C-3 aminoether in the presence of the 3-(4-pyridyl)-2-[1,2,4-triazole] substituent at C-2, determined (*R*)-*tert*-butyl, methyl as the superior C-3 aminoether with a twofold increase in oral exposure over **3**, while sustaining the fourfold enhancement in antifungal activity in the presence of serum. The concurrent improvements in these two parameters resulted in a drastic improvement in the 7-day target organ kidney assay ED99 for ibrexafungerp (**4**) relative to MK-5204 (**3**). This development is an excellent example of how fungal metabolites, which are inherently not optimally designed for use in humans, can be turned into drugs by means of medicinal chemistry. It remains to be seen whether additional chemical modifications can improve pharmacokinetic parameters even further; with SCY-247 a very close structural derivative of **2** was just recently evaluated in a murine model of hematogenously disseminated *C. albicans* (Chu et al. 2021).

### Potential pharmaceutical use and market potential

Ibrexafungerp (**4**) has the potential to become an important drug for antifungal therapy with benefits over existing options, due to its oral efficacy and broad-spectrum antifungal activity, which includes echinocandin resistant isolates and *Candida* auris, for the treatment of multiple serious fungal infections, including vulvovaginal candidiasis (VVC), invasive candidiasis, invasive aspergillosis, and refractory invasive fungal infections (Davis et al. 2020).

After successful completion of phase III clinical trials for the treatment of vulvovaginal candidiasis (Jallow and Govender 2021), the FDA priority review of the new drug application was completed and ibrexafungerp (**4**) was approved on June 2^nd^, 2021 for the treatment of vaginal yeast infections under the trade name Brexafemme®. Thus, the first new class of antimycotics since more than 20 years has been introduced to the market very recently.

However, the development of **4** for the treatment of other, recurrent vulvovaginal candidiasis and invasive fungal infections is still ongoing with several clinical trials in phase II and III being under way (Lee 2021).

## The pleuromutilins, the latest antibacterial drug class that made it to the market, can now be produced by a sustainable biotechnological production process using a heterologous host!

**Fig. 8:**
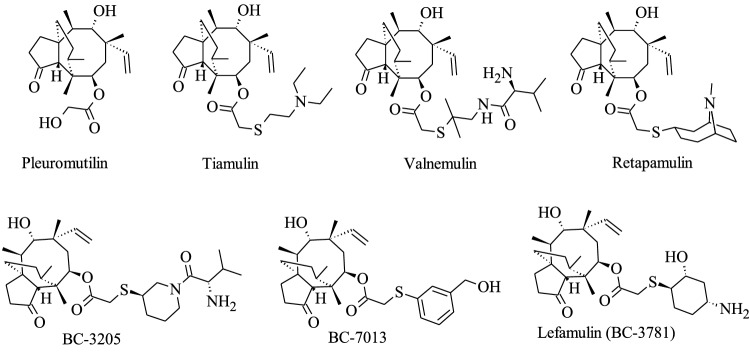
Chemical structures of pleuromutilin and its derivatives

Pleuromutilins (Fig. 8) are a well-known class of antibiotics from Basidiomycota. The naturally occurring pleuromutilin was isolated from *Clitopilus passeckerianus* (formerly named *Pleurotus passeckerianus*) already 70 years ago (Kavanagh et al. 1951). *Clitopilus* was introduced by (Kummer 1871) and is classified in Entolomataceae (Agaricales, Basidiomycota) (Co-David et al. 2009; Baroni and Matheny 2011). *Clitopilus* appears phylogenetically related to *Rhodocybe* as they share unique morphological features including pinkish basidiospores and evenly cyanophilic walls having 5–12 longitudinal ridges, and this was also corroborated by a phylogenetic analysis of the ITS region (Baroni and Matheny 2011; Kluting et al. 2014; Baroni et al. 2020). The taxonomy of pleuromutilin producers had remained obscure for a long time due to varying species concepts that also have affected many other groups of Basidiomycota (see Niego et al. (2021b) for the producers of strobilurins, which are another class of economically important secondary metabolites from Basidiomycota). Recent evidence has also revealed that they are chemotaxonomic markers for a certain clade of *Clitopilus*. A recent polythetic study, combining information in the literature concerning pleuromutilin production, morphological features and a phylogenetic analysis of the ITS region, has revealed that pleuromutilins producers are located in the section *Scyphoides* of *Clitopilus* (Hartley et al. 2009; Jian et al. 2020). *Clitopilus* species that are known to produce pleuromutilins or have the potential include for instance *C. passeckerianus*, *C. prunulus, C. scyphoides, C. pinsitus*, *C. fasciculatus,* and *C. hobsonii*. This has finally clarified that the compound is not produced by other genera of Basidiomycota (e.g., the occurrence in the genus *Omphalina* as reported by Hartley et al. 2009 and Jian et al. 2020).

Pleuromutilins display strong activities against most Gram-positive and some Gram-negative bacterial pathogens (Novak 2011; Paukner and Riedl 2017). However, the applications of natural products in vivo was limited due to insufficient metabolic stability, adverse gastrointestinal side effects, concerns on cardiac safety, and lack of intravenous tolerability (Paukner and Riedl 2017). Advances in the optimization of the pleuromutilins by medicinal chemistry, targeting the improvement of the pharmaceutical properties under maintenance of the potent antibacterial activity have subsequently led to interesting, new semisynthetic derivatives (Fig. 8) (Prince et al. 2013; Paukner and Riedl 2017). Finally, the semisynthetic pleuromutilin analog retapamulin (Fig. 8) was developed and marketed as the first approved antibiotic from Basidiomycota for treatment of skin infections of humans (Yang and Keam 2008). Other promising semisynthetic pleuromutilins like tiamulin (Fig. 8) and valnemulin (Fig. 8) were studied concurrently for use in veterinary medicine (Egger and Reinshagen 1976a, b). Tiamulin has become a successful drug for treatment of dysentery, pneumonia and mycoplasma infections in swine and poultry (Nahler and Nahler 2009). Valnemulin (Econor®) is also approved in veterinary medicine for therapy of swine dysentery and enzootic pneumonia in swine (European Medicines Agency 2019).

Lefamulin (Fig. 8), formerly known as BC-3781, is the first pleuromutilin type antbiotic that has been very recently approved for systemic therapy of bacterial infections in humans. It is now marketed as XENLETA™ for the treatment of community-acquired bacterial pneumonia (CAP) since August 2019 in the USA after approval by the U.S. Food and Drug Administration. In Europe, XENLETA™ was also approved of the marketing authorization application for the treatment of CAP throughout EU by the European Medicines Agency (EMA) since July 2020, which was announced by Nabriva Therapeutics plc (NASDAQ:NBRV) (Nabriva Therapeutics plc 2020). Based on the phase III clinical studies, almost 1,300 patients with CAP were treated with lefamulin by oral administration. The results exhibited the efficacy and general safety of lefamulin without any interferences to moxifloxacin (Alexander et al. 2019; File et al. 2019). Importantly, the drug can even be administered orally. Is is highly effective against *Chlamydia trachomatis*, *Mycoplasma genitalium, Neisseria gonorrhoeae* and even against multidrug-resistant isolates of important Gram-positive human pathogens (Bradshaw et al. 2017; Jacobsson et al. 2017). For instance, lefamulin displayed full activity against methicillin-susceptible and resistant *Staphylococcus aureus* and β-hemolytic streptococci (Sader et al. 2012; Paukner et al. 2013). Considering the fact that the pleuromutilins have a different molecular target than the conventionally used beta-lactams and other antibiotics that have been in use for many decades, there is now some hope that the pleuromutilins will remain effective for many years to come and their utility can be extended further by application in other scenarios of bacterial infections. For this purpose, however, the sustainable access to the compound must be improved further. The producer strains are basidiomycetes that grow relatively slowly and their fermentation at large scale is very difficult. It took several decades to make the pleuromutilin scaffold available in multi gram quantities to allow for the above mentioned drug development because the natural product needed to be harvested, isolated by preparative chromatography and subsequently modified by means of medicinal chemistry. Even for preclinical trials the derivatives had to be made available in multi gram scale. Fortunately, the recent technologies in –OMICS technologies, bioinformatics and biotechnological process developments have made it easier to tackle such challenges even with metabolites from slow growing organisms like Basidiomycota. The elucidation of the biosynthesis of pleuromutilins has provided a showcase on how this challenge may be tackled in the future even for many other fungal metabolites and it is therefore described in more detail further below.

The biosynthesis gene cluster (BGC) of pleuromutilin was recently found to contain seven genes: three cytochrome P450s (*Pl-p450-1*, *Pl-p450-2* and *Pl-p450-3*), one acetyltransferase (*Pl-atf*), one terpene cyclase (*Pl-cyc*), one geranylgeranyl pyrophosphate synthetase (GGS, *Pl-ggs*) and one short-chain dehydrogenase/reductase (SDR, *Pl-sdr*). This gene cluster was expressed heterologously in *Aspergillus oryzae*, giving a significant increase (over 2106%) in the production of pleuromutilin (Bailey et al. 2016; Alberti et al. 2017; Yamane et al. 2017). The heterologous expression of the gene cluster in *Aspergillus* has been a hallmark in fungal biotechnology, which could lead to more interesting metabolites from Basidiomycota becoming available for intensified studies in the future. These organisms are extremely creative in particular regarding the production of unique terpenoids (Sandargo et al. 2019; Gressler et al. 2021). However, a lack of access to larger quantities has hitherto often precluded the broad biological characterization of these compounds, in particular if they were derived from fruiting bodies of species that cannot easily be cultured, or by ectomycorrhizal or other slow-growing species. The example of pleuromutilin and its heterologous production could therefore well have marked a change of paradigms and may ultimately lead to many additional exploratory and clinical candidates for the development of antibiotics and other pharmaceutical drugs. This could be an essential part of future strategies to tackle the challenge of antimicrobial resistance (Miethke et al. 2021).

## A newly discovered immune disorder explaining severe mycoses

Chronic and highly mutilating fungal infections in otherwise healthy-appearing patients have long remained enigmatic. The classical case of a patient with a destructive, finally fatal infection caused by the otherwise harmless plant pathogen *Mycocentrospora acerina* (Lie-Kian-Joe et al. 1957) is illustrative. However, it was recently discovered that most patients with such infections are not perfectly healthy, but have one of a gamut of inherited immune disorders (Gross et al. 2006; Hsu et al. 2007). For fungi, mutations in the signaling protein CARD9 (caspase recruitment domain-containing protein 9) are particularly relevant. Immunological investigations have shown that most fungal infections are controlled by the innate immune system via C-type lectin receptors (CLRs). Fungal β-glucans and mannans are recognized by the hosts’s pattern recognition receptors (PRRs) Dectin-1, Dectin-2 and Dectin-3. A cascade is triggered via the signaling protein CARD9 which stimulates the release of pro-inflammatory cytokines such as Interleukin 6 (IL-6) and Tumor Necrosis Factor alpha (TNF-α) by activating macrophages (Drummond et al. 2011, 2018).

During the last decade, numerous chronic and severe fungal infections proved to have associations with homozygous mutations in the *CARD9* gene interfering with its function. In several cases, a familial relationship of patients with similar infections was revealed (Boudghène-Stambouli et al. 2017) indicating that the disorder is inherited. CARD9 mutations impair the resistance against parasites and fungi. Vaezi et al. (2018) were the first to show that not all fungi, but only particular groups were concerned: predominantly *Candida*, dermatophytes and black fungi, while the otherwise very common opportunist *Aspergillus fumigatus* remained absent (Zhang et al. 2020a, b, c, d). Song et al. (2021) showed that mutations in *CARD9* each led to susceptibility to either *Candida*, dermatophytes, or black fungi, while only a fraction of the mutations was associated with more than one of the three fungal groups. This suggests a fine-tuned connection between fungus and the host’s signaling system.

*Candida* and dermatophytes are common colonizers of healthy individuals and thus are likely to expand upon immune weakness. In contrast, black fungi are unexpected agents of disease, since they are environmental and are uncommonly found on humans. Nevertheless, black fungi in *Phialophora* are particularly pronounced in *CARD9*-related infections. *Phialophora* species are environmental fungi and cause opportunistic infections only occasionally (Song et al. 2021). Quite probably, all published severe infections by *Phialophora* and other black fungi concerned patients with *CARD9* defects. As yet unproven examples of such infections are cases by *Exophiala dermatitidis* (Shimazono et al. 1963; Chang et al. 2000), *E. spinifera* (Dai et al. 1987; Singh et al. 2012a, b), *Cladophialophora devriesii* (Mitchell et al. 1990), *Phialophora tarda* (Hofman et al. 2005), and *Veronaea botryosa* (Matsushita et al. 2003); these were all published without awareness of the *CARD9* immune disorders. Some were indeed recognized retrospectively as being related to mutations in the *CARD9* gene (Bonifaz et al. 2013).

For as yet unknown reasons, nearly all *CARD9*-related disseminated infections by black fungi are found in East Asia (Lanternier et al. 2015). Some of the species are known as colonizers of domesticated locations, such as *Exophiala dermatitidis* in bathing facilities (Matos et al. 2002). However, despite the likely inhalation via aerosols during bathing, very few *CARD9* infections by this fungus are known in the Western world. In contrast, cases of destructive disseminated infections by *E. dermatiitidis* and related black fungi are encountered in East Asia.

Most patients exhibit cancerous expansion of skin tissue with acanthosis and hyperkeratosis, and deformations with local loss of tissue. Some similarity to chromoblastomycosis has been noted. This chronic skin disease is unique to black fungi and provokes cancerous elevations of skin tissue. Excessive acanthosis and hyperkeratosis is not unique to chromoblastomycosis. Patients have skin deformations due to excessive expansion of (sub)cutaneous tissue. Also, this disease might be *CARD9*-related. Several members of the above genera were thought to be severe pathogens and were classified in the highest biosafety category, but possibly the main trigger for these infections are immune defects of the hosts. One of the *CARD9* mutations was associated with severe cases of chromoblastomycosis, which links both diseases types of disease sharing acanthosis.

**Fig. 9:**
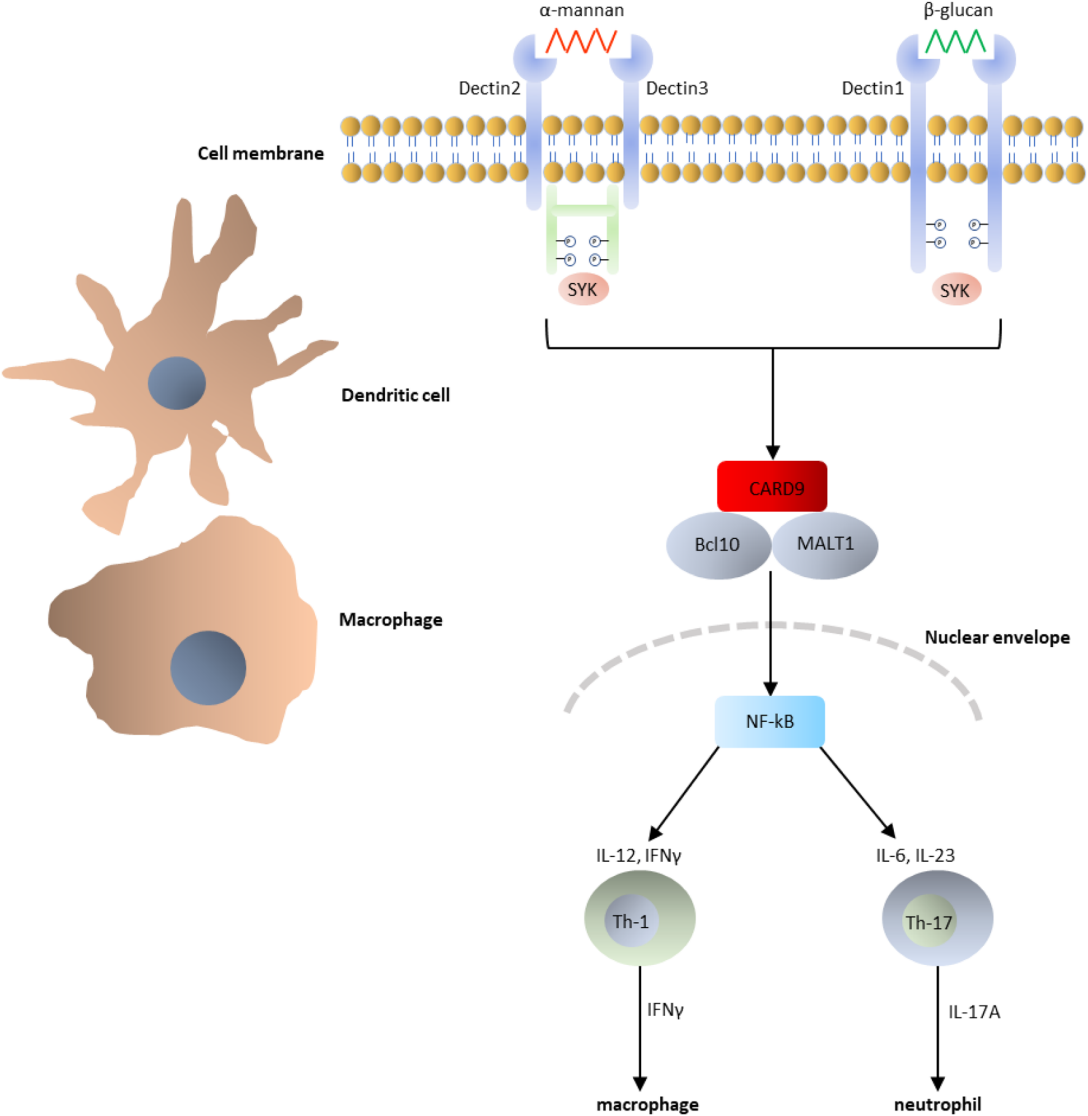
Diagram of signal transduction pathway of fungal carbohydrate antigens by the human immune system. *Bcl* B-cell lymphoma protein 10, *CARD9* caspase recruitment domain-containing protein 9, *IFN* interferon, *IL* interleukin, *MALT1* mucosa-associated lymphoid tissue lymphoma translocation protein 1, *NF-kB* nuclear factor kappa-light-chain-enhancer of activated B cells, *P* phosphor, *SYK* tyrosine-protein kinase, *Th* T-helper cell. CARD9/Bcl10/MALT1 is a central proinflammatory signalosome in innate immune cells. Modified after Drummond et al. (2011)

These findings have revolutionized the understanding of severe and chronic fungal diseases. Possibly, many of the heavily mutilating infections by opportunistic species are related to *CARD9* mutations. For example, the *Mucor* species causing severe and chronic facial infections, *M. irregularis*, which deviates from all other Mucorales that cause acute infections in preconditioned patients, was also found to have a link with *CARD9* (Wang et al. 2019). Other deficiencies have been reported e.g. in the transcription factors STAT1 (van de Veerdonk et al. 2011) and GATA2 (Egenlauf et al. 2015). Apparently, the severity of these infections is largely due to the compromised host, and less to the virulence of the fungus. For this reason, several black fungi, including *Cladophialophora devriesii*, have been moved from biosafety (BSL) level 3 to BSL-2 (de Hoog et al. 2020). Due to the dysfunctional human side of the host/fungus interaction, antifungal treatment is less effective than might be expected on the basis of in vitro susceptibility test results. Black members of Chaetothyriales are generally susceptible to all commonly used antifungals, but patients with homozygous *CARD9* mutations appear extremely difficult to treat. Antifungal compounds are effective for short while, but then the fungus takes over again, leading to slow but unstoppable disfigurement. Alternative treatment options may be the application of Granulocyte–Macrophage Colony Stimulating Factor (GM-CSF) (Gavino et al. 2014) or hemopoietic stem cell therapy (Queiroz Telles et al. 2019). Further research is needed before these methods can utilized with safe and certain outcomes (Fig. 9).

## Advances in the molecular regulation of the biosynthesis of mycotoxins in *Fusarium*: focus on chromatin structure

Mycotoxins are toxic specialized metabolites produced naturally by certain filamentous fungi. They represent a major issue for the agricultural sector worldwide, due to their frequent and sometimes high occurrence and the difficulties to mitigate their presence. To date, there is no existing cropping strategy that is fully effective in limiting mycotoxin contaminations and certifies compliance with official limits (set in Europe by the EC regulation number 1881/2006 rev. 2009). The problem may soon become more important as a result of changes in production practices and climate. In addition, these considerations may also apply to a wide range of unknown (or as yet unstudied) secondary metabolites.

*Fusarium* is one of the most widely recognized genera of plant pathogenic fungi that produce important mycotoxins. Among *Fusarium* species, *F. fujikuroi* Nirenberg (= *Gibberella fujikuroi* (Sawada) Wollenweber) and *F. graminearum* (= *Gibberella zeae*) are causal agents of major plant disease and responsible of the contamination of various crops with mycotoxins. *Fusarium fujikuroi* is associated with the *bakanae* (‘foolish seedling’) disease of rice (Hori 1890). Contamination of rice with this taxon is widely distributed in all rice-growing countries and occurrence of *bakanae* has even increased in the recent years due to environmentally-friendly rice cultivation (Jeon et al. 2013). As rice is a staple crop with an estimated 500 million tons produced in 2014/2015, yield reductions due to plant diseases have a great impact on food and feed safety, making research on this taxon of broad interest. *Bakanae* symptoms are caused by the ability of the fungus to produce and secrete gibberellic acid (Yabuta and Hayashi 1939). Besides gibberellic acid, *F. fujikuroi* produces a huge arsenal of other secondary metabolites including various toxins accumulating during infection, *e.g.,* fusaric acid, fusarin C or beauvericin (Niehaus et al. 2017). *Fusarium graminearum* is a pathogen causing disastrous “Fusarium Head Blight” outbreaks on wheat across the world (Dean et al. 2012). *Fusarium graminearum* is responsible for the production and accumulation of type B trichothecenes (mycotoxins), such as deoxynivalenol, as well as other secondary metabolites in cereal grains, during growth. Trichothecenes are particularly stable and resistant to agri-food processing, ending up in finished products. Beside its toxic properties for humans and animals upon ingestion, deoxynivalenol also plays a role in aggressiveness of the fungus on wheat (Maier et al. 2006).

Understanding the factors involved in crop infection and secondary metabolite production is a pre-requisite for the elaboration of durable, environment-friendly, strategies to control crop health. It is therefore important to increase knowledge regarding the mechanistic clues that can explain the regulation of mycotoxin production. This regulation is likely to operate on different regulatory levels associating pathway-specific and global regulators, signal transduction pathways, and epigenetic control. During the last decade, the most novel and significant insights have concerned the impact of chromatin structure changes on mycotoxin biosynthesis. This chapter proposes a synopsis of most striking advances on the subject.

### Role of chromatin in the regulation of fungal secondary metabolism

In eukaryotes, including fungi, genomic DNA wraps around histone protein octamers to form nucleosome chains. All eukaryotes possess four canonical histone proteins – H2A, H2B, H3, and H4 – that constitute the histone octamer, each of them being present in two copies. Additionally, they can possess variant copies of these histones, whose types and numbers differ per species. The histones constituting nucleosomes can carry modifications added post-translationally (e.g., acetylation, methylation) or be replaced by variant histones that influence the overall level of tightness of the wrapping. This organized combination of DNA with histone proteins is called chromatin. Heterochromatin corresponds to genomic regions that are tightly packed, by opposition to euchromatin that refers to more relaxed structures. A common paradigm is that euchromatic territories are the places where active transcription of cellular genes can take place. The organization of chromatin is not static, and remodeling events play important roles in gene regulation. In fungi, gene clusters encoding for secondary metabolites are silent in repressed chromatin (heterochromatin) when production is non-favorable, and can be readily activated during development or infection processes (Strauss and Reyes-Dominguez 2011). There is now profound evidence that many development-related processes, such as rapid response to environmental changes or expression of secondary metabolite genes, are subjects of epigenetic control via chromatin structure changes.

Histone H3 lysine 4 (H3K4) and H3K36 methylation marks have been described as hallmarks of euchromatin in budding and fission yeasts as well as in higher eukaryotes (Rando and Chang 2009; Wagner and Carpenter 2012). Indeed, the H3K4me marks are largely located to euchromatic regions in both *Fusarium fujikuroi* and *F. graminearum* (Connolly et al. 2013; Wiemann et al. 2013). Deletion of *CCL1*, a component of COMPASS (complex associated with Set1) and required for full H3K4me3, resulted in an altered secondary metabolite profile in both taxa (Studt et al. 2016a, b). H3K4me2/3 deposited and removed by Set1 and Kdm5, respectively, play a role in development, secondary metabolite production and pathogenicity (Liu et al. 2015a, b; Janevska et al. 2018a, b). In *F. fujikuroi*, H3K36me3 covers whole chromosomes. Notably, two genes are involved in deposition of this mark, *i*.*e*., *ASH1* (H3K36me3 at subtelomeric regions) and *SET2* (H3K36me3 at euchromatic regions) (Janevska et al. 2018a, b). Deletion of the respective genes *ASH1* and *SET2* resulted in the de-regulation of secondary metabolism in *F. fujikuroi*. Notably, neither in case of H3K4me3 nor H3K36me3 could the observed effects could be directly associated with the respective histone modifications at the analyzed secondary metabolite gene clusters. Conversely to activation, two methylation marks, i.e., H3K9me3 and H3K27me3, have been associated with repression of secondary metabolite gene expression in *F. graminearum* (Connolly et al. 2013; Reyes-Dominguez et al. 2012). For H3k27me3, a direct association with secondary metabolite gene clusters has been shown. Consequently, deletion of the involved histone methyltransferase (Kmt6) induces expression of otherwise silent secondary metabolite genes (Connolly et al. 2013).

In *F. fujikuroi* the ortholog of *KMT6* appears to be essential (Studt et al. 2016a, b), but similarly to *F. graminearum*, down-regulation of *KMT6* by RNA interference resulted in up-regulation of several otherwise silent secondary metabolite genes, a phenotype that was accompanied by reduced H3K27me3 levels at the respective gene loci and the production of novel compounds (Studt et al. 2016a, b). Notably, H3K9me3 established by Kmt1 appears to be essential for biosynthesis of fusapyrone in *Fusarium mangiferae* associated with mango malformation (Atanasoff-Kardjalieff et al. 2021). Another histone mark that has received little attention in filamentous fungi is H4K20me3 associated with gene silencing in higher eukaryotes (Kourmouli et al. 2004; Schotta et al. 2004). In *F. fujikuroi* and *F. graminearum* one protein, Kmt5, writes mono-, di- and trimethylation of H4K20me, and its loss distinctly affects secondary metabolite biosynthesis with the most pronounced effects on fusarin biosynthesis in *F. fujikuroi* and zearalenone biosynthesis in *F.graminearum* (Bachleitner et al. 2021).

Next to histone methylation, histone acetylation has been shown to greatly influence secondary metabolite gene regulation in both fungi. For example, the histone acetyltransferase Gcn5, a member of the SAGA complex, is responsible for the acetylation of several histone 3 lysine residues in *F. fujikuroi*, e.g., H3K4, H3K9, H3K18, and H3K27 (Rösler et al. 2016). Deletion of *GCN5* affected the transcription of 28 out of 47 putative secondary metabolite gene clusters. Similarly, the histone deacetylases Hda1 and Hda2 are involved in secondary metabolite gene regulation in this fungus (Studt et al. 2013). While several known secondary metabolites were shown to be de-regulated upon deletion of either *HDA1* or *HDA2*, deletion of *HDA1* resulted in the activation of a previously silent secondary metabolite, which was later on identified as beauvericin (Niehaus et al. 2016). Similarly, the ortholog of *HDA2*, *HDF1* in *F. graminearum*, seems to be involved in the activation as well as the repression of secondary metabolite genes (Li et al. 2011). The intervention of chromatin control through specific changes in histone marks thus appears today as a major mechanism that controls secondary metabolite biosynthesis. In fact, chromatin could be a new and relevant target to generate novel strategies to control mycotoxin accumulation in grains.

### Secondary metabolism and the histone variant H2A.Z

H2A.Z is a histone variant that makes up 5 to 10% of the total H2A protein in most organisms examined to date. Abundances increase when cells exit the cell cycle and no longer replicate their DNA, such as during development (Piña and Suau 1987). H2A.Z has been linked to a wide variety of different sometimes contradictory nuclear functions, including transcriptional activation, transcriptional repression, RNA Polymerase II elongation, heterochromatin, anti-silencing, cell-cycle control, DNA replication, DNA damage repair, chromosome segregation, and genome integrity (Chen and Ponts 2020). The function of H2A.Z appears to be essential in a number of organisms, including *F. fujikuroi* and *F. graminearum* (Sevilla and Binda 2014; Chen et al. 2020).

H2A.Z has been mapped genome-wide in a variety of eukaryotes. This histone variant is mainly found around transcriptional start site of genes and at enhancer sites, likely regulating transcription, is mutually exclusive with DNA methylation, and can be modified post-translationally (Sevilla and Binda 2014). In particular, the N-terminal tail of H2A.Z can be acetylated by the NuA4 and the SAGA histone acetyltransferase complexes (Babiarz et al. 2006; Keogh et al. 2006; Mehta et al. 2010). Conversely, H2A.Z deacetylation was shown to involve the Hda1 protein deacetylase (Mehta et al. 2010). High-resolution chromatin immunoprecipitation (ChIP) experiments in a number of model organisms have revealed that H2A.Z preferentially occupies nucleosomes that flank gene promoters and is particularly enriched at the + 1 nucleosome as well as at the − 1 and − 2 nucleosomes thereby flanking nucleosome-depleted regions at the transcriptional start sites (Talbert and Henikoff 2010). These nucleosomes also exhibit rapid, replication-independent turnover, which is thought to function in erasing histone marks, preventing the spread of chromatin states, and ensuring general plasticity of the epigenome. In budding yeast, nematodes and plants, H2A.Z occupancy around promoters is correlated with non-transcribing genes ‘poised’ for transcription. Similarly, in yeast, H2A.Z is involved in “transcriptional memory”, *i.e.*, the priming for fast reactivation of repressed genes involving perinuclear localizations. However, in flies and mammals, promoter H2A.Z occupancy appears to correlate more with actively transcribed genes, although studies in mouse embryonic stem cells revealed that H2A.Z preferentially occupies the promoters of genes that are poised to direct development and differentiation when activated. In mice, inhibiting H2A.Z expression results in increased and more stable nucleosome occupancy at regulatory regions, decreased methylation of H3K4 and H3K27 at promoters and enhancers, and the de-repression of developmental target genes. In budding yeasts, H2A.Z is specifically deposited near or within heterochromatin, where it serves as an anti-silencing factor. Here, its deletion results in extended spreading of silent chromatin inward from the telomeres. This effect can be suppressed by the additional deletion of genes encoding silencing factors themselves. Indeed, this function may act globally, in parallel with the Set1 histone H3 methyltransferase, to prevent large-scale aberrant distribution of silencing factors (Venkatasubrahmanyam et al. 2007).

The importance of histone modifications in secondary metabolite gene regulation is well-accepted and has been studied in several fungi over the last years, including also *F. fujikuroi* and *F. graminearum* (Chen and Ponts 2020). Yet, nothing is known regarding the influence of histone variant deposition on secondary metabolite gene regulation in fungi. H2A.Z is involved in the regulation of adaptive gene clusters in other organisms, including the virulence (vir) cluster in the malaria parasite (*Plasmodium falciparum*) (Petter et al. 2013), the Hox gene cluster in animals (Creyghton et al. 2008) as well as the thalianol and marneral gene clusters in *Arabidopsis thaliana* (Nützmann and Osbourn 2015). In *S. cerevisiae*, H2A.Z is required for the coordinate expression of the DAL cluster, a catabolic gene cluster involved in allantoin utilization (Wong and Wolfe 2005), and deposition of H2A.Z in euchromatic regions together with trimethylation of H3K4 prevents Sir2 spreading into these regions, thereby indirectly contributing to proximal telomeric gene silencing (Venkatasubrahmanyam et al. 2007; Meneghini et al. 2003). Thus, it is likely that H2A.Z also plays a role in the regulation of the fungal secondary metabolism. In a general manner, H2A.Z and H3K4me3 often co-localize at active sites of transcription, and loss of Set1 resulting in a complete loss of H3K4me leads to an altered secondary metabolite profile in both fungi (Liu et al. 2015a, b; Janevska et al. 2018a, b). Notably, in *S. cerevisiae*, both H2A.Z and SET1 are involved in genome-wide anti-silencing by preventing ectopic, Sir2-dependent silencing of genes across euchromatin (Venkatasubrahmanyam et al. 2007). H2A.Z and H3K4me3 could, here, be acting together. The relationship between their respective depositions on the genome remains to be defined.

### Conclusion

Many factors influence the production of toxins potentially implying the intervention of various regulatory genes in response to various environmental factors. In the recent years, it became evident that chromatin structure plays a role in the regulation of secondary metabolism in filamentous fungi. The intervention of chromatin control, through specific histone modifications, appears today as a major mechanism of controlling mycotoxin biosynthesis in fungi. Dynamic changes of chromatin structure allow the expression of secondary metabolite-related genes hitherto silent as optionally embedded in repressive chromatin.

## Successful application of CRISPR-Cas9 in medical mycology

Being eukaryotic pathogens, the kingdom Fungi shares similarities with human cells (Rodrigues and Nosanchuk 2020; Nargesi et al. 2021). Fungal pathogens often cause chronic diseases, and with prolonged disease duration, they tend to mutate, causing them detrimental to humans (Hyde et al. 2018a). The highly variable trophism exhibited by fungal pathogens allows them to infect a wide range of cells (Rodrigues and Nosanchuk 2020). The ability of these pathogens to infect multiple tissues while undergoing morphogenetic shifts makes fungal diseases differ significantly from other infections (Li and Nielsen 2017). Over 600 fungal pathogens that may cause diseases in humans have been reported so far, and among them, *Aspergillus*, *Candida*, *Cryptococcus* and *Pneumocystis* species are the most common (Taylor et al. 2001; Morio et al. 2020; Rodrigues and Nosanchuk 2020). Fungal infections in humans or mycoses vary from mild to life-threatening, with various symptoms. Invasive mycoses were acknowledged only in the 1980s, and till then, fungi were underappreciated as human pathogens (Nucci and Marr 2005). Superficial fungal infections are the most common aspect, affecting almost two billion people worldwide and are easy to treat (Cole et al. 2017). Despite having lower disease incidence, invasive fungal infections can be life-threatening and associated with unacceptably high mortality rates (Janbon et al. 2019; Morio et al. 2020). However, this picture has changed with the increasing population of immunocompromised individuals (Enoch et al. 2017; Patel et al. 2017). Therefore, with the broadening of the susceptible population, the frequency of invasive mycoses increases resulting in a death toll of about one and a half million people annually (Brown et al. 2012; Bongomin et al. 2017). Even the commensal fungi become lethal to immunocompromised patients with immunosuppressive diseases, such as HIV and neutropenia or to the ones undergoing treatment for severe diseases such as cancer and pancreatitis (Iliev and Underhill 2013; Fisher et al. 2020), making fungal infections a significant global public health problem (Li and Nielsen 2017).

Whether invasive or superficial, successful control of fungal diseases depends highly on the timely diagnosis, effective antifungal therapy and reversal of predisposing factors (Riley et al. 2016). However, diagnosis and treatment remain challenging tasks for these fungal infections (Bruni et al. 2019). The situation is further complicated with fungi exhibiting intrinsic resistance to the majority of the routinely used antifungal agents, limiting the possible therapeutic options (Riley et al. 2016; Scorzoni et al. 2017). Therefore, diagnostic tools and antifungal drugs with improved efficiency are needed (Janbon et al. 2019). Due to these reasons, there is a necessity to advance efficient genetic manipulation techniques and an urgency to search for new antifungal targets. However, to achieve this, a deeper understanding of the epidemiology of the fungal pathogens, their interactions with the hosts, potential virulence factors and novel biomarkers are vital. Genetic manipulation tools have been utilized to decipher drug resistance mechanisms and the virulence potential in selected fungi, in a targeted and defined manner. However, these tools are tedious, time-consuming and difficult to use in fungi, specifically for fungi that lack a sexual cycle (Alberts et al. 2002). Conventional genome editing techniques, such as RNA interference, various artificial nucleases, such as zinc finger nucleases and transcription activator-like effector nucleases, have been used to manipulate fungal genomes (Meyer 2008; Weinthal et al. 2010; Carroll 2011; Arazoe et al. 2015; Chandrasegaran and Carroll 2016; Sarkari et al. 2017; Wang et al. 2017). Diploid genomes, lack of sexual cycle, absence of natural plasmids, lack of cloning vectors, scarcity of dominant markers for screening purposes, coupled with fewer numbers of transformants resulting from the prevailing techniques (Samaranayake and Hanes 2011; Defosse et al. 2018; Morio et al. 2020), hampered most of the research efforts in fungi. A ground-breaking, novel genome-editing technique clustered regularly interspaced short palindromic repeats (CRISPR- CRISPR associated protein 9/Cas9) was introduced in the last decade (Mojica et al. 2005), overcoming the drawbacks of the previous techniques and revolutionizing the genome editing arena.

The CRISPR system was first discovered as an adaptive immune system in bacteria (Barrangou et al. 2007) which was later adopted for editing genomes in other organisms, especially in mammalian cell lines and yeasts. It is now being used as an efficient tool in molecular biology. There are different types of CRISPR/Cas systems but the most commonly used is the type II CRISPR/Cas9 system from *Streptococcus pyogenes* (Marraffini and Sontheimer 2008; Nargesi et al. 2021). The CRISPR/Cas9 system introduces stable and heritable changes into the genome via precision insertions and deletions (Wu et al. 2014). This system consists of two main working components, Cas9 endonuclease and a single-guide RNA (sgRNA) (Cui et al. 2018). Cas9 endonuclease introduces a double-stranded break, three base pairs upstream of the protospacer adjacent motif (PAM), which is a small chimeric motif present within the target sequence and facilitates the specific targeting of the Cas9 nucleases (Karvelis et al. 2015). The sgRNA is the fusion between the CRISPR-RNA (crRNA) and the *trans*-activating crRNA (tracrRNA) that provides the specificity and the scaffolding/binding ability to Cas9 (Doench et al. 2014). The resulting DSB of this process is repaired either by non-homologous end joining (NHEJ) facilitated by the natural repair mechanism of the cell or homology-directed repair (HDR) facilitated by the donor template (Morio et al. 2020).

The *Saccharomyces cerevisiae* genome is the first for which CRISPR/Cas9 has been applied (Dicarlo et al. 2013). Since then, it has been successfully adapted to many clinically, agriculturally and industrially important fungi for functional characterization and breeding purposes (Song et al. 2019). The system makes it possible to perform genetic changes, inactivate target genes, replace defective genes with healthy ones, and alter gene expression via deletions, mutations, barcoding and tagging performed throughout the genome or in specific sites. Apart from understanding the virulence factors and disease progression, gene-editing in human pathogenic fungi can be utilized in developing new antifungal drugs (Song et al. 2019). The multiplexing capabilities of the CRISPR system are used to develop fungal cell factories that produce medically important compounds and other metabolites (Nielsen et al. 2017). Human pathogenic fungi, such as *Aspergillus* spp., *Blastomyces dermatitidis, Candida albicans*, *Cryptococcus* spp., *Fusarium* and *Malassezia*, have been successfully edited using this system (Vyas et al. 2015; Arras et al. 2016; Wang et al. 2016; Fan and Lin 2018; Song et al. 2018). In this study, we discuss the application of this biotechnological breakthrough on medically important fungi that has the potential to revolutionize the medical field.

### Applications of the CRISPR/Cas9 technology in medical mycology

In a time when novel approaches are urgently needed to overcome fungal diseases, the CRISPR/Cas9 system has successfully manipulated target genes in human pathogenic fungi, including yeasts (*Candida* and *Cryptococcus* species) and molds (*Aspergillus* species). This section discusses how CRISPR/Cas9 technology is used to limit the susceptibility to fungal diseases and its therapeutic potential using the FDA approved clinical trials.

### Understanding human-fungal pathogen interactions

Pathogenic fungal interactions with human tissues influence the establishment of fungal diseases (Tronchin et al. 2008). As the adhesion to the human tissues is important for disease initiation, understanding fungal interactions with the human is a prerequisite for controlling and treatment purposes. Many studies have employed CRISPR/Cas9 to study genes responsible for fungal virulence (Gauthier et al. 2012; Min et al. 2016, 2018; Lombardi et al. 2017; Shapiro et al. 2018; Umeyama et al. 2018; Ballard et al. 2019; Bruni et al. 2019). This system generates multiple, parallel, genome-wide mutations of targeted genes and tests their function in response to fungal diseases (Mans et al. 2015; Sharon et al. 2018). Most of these studies have been focused on understanding the interactions of major human pathogenic fungal lineages, such as *Candida* species, *Cryptococcus neoformans*, *Aspergillus fumigatus* and Mucorales (Vyas et al. 2015; Arras et al. 2016; Min et al. 2016, 2018; Wang et al. 2016; Zhang et al. 2016; Al Abdallah et al. 2017; Huang and Mitchell 2017; Lombardi et al. 2017; Nguyen et al. 2017; Nagy et al. 2017; Fan and Lin 2018; Shapiro et al. 2018; Umeyama et al. 2018; Ballard et al. 2019; Bruni et al. 2019; Wensing et al. 2019). They not only contributed to manipulate fungal pathogens, but also develop and assess different CRISPR/Cas9 delivery strategies, their transient or permanent expression systems, leading to the evolution of CRISPR/Cas9 technology (Table 1) (Vyas et al. 2015; Min et al. 2016, 2018; Arras et al. 2016; Wang et al. 2016; Zhang et al. 2016; Al Abdallah et al. 2017; Huang and Mitchell 2017; Lombardi et al. 2017; Nguyen et al. 2017; Nagy et al. 2017; Fan and Lin 2018; Shapiro et al. 2018; Umeyama et al. 2018; Ballard et al. 2019; Bruni et al. 2019; Wensing et al. 2019). For instance, before the CRISPR/Cas system, which radically increased HDR in fungal species such as *Cryptococcus* was introduced, it was not possible to produce stable transformants in many clinically important fungal strains. Further, some species such as *C*. *neoformans* could not be successfully transformed with chemical methods nor electroporation. Based on the rapid progress achieved, CRISPR and CRISPR/Cas9 were selected as the Science’s Breakthrough of the Year 2015 (Kim et al. 2017).

**Table 1:** Studies conducted on human fungal pathogens using the CRIPSR/Cas9 system and its developments

Targeted genes and mutant phenotypes	Cas9 and genomic RNA expression modules	Improvement	Reference
***Candida albicans/C. parapsilosis*:** Causes Candidiasis in immunocompromised individuals			
*ADE2, CDR1*, *CDR2:* Duet system increased mutation efficiency by 20–40% while the solo system targets 60–80%	**Cas9:** *Candida*/*Saccharomyces* codon-optimized Cas9 (CaCas9) / the ENO1 promoter(p) **gRNA:** RNA polymerase III promoter SNR52	Generate homozygous mutations during a transformation by both duet and solo system	Vyas et al. (2015)
*ADE2*: Easily visible red phenotype observed in mutants (homozygous mutants)	**Cas9:** ENO1p:CaCas9:CYC1t **gRNA:** RNA polymerase III promoter SNR52P	Introduced a transient CRISPR/Cas9 for efficient gene deletion	Min et al. (2016)
*CDR1*, *CDR2*: increased sensitivity to the clinically useful azole antifungal agents in the mutants	**Cas9:** ENO1p:CaCas9:CYC1t **gRNA:** RNA polymerase III promoter SNR52	CRISPR-mediated marker excision (CRIME)	Huang and Mitchell (2017)
*ADE2, URA3, WOR1, WOR,* CZF1: more than 50% integration efficieny with 80% single gene deletions	**Cas9:** US-pENO1:Cas9:NAT **gRNA:** NAT-pSNR52-gRNA-DS	Develop a marker-less system without molecular cloning: LEUpOUT system for marker recycling	Nguyen et al. (2017)
*ADE2*, *CPAR2_101060*, *URA3*: 100% transformation efficiency across 20 clinical isolates	**Cas9**: TEF1p:Cas9:TEF1t **gRNA:** pCpSNR52-sgRNA:SUP4t, cpGAPDHp:HH-sgRNA-HDV: GAPDHt	Ability to edit any number of target genes in a single transformation step	Lombardi et al. (2017)
*NDT80*, *REP1*, *RON1*: multiple deletions were successfully constructed	**Cas9:** CRISPR-Cas9 system by using a SAT1-FLP system **gRNA:** SNR52P/TENO1	Enhance the understanding of target genes (single or in combination) in virulence	Min et al. (2018)
Antifungal efflux and biofilm adhesion factors: generated the two large pairwise gene deletion mutants	**Cas9**: CAS9 and two sgRNAs integrated at the *NEUT5L* locus in a haploid cell **gRNA:** 5’-homology arm–SNR52P-gRNA1–gRNA2-3’ homology arm	Develop a gene drive array system to generate combinatorial deletion mutants	Shapiro et al. (2018)
*ADE2*: 20-fold repression of the target gene	**Cas9**: ACT1p:dCas9:ACT1t **gRNA**: SNR52p:gRNAtail	Demonstrated a functional CRISPR system for the repression of gene expression	Wensing et al. (2019)
***Aspergillus fumigatus*:** Causes Aspergillosis in immunocompromised individuals			
*pks*, *cnaA*: Albino colonies due to reduced melanin production achieved with 95–100% mutation rate	**Cas9**: Gpdap:3xFLAG-NLS-Cas9-NLS:TRPCt **gRNA**: U6-3-gRNA	Established the system for mutagenesis using MMEJ process	Zhang et al. (2016)
*PKSP*: close to 100% gene deletion efficiency	**Cas9:** Alt-R-CRISPR-Cas9 components from integrated DNA technologies **gRNA:** cr5 = pksP and cr3 = pksP	Elimination of strain construction step by introducing in vitro assembly of Ribonucleoproteins	Al Abdallah et al. (2017)
*CYP51A*: site-directed mutagenesis successfully established using CRISPR-Cas9 system	**Cas9**: Cas9-NLS **gRNA**: T7-sgRNA	Target and investigate the role in azole resistance of *CYP51A* gene	Umeyama et al. (2018); Ballard et al. (2019)
***Cryptococcus neoformans*:** Cryptococcosis in lungs leading to meningoencephalitis if spread to the brain			
*ADE2*: achieved 70% gene disruption rate	**Cas9**: TEF1p:Cas9-SV40NLS:TEF1t **gRNA**: pACT1:HH-gRNA-HDV:TRPt	Biolistic transformation to introduce CRISPR/Cas9 components and the first proof of principle study	Arras et al. (2016)
*ADE2*, *Tsp2-1*: over 80% gene deletion rate	**Cas9**: ACT1P:V40NLS-Cas9:NLS-bGHpAt **gRNA**: pCnU6:GN19-gRNA:6Ts	“suicide” system for the elimination of CRISPR components and developed a system for gene complementation with reduced off-target effects	Wang et al. (2016)
*ADE2*: Upto 90% gene disruption rate	**Cas9**: GPD1p:Cas9:GPD1t **gRNA**: pCnU6:sgRNA:6-Tt	TRACE (transient CRISPR–Cas9 coupled with electroporation) to introduce CRISPR/Cas9 components	Fan and Lin (2018)
***Mucor circinelloides*:** Mucormycosis immunocompromised individuals			
*CARB*, *HMRB*: White colonies due to the disruption of β-carotene production with 100% targeting efficiency of NHEJ and HR	**Cas9**: Alt-R:CRISPR-Cas9:tracrRNA gRNA: Alt-R:CRISPR-crRNA	Introduced a plasmid-free CRISPR-Cas9 approach to obtain stable mutants	Nagy et al. (2017)
***Rhizopus delemar*:** Mucormycosis immunocompromised individuals			
*PYRF*: 36–59% gene disruption efficiency	**Cas9:** pmCas9:tRNA-gRNA **gRNA:** pmCas9:tRNA-gRNA	Point mutation introduced to investigate its pathogenesis mechanisms	Bruni et al. (2019)

### Developing diagnostics for fungal diseases and the therapeutic potential of the CRISPR/Cas9 system

As the socioeconomic burden of genetic diseases increases, numerous attempts were taken to test the CRISPR/Cas9 system as a tool for disease diagnostics, correct genetic abnormalities, discover target cells of drugs, and assess its feasibility and future possibilities in clinical applications (Hu et al. 2014; Lin et al. 2014; Zhen et al. 2014; Liu et al. 2015a, b; Kennedy et al. 2015; Park et al. 2015; Yu et al. 2015; Long et al. 2016; Nelson et al. 2016). With the popularization of precision or personalized medicine in medical practice, CRISPR/Cas9 took the center stage as a probable tool for diagnostic and therapeutic interventions (Karimian et al. 2019; Semiz and Aka 2019).

CRISPR-based diagnostic platforms provide rapid, sensitive, specific and reliable diagnostics for non-infectious and infectious diseases caused by bacteria, fungi and viruses (Bhattacharyya et al. 2018; Jolany vangah et al. 2020). CRISPR-based tools rely mainly on identifying sequences associated with a disease or a pathogen. The CRISPR system has been used as a diagnostic tool for pathogenic bacteria (*Staphylococcus aureus*, *Enterococcus faecium*, *Mycobacterium tuberculosis*, enterohemorrhagic *Escherichia coli*) (Delannoy et al. 2012; Ai et al. 2019; Quan et al. 2019) and viruses (human papillomavirus, human immunodeficiency virus, flaviviruses, COVID-19) (Myhrvold et al. 2018; Zhang et al. 2020a, b, c, d). To date, the CRISPR-based diagnostic tools are commercially available to diagnose genetic, bacterial and viral diseases (Jolany vangah et al. 2020), and very few are available for fungi. For example, the rapid CRISPR/dCas9-based detection kits are available to diagnose *Candida albicans* (The International Genetically Engineered Machine Competition: UiOslo_Norway, accessed at: http://2018.igem.org/Team:UiOslo_Norway; accessed on August 2021). Furthermore, a new forecasting system was introduced as a promising, portable platform of molecular tools, including the CRISPR system to detect pathogenic fungal species (Arastehfar et al. 2019). Even though not much research has been conducted on the diagnosis of fungal diseases, this technique has the potential to develop diagnostic tools for fungal diseases in the foreseeable future.

The CRISPR/Cas9 has been tested for its applicability in in vivo gene therapy in diseased cell lines and diseased mouse models, involving the direct transfer of nucleases or donor DNA templates into diseased cells and tissues (Hu et al. 2014; Lin et al. 2014; Zhen et al. 2014; Liu et al. 2015a, b; Kennedy et al. 2015; Park et al. 2015; Yu et al. 2015; Long et al. 2016; Nelson et al. 2016). The CRISPR has been successfully used in gene therapies in diseased mouse models to inactivate or correct deleterious mutations responsible for diseases with no effective treatment plans, such as Duchenne muscular dystrophy (DMD) (Ousterout et al. 2015) and has been shown to correct 2–100% in DMD mouse models with 15–20% therapeutic benefits (Long et al. 2016; Nelson et al. 2016). Further, CRISPR/Cas9 technology facilitates the insertion of corrective or protective mutations, such as in Haemophilia A (Park et al. 2015), Sickle-cell anaemia and β-thalassemia (Huang et al. 2015; Song et al. 2015). CRISPR/Cas9 has been efficiently used in different cell lines to disrupt viral DNA by inactivating the viral gene expression and replication of human immunodeficiency virus (Hu et al. 2014) and other viruses, such as hepatitis B (Lin et al. 2014; Liu et al. 2015a, b; Kennedy et al. 2015), and human papillomavirus (Zhen et al. 2014; Yu et al. 2015). Due to the rapid development, many studies and clinical trials adopt CRISPR/Cas9 system as a therapeutic strategy. In addition to these genetic diseases and disorders, CRISPR-based gene alteration therapeutic studies have been assessed against fungal diseases, such as deadly Cryptococcosis caused by *Cryptococcus neoformans* and *C. gattii* (Arras et al. 2016; Wang et al. 2016; Fan and Lin 2018). Similar studies have been conducted to replace virulence genes in human pathogenic fungal species, such as the *cyp51A* gene in azole-resistant clinical *Aspergillus fumigatus* isolates (Umeyama et al. 2018), *carB* and *hmgR2* genes of *Mucor circinelloides* (Nagy et al. 2017), and *pyrf* gene in clinical isolates of *Rhizopus delemar* (Bruni et al. 2019). Furthermore, CRISPR/Cas9 has been used in cancer and stem-cell research as a highly-specific and adaptable tool to correct mutations in cancer cell lines (Kim et al. 2017). In addition to being a therapeutic tool, the CRISPR/Cas9 has been assessed for developing anticancer drugs. For example, CRISPR-based studies conducted on *Candida* species facilitate the development of new antifungal drugs (Min et al. 2016, 2018; Lombardi et al. 2017; Shapiro et al. 2018; Halder et al. 2019) and have been used to discover drugs against *Cryptococcus* species (Nargesi et al. 2021). Even though, most CRISPR-based studies are not directly related to fungal diseases, the development achieved can be applied to treat fungal diseases in the future.

Major difficulties in treating fungal diseases are drug resistance and the biofilm development encoded by adhesion genes (Wyss Institute for Biologically Inspired Engineering at Harvard 2017). Using the CRISPR/Cas9 technology, Shapiro et al. (2018) suggested adhesion genes as targets for therapy against Candidiasis infections of *Candida albicans*. With the exception of a few studies on fungal diseases, almost all of the CRISPR-based diagnostic and therapeutic studies have been conducted on genetic, viral and bacterial infections. Together, these studies demonstrate a great promise to use the CRISPR/Cas9 to facilitate drug target discoveries, disease therapeutics, development of drugs and pathogen diagnostics for human fungal pathogens in the future.

### Developing fungal cell factories for the production of secondary metabolites of pharmaceutical importance using CRISPR/Cas9 system

Microbial secondary metabolites are widely exploited for their use as antibiotics, anticancer drugs, cholesterol-lowering agents, immunosuppressive drugs and other medicinals (Newman and Cragg 2016; Nielsen and Nielsen 2017). Due to the limitations of natural metabolite production by microbes, metabolic engineering uses the CRISPR/Cas9-based transcriptional activation in many fungal models as a tool to overexpress genes involved in bioactive secondary metabolite biosynthesis (Leonard et al. 2007; Gauthier et al. 2012; Weber et al. 2017; Sanson et al. 2018; Wang and Coleman 2019; Roux et al. 2020; Wei et al. 2020; Jiang et al. 2021). These microbial cell factories produce a repertoire of metabolites important for clinical therapeutics (Jiang et al. 2021). Many studies were conducted on filamentous fungi, such as *Aspergillus oryzae* and *Trichoderma reesei* using the CRISPR/Cas9 and the CRISPR-activation (CRISPRa) techniques to develop bioactive products and their derivatives for biopharmaceuticals (Roux et al. 2020).

Trypacidin is an antimicrobial compound of medical importance produced naturally by *Aspergillus fumigatus*, which is toxic to human lung cells (Gauthier et al. 2012). Weber et al. (2017) used the CRISPR/Cas9 tool to reconstitute Trypacidin production effectively. Pneumocandin B_0_ produced by *Glarea lozoyensis* is essential for synthesising Caspofungin, an antifungal drug approved by the USFDA against aspergillosis and certain *Candida* infections (Leonard et al. 2007). Due to its importance in pharmaceuticals, studies were conducted to enhance Pneumocandin B_0_ accumulation using the CRISPR/Cas9 tool (Wei et al. 2020). In addition, CRISPRa has been used to increase the transcriptional regulation of biosynthetic pathways of secondary metabolites of pharmaceutical importance, such as microperfuranones of *Aspergillus nidulans* (Sanson et al. 2018). In some fungi, the CRISPR/Cas9 has been used to identify genes related to the synthesis of secondary metabolites, such as in *Talaromyces atroroseus* (Nielsen et al. 2017). Similarly, it brings unlimited opportunities to accelerate the production of secondary metabolites, efficiently.

Penicillin, one of the first discovered antibiotics that belong to the class of beta (β) lactam antibiotics, is derived from highly complex nonribosomal peptide synthetase (NRPS) enzymes, which require multiple synthesis steps (Fleming 1929). The wild strains of *Penicillium chrysogenum* produce a negligible amount of Penicillin (Sawant and Vamkudoth 2022). Since their discovery by Alexander Fleming, *P. chrysogenum* strains have been improved using classical approaches, such as random mutagenesis, classical genetic engineering and fermentation, resulting in marginal increments in industrial penicillin production. For example, application of gene knockout has resulted in a low success rate in *P. chrysogenum* (Snoek et al. 2009; Hoff et al. 2010; Fierro et al. 2022). However, Pohl et al. (2016) established a marker-free CRISPR-Cas9 technique on *P. chrysogenum* by successfully editing several secondary metabolite genes. This technique can be used to edit and regulate several factors that limit penicillin production, including the number of genes involved, regulatory proteins, the supply of precursors, and co-factors. For example, Mózsik et al. (2019, 2021) demonstrated that replacing the natural promoter of the penicillin gene cluster with engineered and adjustable promoters faciliates higher yields of penicillin based on the type of promoter used in engineered strains. Similarly, terminators, transcription factors, and regulatory and DNA-binding domains of transcriptional regulators present in the gene clusters participating in the penicillin biosynthesis pathway in *P. chrysogenum* can be optimized using CRISPR-Cas9 technologies to achieve better yields of penicillin. These new synthetic transcription units produced from CRISPR-Cas9 technologies may be important for incorporating and assembling new fungal cell factories. Therefore, the CRISPR-Cas9 technique can facilitate the engineering of the biosynthetic gene cluster of penicillin in *P. chrysogenum* to improve the production yield of penicillin.

### Conclusions and future perspectives

The CRISPR/Cas9 systems have been successfully developed for many fungal species, paving the way for progress in genetics and molecular biology of medically important fungi. More than the engineered nucleases, the CRISPR/Cas9 seems simple in design and comparatively faster. Genetic changes performed with the CRISPR/Cas9 system, such as deletions, mutations, barcoding and tagging, are achieved throughout the genome and in specific sites causing either a single gene mutation or multi-gene expression regulation. The multiplexing capabilities of the CRISPR system are important to develop fungal cell factories that produce medically important metabolites.

The flexibility of this tool has shown potential in understanding biology, pathogenesis and virulence factors in medically important fungi in research settings. Kwon et al. (2019) evaluated the possibilities of using this method in the application stage for different fungal species, while McCarthy (2020) tested for medically important fungi. Accurate and fast identification is paramount for preventing and treating fungal diseases. Although not widely used, diagnostic methods like genome imaging using CRISPR/Cas9 technology are important for medical mycology to detect medically important fungi. Pathogen diagnosis using CRISPR/Cas9 has been established for antibiotic-resistant bacterial and viral pathogens, such as *Staphylococcus* *aureus*, Zika, dengue and most recently SARS-CoV-2 viruses. Speed and accuracy are assured in these CRISPR/Cas9 based modified methods of SHERLOCKv2 and CRISPR-Chip, which only takes up to 15 min to produce results for the diagnosis. Timely diagnosis is extremely important for immunocompromised patients as early therapeutic approaches could lower the mortality rate (Legrand et al. 2016). Even though, it has not yet been applied for fungal diagnostics, the development of CRISPR-Chips for diagnosing fungi is important in the future. These highly specific and sensitive methods could deliver results to smartphones even in low-infrastructure settings (Lau et al. 2020; Lackner et al. 2021). In addition to these diagnostic purposes, studying other application potentials of CRISPR/Cas9 technology, such as drug discovery, antifungal resistance, and host-fungal interactions, could advance the field of medical mycology in future.

## Bioeconomy of mushrooms

### Mushroom trade development

Mushrooms have been used as food and medicine for thousands of years. The earliest reports of mushroom consumption come from Spain (18,700 years ago), China (5000 to 6000 years ago), and Egypt (4600 years ago) (Chang 2006; Power et al. 2015; Straus et al. 2015). Despite the long history of human use and consumption, it is only in recent decades that mushrooms have been truly embraced, as reflected in the ever-increasing global bioeconomy of mushrooms.

**Fig. 10:**
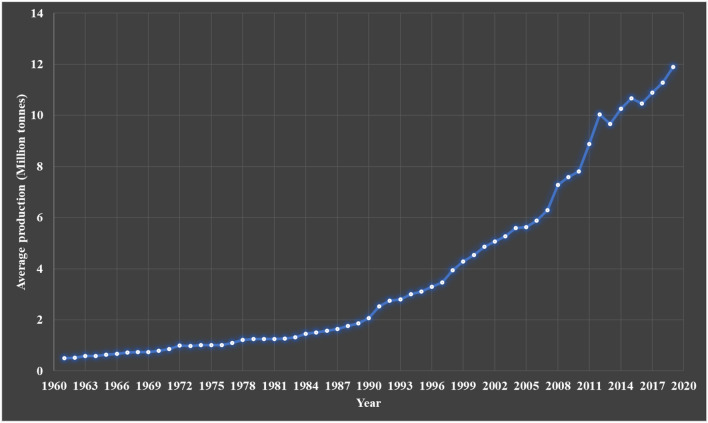
Global production of mushrooms and truffles from 1961 to 2019 (Food and Agriculture Organization Statistical 2019a). Aggregate global data may include official, semi-official, estimated, or calculated data

**Fig. 11:**
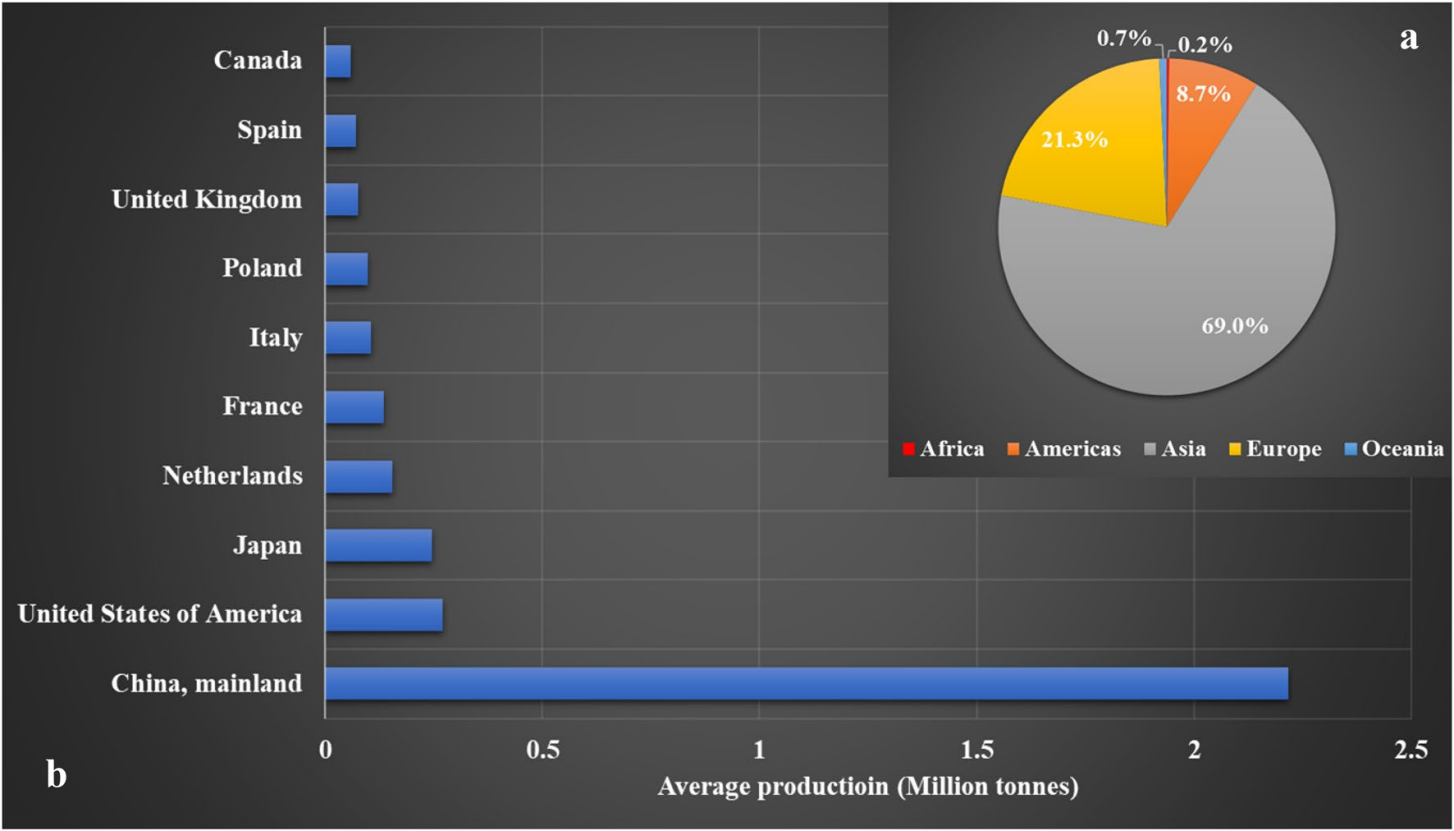
Production share (**A**) and top 10 producers (**B**) of mushrooms and truffles. Average production was calculated by the aggregate data of mushroom and truffle trade and production from 1961 to 2019. Aggregate global data may include official, semi-official, estimated or calculated data (Food and Agriculture Organization Statistical 2019a)

The current mushroom trade and consumption numbers have reached unprecedented levels. Royse (2014) reported that over a 15-year stretch (1997–2012), per capita global consumption of mushrooms has increased fourfold, from 1 to 4 kg/year. The global mushroom trade has grown continually since the 1960s, spurred by the development of mushroom cultivation technology to meet increasing consumer demands. At present, production figures in the mushroom market have increased 21-fold over the last 58 years (Figs. 10, 11). Rising consumer awareness about the myriad health and wellness benefits of mushrooms, greater knowledge of different varieties, and their innovative use as a meat substitutes are factors all driving the global market trends (Raut 2019). From 1961 to 2019, the overall pattern characterizing the global mushroom trade has shifted in two main ways: (1) In the past producers tended to only grow a single species, however, this trend has changed to producers growing a range of species, such as *Lentinula edodes*, *Pleurotus ostreatus*, and *Flammulina velutipes*, thereby diversifying their trading models; and (2) industrial mushroom production hubs have relocated from Europe and North America to Asia. In the 1970s, mushroom production was mainly distributed in the Netherlands, Germany, France, UK, Italy, and America. However, after the widespread dissemination of mushroom cultivation technology at the 9th International Edible Mushroom Congress in 1974, Asian countries like China, Japan, and South Korea became industry powerhouses for edible mushroom production (Zhang et al. 2015).

### Types of trade mushrooms

**Fig. 12:**
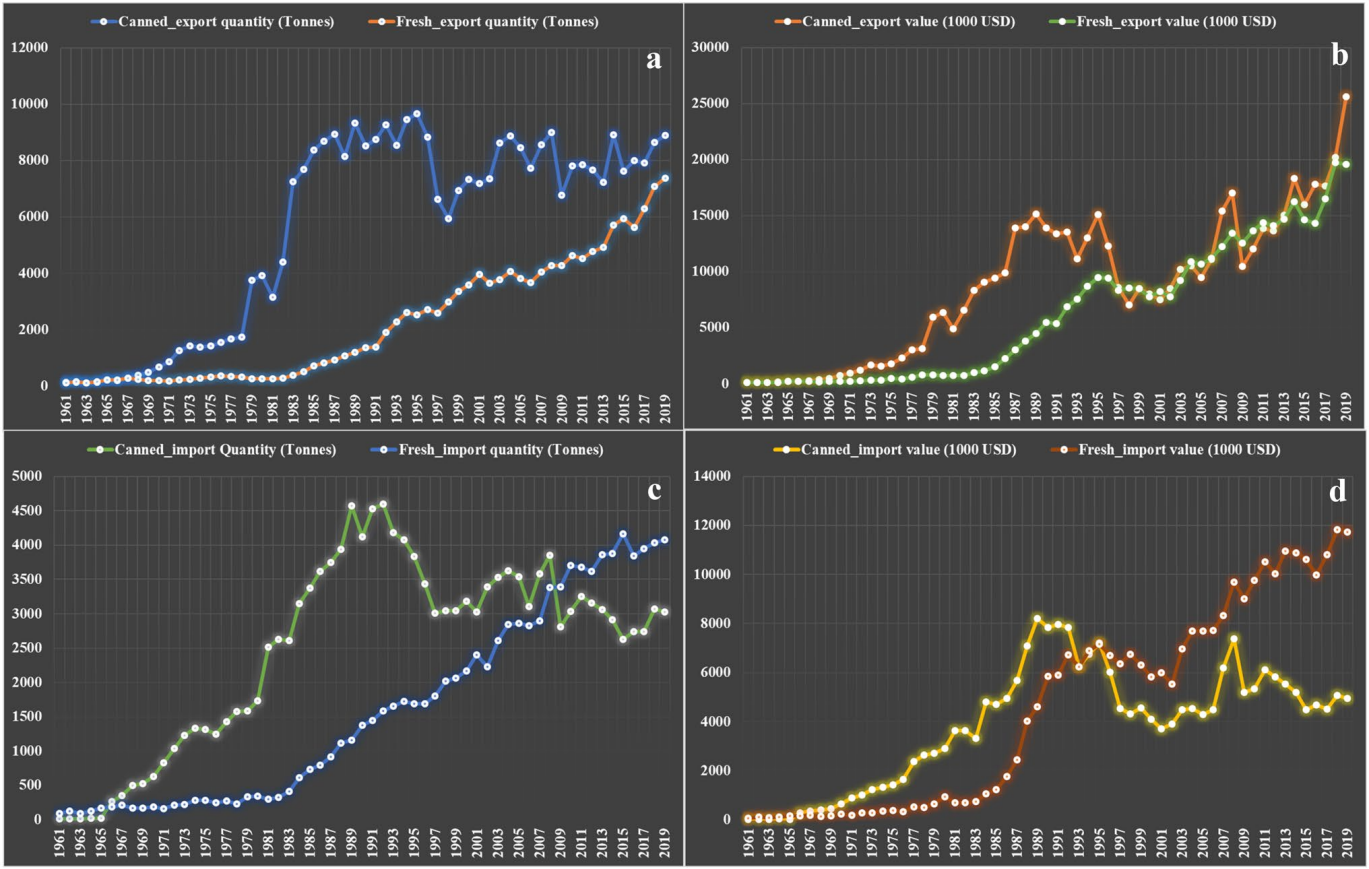
Annual change of quantity and value of mushroom products. **A** export quantity of canned and fresh mushrooms, **B** Export value of canned and fresh mushrooms, **C** Import quantity of canned and fresh mushrooms, **D** Import value of canned and fresh mushrooms. Fresh mushroom products comprise both mushrooms and truffles in the FAO database (Food and Agriculture Organization Statistical 2019b)

Generally, mushrooms (both wild harvested and cultivated) are traded as either fresh or processed mushroom products (Wakchaure 2011). Fresh mushrooms usually receive minimal processing (e.g., cleaning, packing) and are sold directly in markets or supermarkets. Processed mushroom products typically undergo drying, canning, pickling, and freezing. When looking at global mushroom products, the market share of fresh mushrooms is lower than processed mushroom products (Fig. 12), while the total export amount and value of fresh mushroom products has increased year after year (Fig. 12). Canned mushroom products dominate the market, with China and the Netherlands producing the highest amount of canned mushroom products (FAOSTAT, 2019a; b accessed on 27 April 2021).

**Fig. 13:**
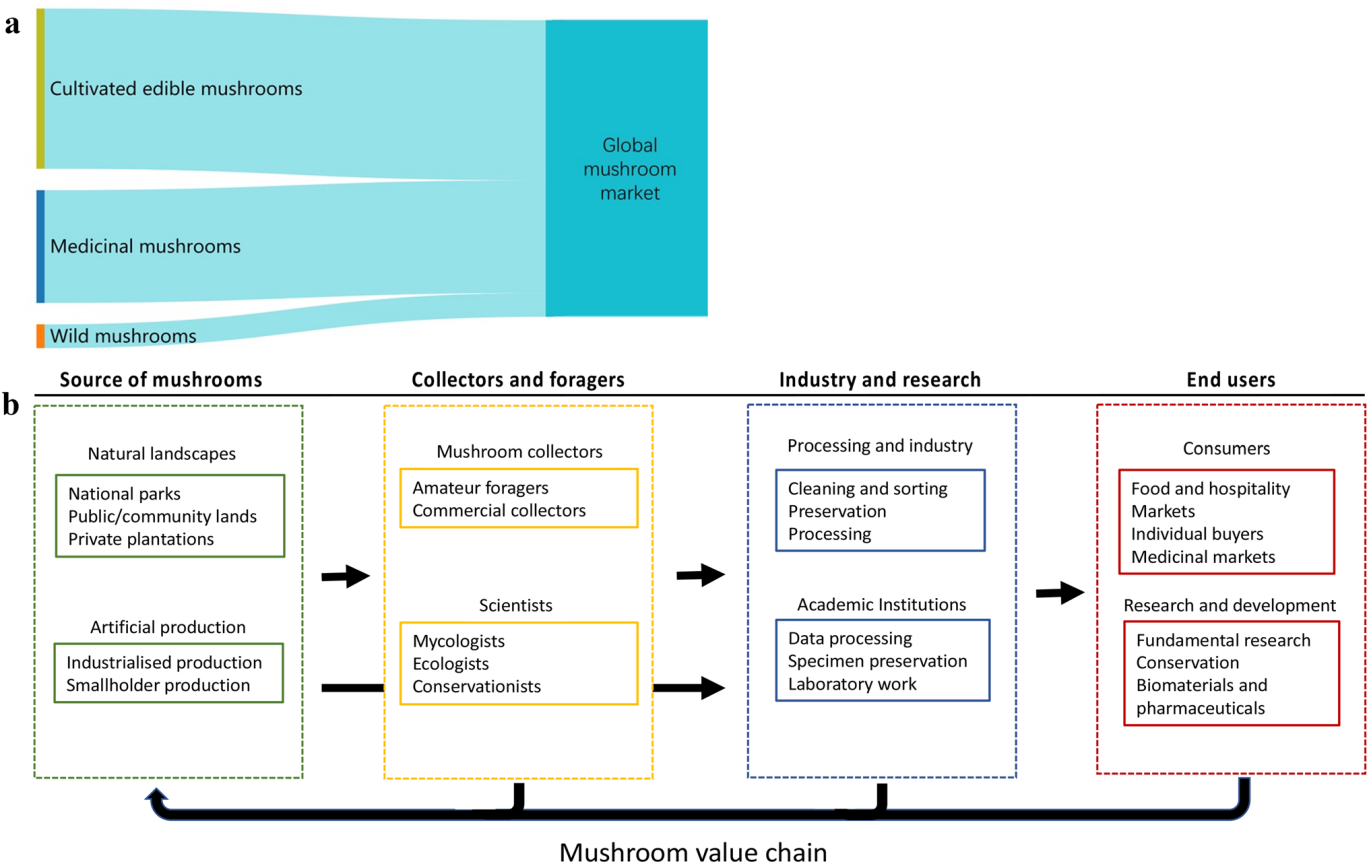
Relative contribution of the three major sectors (cultivated edible mushrooms, medicinal mushrooms, and wild harvested mushrooms) to the global mushroom market (**A**), generated using data from Royse et al. (2017); and the mushroom value chain highlighting the key sectors within the mushroom industry, and the feedback loops between these sectors (**B**)

In addition, trade mushrooms include cultivated edible mushrooms, medicinal mushrooms, and wild edible mushrooms (Chang 2006). The mushroom industry was estimated to be worth approximately 63 billion USD in 2013, of which cultivated mushrooms account for approximately 34 billion (54%), medicinal mushrooms comprise 24 billion (38%), and wild mushrooms are worth 5 billion (8%) of the overall mushroom industry (Fig. 13A) (Royse et al. 2017).

### Value chain and economic sectors of the mushroom trade

Mushrooms are primarily used as foods and medicines; accordingly, these commodities dominate the mushroom trade. These products are derived from either wild harvesting of mushrooms or from artificially cultivated mushrooms and can be divided into three main categories: wild harvested, medicinal, and cultivated edible mushrooms (Fig. 13A). Products within these sectors include fresh, functional, and designer foods; dietary supplements; drugs and mycopharmaceuticals; and nutraceuticals. The value chain representing the mushroom trade and encompassing the three major economic sectors within this trade is shown in Fig. 13B. Mushrooms are sourced from either the wild or through industrialised cultivation processes and used for the purpose of economic development, first by intermediary producers and collectors, and then later by industries related to the processing, marketing and sales of mushroom products. The role of scientists and research institutions also needs to be recognised within the value chain. Scientists drive discovery and domestication of new mushroom species for introduction to the market (Thawthong et al. 2014) as well as providing feedback on the quality and efficiency of existing production lines. Furthermore, scientists monitor and formulate baseline data used in developing management strategies for the conservation of natural habitats. These habitats function as sources of new species and as habitats for economically valuable, wild harvested mushrooms.

### Wild mushroom trade

Trade and consumption of wild harvested mushrooms benefit a range of economic sectors. Mushrooms collected by rural communities in isolated parts of Asia and South America are sold in large developed cities thousands of kilometres away. The urban demand for wild mushrooms drives an extensive value chain, providing income to numerous actors involved. As reported by de Frutos (2020), in 2017 the volume of trade in wild mushrooms was greatest in the European Union, followed by East Asia, and the Pacific region, whereas South Asia and sub-Saharan Africa recorded the lowest trading volumes (Table 2). However, when assessing the average increase in trade from 2002 to 2017, South Asia showed by far the greatest increase (12.74%), indicating that areas with low wild mushroom utilization and trade are beginning to value these mushrooms and see them as an important commodity (Table 2). Conversely, some areas, such as Latin America and the Caribbean, showed almost no change in the trade of wild mushrooms over this period (Table 2), suggesting either market saturation or a lack of cultural predisposition towards the use of wild mushrooms.

**Table 2:** Trade of wild edible mushrooms by geographical region (in tons) Adapted from de Frutos (2020)

Geographical region	2002	2017	Average annual trade increase (%)
South Asia	1.8	3532.8	12,74
European Union	181 461.1	753 029.3	21
Other European Countries and Central Asia	16 582.1	38 699.7	9
Latin America and Caribbean	3282.2	4465.9	2
North America	14 139.6	26 512.6	6
East Asia and Pacific	156 189.6	390 156.1	10
Arab World	1953.6	9363.1	25
Sub-Saharan Africa	927.2	3481.3	18

High-value wild mushroom species vary across different regions (Table 3). Harvesting commercial mushroom species from the wild, such as matsutake (*Tricholoma* sp.), boletes (*Boletus* sp.), truffles (*Tuber* sp.), morels (*Morchella* sp.), and various *Lactarius* species (e.g. *L. deliciosus*) is a lucrative practice in many countries and generates essential income for collectors and their families (Boa 2004; de-Román and Boa 2006; Yeh 2000). Global trends differ in which species of mushrooms are most sought after, which subsequently influences the trade and bioeconomy of mushrooms at the regional level. In Asia, the most sought after species are *Astraeus hygrometricus, Boletus edulis, Morchella conica, Ophiocordyceps sinensis, Phlebopus portentosus, Pleurotus giganteus, Termitomyces eurhizus, Thelephora ganbajun, Tricholoma matsuake,* and *Tuber indicum* (Mortimer et al. 2012). *Boletus edulis, Cantharellus cibarius, Lactarius delicosus, Morchella esculenta, Imleria badia, Agaricus campestris,* and *Cantharellus curnucopioides* are top sellers in the commercial markets of European countries, of which *Boletus edulis* and *Cantharellus cibarius* are the market leaders (Peintner et al. 2013). The most important commercially harvested wild mushrooms in the Pacific Northwest of the United States (Table 3) include *Tricholoma magnivelare, Morchella* sp*., C. formosus, C. cibarius, C. subalbidus, Hydnum repandum, Boletus edulis, Tuber gibbosum,* and *Leucangium carthusianum*.

**Table 3:** Main mushroom species that are eaten and traded in different regions

Scientific names	Price per kg (USD)	Region	References
*Astraeus hygrometricus*	3–5	Asia	Dell et al. (2005), Butkrachang et al. 9^2007^)
*Boletus edulis*	11	Europe, Asia, North America	Pilz and Molina (2002)
*Cantharellus formosus*	6	North America	Pilz and Molina (2002)
*Cantharellus subalbidus*	5	North America	Pilz and Molina (2002)
*Hydnum repandum, Hydnum umbilicatum*	7	North America	Pilz and Molina (2002)
*Leucangium carthusianum*	100	North America	Pilz and Molina (2002)
*Morchella conica*		Asia	Mortimer et al. (2012)
*Morchella* sp.	11	North America	Pilz and Molina (2002)
*Ophiocordyceps sinensis*		Asia	Mortimer et al. (2012)
*Phlebopus portentosus*		Asia	Mortimer et al. (2012)
*Termitomyces eurhizus*		Asia	Mortimer et al. (2012)
*Thelephora ganbajun*	120–200	Asia	He et al. (2011)
*Tricholoma magnivelare*	33	North America	Pilz and Molina (2002)
*Tricholoma matsuake*	27–560	Asia, North America, Europe	Wang et al. (1997)
*Tuber gibbosum*	50	North America	Pilz and Molina (2002)
*Tuber indicum*		Asia	Mortimer et al. (2012)

### Cultivated mushroom trade

Evidence suggests that mushrooms were first cultivated in Asia, with ancient texts indicating that *Auricularia* spp. were grown around 600 AD in China, followed by the cultivation of *Lentinula* spp. in China around 1000 AD (Zhang et al. 2015). The Chinese maintain the tradition of mushroom cultivation to this day and are currently the global leaders in the production and consumption of cultivated mushrooms (Fig. 11).

Over 30 billion kg of mushrooms were produced in China in 2013, accounting for about 87% of total worldwide production. In comparison, the rest of Asia produced about 1.3 billion kg, while the European Union, the Americas, and other countries collectively produced about 3.1 billion kg (Royse et al. 2017).

Currently, there are more than 100 species of edible macrofungi that can be artificially cultivated, of which about 60 species are cultivated commercially (Chang and Miles, 2004). Most of these are saprobic mushrooms (Chang 2008; Stamets 2000). The edible macrofungi most commonly cultivated as food or medicine are *Agaricus bisporus*, *Pleurotus* sp., *Auricularia auricula*, *Coprinus comatus, Hericium erinaceus*, *Hypsizygus ulmarius Ganoderma lingzhi*, *Grifola frondosa*, *Flammulina filiformis*, *Lentinula edodes*, *Pholiota microspora Tremella fuciformis* and *Volvariella volvacea*. Out of these, *Agaricus bisporus*, *Lentinula edodes* and *Pleurotus* sp. are produced in the greatest volumes. Currently, 90% of global mushroom production originates from *Lentinula, Agaricus, Pleurotus, Auricularia, Flammulina,* and *Volvariella* (Raut 2019). *Lentinula* is the most widely grown mushroom, accounting for over 2 million tons in global production; *Pleurotus* sp. are the second-most widely grown, with an annual production volume of approximately 0.4 million tons. *Auricularia* sp. make up the third-largest production volume of mushrooms, with 73,840 tons grown annually, followed by *Agaricus bisporus* (11,076 tons), *Flammulina* (45,120 tons), and *Volvariella* (20,410 tons). *Lentinula*, *Pleurotus* and *Agaricus* are cultivated worldwide, whereas *Auricularia*, *Flammulina*, and *Volvariella* are grown almost exclusively in Asia (Royse 2014, 2017).

### Emerging economic sectors

Many emergent industries are beginning to utilize mushrooms for non-food-based products. It is likely that in the future, these industries will contribute significantly towards the mushroom bioeconomy (Ghazvinian et al. 2019). Mycelium-based biomaterials can be used to produce packaging (Abhijith et al. 2018; Holt et al. 2012) and furniture (Ecovative Design LLC 2021; MycoTech 2021; Mogu S.r.l. 2021; Krown Design 2021), with other applications in construction, revolutionizing the way these industries operate (Hyde et al. 2019). These products are produced using the mycelium or fruiting bodies of certain mushrooms, such as *Pleurotus* and *Ganoderma*. They are renewable, sustainable, and cost effective (Abhijith et al. 2018). Another innovative product is mycelium-based leather, offering a sustainable alternative to leather-based products used in fashion, automobile interiors, or the furniture industries (Attias et al. 2020).

### Challenges for the future growth of the industry

Currently, the global mushroom economy can be divided into wild and cultivated resources. Adopting a sustainable approach for the continued use of wild resources will be paramount for the industry to maintain its current growth trajectories. Improved forest management, sustainable harvesting techniques, and better post-harvest management of mushroom products are all required to ensure future growth. Similarly, sustainable practices can enhance cultivation techniques. Recycling of materials, adoption of renewable energy, and incorporation of new varieties of mushrooms into existing production lines are all important aspects worth consideration for the cultivation industry to continue its explosive growth.

Furthermore, the cultivation industry is also limited by current production technologies. Mushroom cultivation is predominantly confined to saprobic species, with some managed production of ectomycorrhizal species showing potential (e.g., inoculation of host trees with *Tuber* sp.). However, to meet current and future levels of demand as well as to alleviate the exploitation of natural forest systems, new techniques will be required to cultivate ectomycorrhizal species at industrial scales. Such advances will launch the mushroom bioeconomy into new heights.

## Mycelium-based technology

The synthesis of functional materials from biological resources has been receiving increasing attention in recent years (Cerimi et al. 2019). This is in accordance with the Green Economy transition, which represents growth and development that are consistent with environmental well-being (Söderholm 2020). There are growing concerns about the degradation of synthetic plastic, which initiated research focused on the use of materials from renewable resources such as fungal mycelium-based materials (Manan et al. 2021). Mycelium can be described as a network of interwoven, thread-like hyphae that constitute the vegetative part of fungi (Karana et al. 2018). Fungi decompose dead plant substrates by breaking down cellulose, hemicellulose, lignin, and other sugars into small molecules through the secretion of enzymes (Promputtha et al. 2010). The vegetative mycelium degrades and colonizes the organic substrate by using the products of degradation (Meyer et al. 2020a, b). During colonization of the substrate, fungi grow by extending its hyphae and the hyphae bind organic particles together to form a three-dimensional interwoven filamentous network (Karana et al. 2018). The mycelial network comprises individual hyphae ranging from about 2 to 20 μm in diameter (Fricker et al. 2017). Fungal mycelium grows on the surface and penetrates the substrate, while some grow out of the substrate and form a compact layer referred to as “fungal skin” (Grimm and Wösten 2018).

**Fig. 14:**
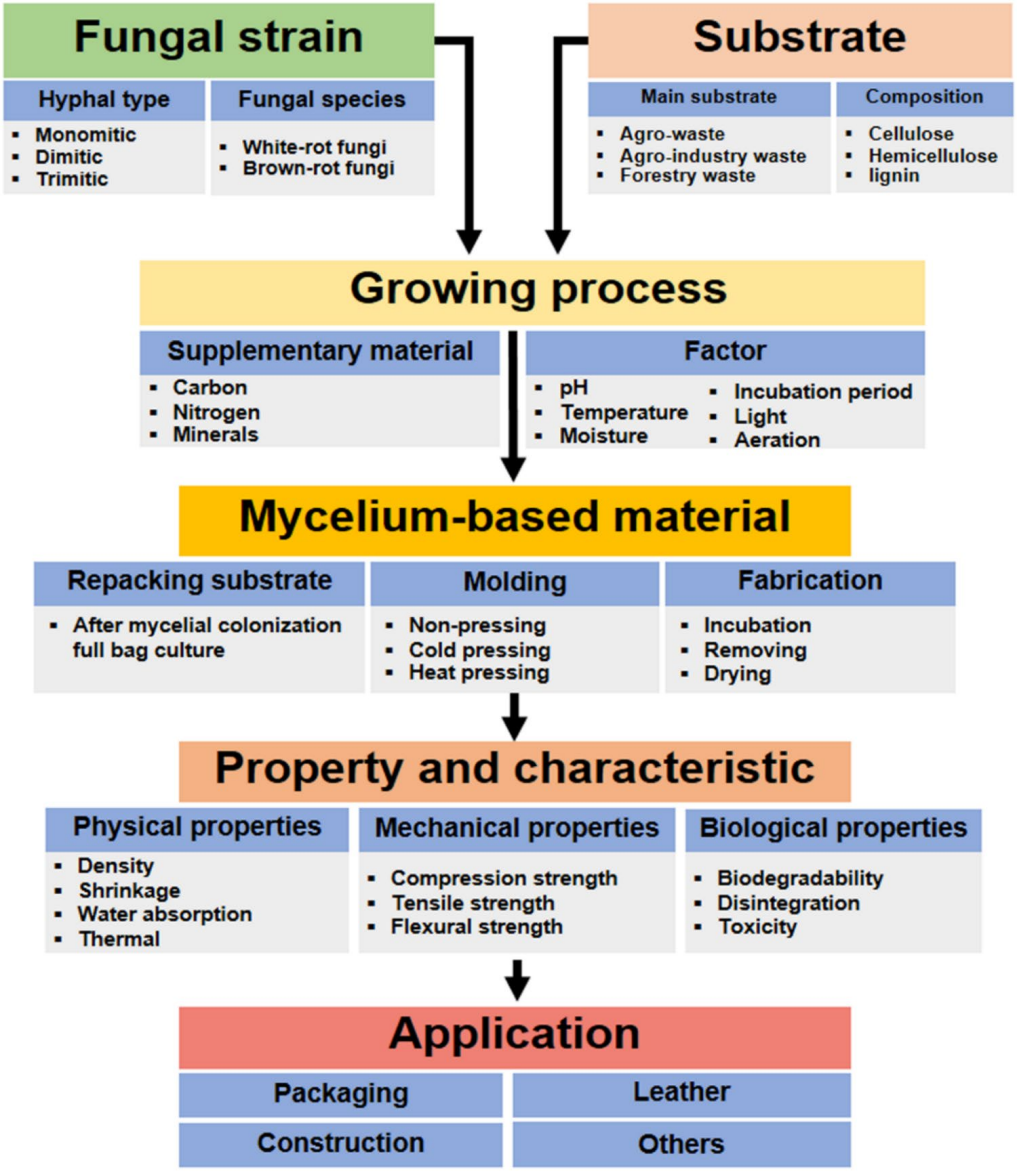
Schematic steps of the synthesis process of mycelium-based composite with key steps and possible variations in processes

Mycelium-based materials are grown either by allowing mycelium to interlock other substances to form a bulk material (mycelium-based composites) or by harvesting a liquid culture of mycelium (pure mycelium) (Holt et al. 2012; Haneef et al. 2017). Mycelium-based materials are produced by growing vegetative fungal hyphae on different organic substrates through solid-state fermentation (Pelletier et al. 2013; Islam et al. 2018). The properties of mycelium-based materials depend on the fungal strain, the type of substrate, the growth conditions, and the post-synthesis process (Appels et al. 2018, 2019). A schematic illustration presenting the different steps involved in the synthesis of mycelium-based material is shown in Fig. 14. Several Ascomycota and Basidiomycota genera have been used in mycelium-based technology (Attias et al. 2020, Table 4). White-rot and brown-rot fungi have mainly been utilized in the generation of mycelium-based materials due to their high colonization rate and ability to degrade a large amount of organic biomass (Cerimi et al. 2019). Different hyphal types can influence the properties of mycelium-based materials, for example, monomitic fungal species can provide less effective mechanical properties than dimitic and trimitic fungal species (Pegler 1996; Bayer and McIntyre 2012, 2015). For example, *Pleurotus ostreatus* and *Trametes versicolor* provide greater stiffness and strength in mycelium-based composites (Lelivelt 2015; Jones et al. 2020) whereas “*Ganoderma lucidum*” (probably wrongly named as this European species has not been safely recorded from China by specialists) can enhance the physical and mechanical properties of the composite (Liu et al. 2019). However, many publications have not identified the fungal species used in mycelium-based composite production (Parisi et al. 2016; Dahmen 2017; Jiang et al. 2017).

**Table 4:** Ascomycota and Basidiomycota species that have been used in mycelium-based technology

Phylum	Species name based on Index Fungorum
*Ascomycota*	*Morchella angusticeps*
	*Xylaria filiformis*
	*Xylaria hypoxylon*
	*Xylaria longipes*
	*Xylaria polymorpha*
*Basidiomycota*	*Cyclocybe aegerita* (*Agrocybe aegerita*)
	*Coprinus comatus*
	*Flammulina velutipes*
	*Fomes fomentarius*
	*Ganoderma lucidum*
	*Ganoderma oregonense*
	*Ganoderma tsugae*
	*Grifola frondosa*
	*Hericium erinaceus*
	*Hypholoma capnoides*
	*Hypholoma lateritium* (*Hypholoma sublateritium*)
	*Lentinula edodes*
	*Macrolepiota procera*
	*Pleurotus djamor*
	*Pleurotus eryngii*
	*Pleurotus ostreatus*
	*Laccocephalum mylittae* (*Polyporus mylittae*)
	*Pycnoporus cinnabarinus*
	*Trametes versicolor*

The ideal substrate for mycelium-based products should provide nutrients for mycelium growth, such as carbon, nitrogen, minerals, vitamins and water (Karana et al. 2018). The lignocellulosic forms of waste generated from routine agricultural, agro-industrial, and forestry practices are commonly used as the primary substrates for mycelium-based composite production (Pelletier et al. 2013; Jones et al. 2018). Some examples of substrate include wheat or rice straw and wood sawdust (Pelletier et al. 2013; Jones et al. 2018). Fungi split the polymeric plant substrates, which consist largely of lignin, cellulose and hemicellulose, into their monomeric components and synthesize new organic molecules (Karana et al. 2018). The composition of cellulose, hemicellulose, and lignin in lignocellulosic waste depends upon the species, tissue, and maturity of the plant (Grimm and Wösten 2018; Royse et al. 2017; Kumla et al. 2021; Moonmoon et al. 2011). The substrate composition can directly affect the ability of mushrooms to grow in a substrate, which can influence the technical and experiential qualities of the resulting material (Royse et al. 2017; Hoa and Wang 2015). Furthermore, the addition of various supplements in the substrates can support mycelia growth (Karana et al. 2018). Pure mycelium materials are harvested from liquid fermentation of fungi in static or machine-shaken containers (Karana et al. 2018). Filamentous fungi grown in static liquid culture form a mat of hyphae at the surface of the liquid and when dried the resulting material resembles leather, paper, or plastic (Karana et al. 2018). Many factors, such as light, humidity, temperature, and incubation period are important factors that can affect mycelium growth. Conditions of darkness are often preferred to prevent the formation of fruiting bodies and for rapid mycelium growth (Deacon 1980).

Different fabrication processes result in different functional aspects of mycelium-based composite (Karana et al. 2018). The residual water present in the mycelium-based composite is commonly removed by drying in an oven to produce lightweight and high-strength materials (Jiang et al. 2017). Moreover, the pressing involved in the fabrication process can result in a reduction of the porosity of the materials, thus increasing the material density and strength (Haneef et al. 2017; Appels et al. 2019; Liu et al. 2019). Mechanical, physical, and biological properties of mycelium-based composites are affected by the substrate type, the mycelia network and the pressing method (Pegler 1996; Appels et al. 2019). The high compressive strength and lightweight of mycelium-based composites enable them to be used as packaging and construction materials (Yang et al. 2021). Moreover, the low density, low thermal conductivity, and high porous characteristics of mycelium-based composites make them suitable for the production of alternative synthetic foam and wood fibers (Manan et al. 2021). Therefore, a better understanding of the beneficial properties of mycelium-based composites is crucial for their potential applications in a variety of fields. Another advantage of mycelium-based composites is that they are non-toxic and biodegradable in nature (Cerimi et al. 2019; Girometta et al. 2019; Yang et al. 2021). One of the unique features of mycelium-based materials is that they can be grown into any shape using a mold, which represents various possibilities in the textile, furniture or building materials industry (Cerimi et al. 2019). There is also a “Grow it yourself” kit developed by the Ecovative company, which is available to the public to produce their own composite material at home in any forms (Rognoli et al. 2015).

## Mycelium-based materials

### Mycelium-based packaging

The demand for packaging materials has increased significantly following global industrial growth (Söderholm 2020). Several petrochemical-based plastics mainly polystyrene, polyethylene, and polypropylene have been widely used in the production of packaging materials (Pavlineri et al. 2017). However, the production of plastic packaging materials contributes to the release of greenhouse gases, while plastic packaging is also known to be wasteful and leads to increased levels of environmental pollution (Verma et al. 2016). Therefore, several studies focusing on the performance of alternative materials have explored the development of new materials for packaging (Cerimi et al. 2019). Bioplastic production can be utilized to create an alternative to petroleum-based plastics, but the cost of bioplastics remains higher than petrochemical-derived plastics (Gill 2014). Mycelium-based materials can therefore represent a cheaper alternative for packaging applications for electronics, food, and fragile items (Abhijith et al. 2018; Ncube et al. 2020). The preference for these materials is based on their excellent renewable and biodegradable features (Fig. 14). Importantly, materials with non-toxic properties are preferred for use in the food industry (Hyde et al. 2019). The packaging production of mycelium-based materials focuses on the use of various agricultural residues and the mycelia of many fungal genera, namely *Agrocybe*, *Fomes*, *Ganoderma*, *Lentinula*, *Pleurotus*, *Polyporus*, and *Xylaria* (Abhijith et al. 2018; Cerimi et al. 2019). Mycelium-based packaging developed from *Pycnoporus cinnabarinus* has an orange-red color without the addition of any pigments (Cerimi et al. 2019; Manan et al. 2021). Mycelium-based packaging has been designed in various shapes by many companies depending on its intended use. The Ecovative Company, Shenzhen Tech., Beijing Zhongke Aobei Supersonic Wave Tech Res Inst., and Mycoworks Inc are examples of some of these companies. These companies have developed and patented several methods of manufacturing mycelium-based products as substitutes for conventional packaging materials (Cerimi et al. 2019; Manan et al. 2021).

### Mycelium-based leather

**Fig. 15:**
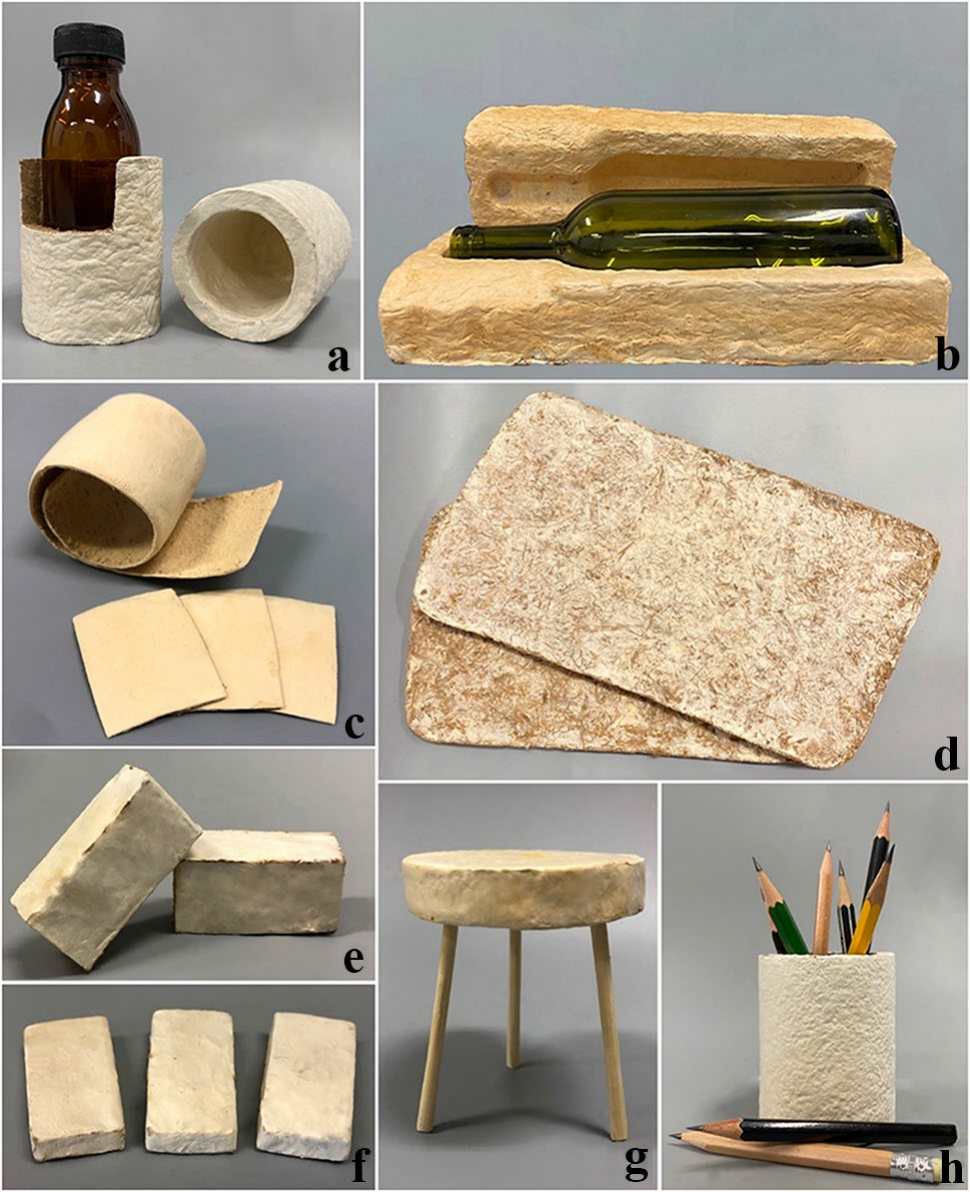
Applications of mycelium-based materials in different fields. **a, b** Packaging. **c** Leather. **d–f** Construction materials. **g, h** Others

Leather is a durable natural product that is produced from animal hides through processes involving physical and chemical treatments (tanning) (Kanagaraj et al. 2015). The demand for natural leather has increased because of its beauty, durability and softness (Kanagaraj et al. 2015). Therefore, the increased demand for livestock has major impacts on the environment due to an increased demand for land to raise animals for their skin (Dopelt et al. 2019). Therefore, several studies have focused on the production of alternative forms of leather (Cerimi et al. 2019). Artificial forms of leather that are synthesized from polyvinyl chloride and polyurethane have been promoted as substitutes for animal leather (Roh et al. 2013). However, these synthetic forms of leather also require the use of hazardous chemicals in the production processes (Roh et al. 2013). Furthermore, these materials also lack the characteristic of biodegradability and can increase environmental pollution as they are associated with the same limited end-of-life options as most plastics (Shah et al. 2008). Recently, other types of artificial leather have been produced as environmentally safe materials from plants and fungal biomass (Cerimi et al. 2019). Fungal biomass is advantageous over plants in terms of its high availability, stability and yield, lower amounts of residues, and the ease with which it can be harvested (Meyer et al. 2020a, b). Fungal biomass forms a mat of mycelia containing chitinous biopolymers that resemble leather (Karana et al. 2018). This biomass can be obtained from both liquid and solid fermentation processes (Javadian et al. 2020; Vandelook et al. 2021). After harvesting the fungal biomass, physical and chemical treatments are applied to improve the tissue density, tensile strength, and elastic properties (Vandelook et al. 2021). The production of mycelium-based leather focuses on polypore fungal species in the genera *Fomes*, *Ganoderma*, *Perenniporia*, *Pycnoporus* and *Trametes* (Fig. 15) (Gandia et al. 2020; Stewart et al. 2020; Manan et al. 2021). Since 2019, many prototypical products such as handbags, shoes, watch bands, and wallets are made from mycelium-based leather available under a variety of trade names. These include Mylea™ from Mycotech PTE. LTD., Reishi™ from Mycoworks, Mylo™ from Bolt threads, and VTT mycelium leather from the VTT research team (Ross et al. 2018; Sun et al. 2019; Bentangan et al. 2020; Smith et al. 2020). However, there are noticeable variations in the mechanical and physical properties between the various mycelium-based leather brands (Sun et al. 2019; Attias et al. 2020; Vandelook et al. 2021).

### Mycelium-based construction

Rapid urbanization has increased the demand for construction materials such as bricks, cement and insulation panels. The production of conventional construction materials translates to an even greater demand for large amount of energy which can have major impacts on the environment through harmful manufacturing methods (Madurwar et al. 2013; Jones et al. 2020). Therefore, the increased demand for the development of innovative construction materials has become of significant interest to researchers. Bio-based materials are considered a promising resource for buildings in the twenty-first century due to their sustainability and versatility (Karana et al. 2018). They can be produced from agricultural, agro-industry and forestry waste (Karana et al. 2018). Bio-based materials have low energy needs, low production costs, and are considered safe and eco-friendly (Abhijith et al. 2018; Sandak et al. 2019). Mycelium-based materials have become increasingly popular over the last decade in the construction, structure, and design industry (Almpani-Lekka et al. 2021; Sydor et al. 2022). Mycelium-based materials have the potential to be utilized in various applications such as alternative insulation materials for building and infrastructure development (Fig. 15). The production of mycelium-based construction materials has focused on producing alternative forms of bricks, blocks, boards, and panels made from various agricultural types of residues using the mycelia of *Fomes fomentarius*, *G. lingzhi*, *G*. *lucidum*, and *Pleurotus ostreatus* (Elsacker et al. 2021; Almpani–Lekka et al. 2021). Mycelium panels can serve as sound absorbers that can effectively reduce noise pollution (Pelletier et al. 2013). Ongpeng et al. (2020) found that mycelium-bricks possess better than average levels of compressive strength, flexural strength, and midpoint displacement, which can reduce the need for traditional construction materials. Many mycelium-based construction products are available from the Ecovative Company. However, there are problems associated with different mechanical applications, high water absorption properties, and a lack of standard methods for the production and testing of mycelium-based construction materials that need to be addressed in future studies (Javadian et al. 2020).

### Mycelium-based food

Fungi have been used as a source of food for humans throughout history (Hyde et al. 2019). The human consumption of fungi has predominantly involved mushrooms that originate from both natural habitats and cultivation processes (Chang 2006; Li et al. 2021; Perez-Moreno et al. 2021). The fruiting bodies of mushrooms can emerge in a variety of shapes, tastes and textures (Hyde et al. 2020b; Bhunjun et al. 2022). Some mushrooms have been described as being meat-like, such as beefsteak fungus (*Fistulina hepatica*) and chicken of the woods (*Laetiporus sulphureus*), as well as the seafood-like abalone fungus (*Pleurotus cystidiosus*) and the lobster mushroom (*Lactarius* and *Russula* parasitized by the ascomycete *Hypomyces lactifluorum*) (Rahi and Malik 2016). The dry matter of an edible mushroom is generally composed of protein (15–35%), carbohydrates (35–70%), essential fatty acids (less than 5%), as well as traces of vitamins and minerals (Barros et al. 2008; Valverde et al. 2015; Niego et al. 2021a). They are also known to have antioxidant, antimicrobial, anticancer, and immunomodulatory properties (De Silva et al. 2012; Kaewnarin et al. 2016, 2020; Valverde et al. 2015; Hyde et al. 2019). Traditionally, fungal mycelia have only been used as flavor and color modifiers in fermented foods such as blue cheese, red mold rice, soy sauce and tempeh (Hyde et al. 2019; Ahmad et al. 2020). However, meat-like products or mycoproteins have been produced from fungal mycelium via liquid fermentation process (Moore and Chiu 2001). Over the last three decades, *Fusarium venenatum*, has been used to produce mycoproteins on an industrial scale by Marlow Foods under the trade name Quorn™ (Finnigan 2011). Further, edible strains of filamentous fungi, such as *Aspergillus oryzae*, *Monascus purpureus*, *Paradendryphiella salina* and *Rhizopus oryzae* have also been used to produce mycoprotein via submerged fermentation or solid-state fermentation processes (Souza Filho et al. 2018; Reihani and Khosravi-Darani 2018; Landeta-Salgado et al. 2021). Several meat-like products have also been developed by companies such as Mycorena (*A. oryzae*), Sustainable Bioproducts (*F. oxysporum*) and MycoTechnology, using the basidiomycete *Lentinula edodes* (Meyer et al. 2020a).

Mycoprotein products are available in different forms, for example beef burgers, beef steaks, chicken nuggets, fish sticks, meatballs, sausages, among others (Joshi and Kumar 2015). Mycoprotein products from fungal mycelia are also available in the form of a bacon substitute by Atlast Food Co. under the trade name MyBacon™ (Meyer et al. 2020a). Mycoprotein is a widely accepted food and is approved for sale in all EU counties, as well as Australia, Canada, New Zealand, Norway, Switzerland, and the USA (Derbyshire 2020). It has recently been approved for sale in Thailand (Derbyshire 2020). Mycoprotein is considered a good source of high-quality proteins as it contains a higher percentage of essential amino acids (approximately 45%) than most other commonly consumed plant-based proteins (approximately 25%) (Finnigan et al. 2019). The consumption of mycoproteins can lead to the generation of slower and more sustained essential amino acids and branched chain amino acid levels when compared to milk (Finnigan et al. 2019; Dunlop et al. 2017). Moreover, the high bioavailability and amino acid composition of mycoproteins can stimulate a greater rate of muscle protein synthesis compared to milk protein in healthy young men (Dunlop et al. 2017). Subsequently, mycoproteins show great promise as a source of dietary protein that has the potential to support skeletal muscle protein metabolism (Coelho et al. 2021; Monteyne et al. 2020). Several studies have reported that mycoprotein consumption can change blood lipid levels by reducing plasma cholesterol and improving high-density lipoproteins (Turnbull et al. 1992; Ishikawa 1994; Ruxton and McMilan 2010; Coelho et al. 2021). Mycoprotein consumption is also associated with reduced insulin levels, sustained hyperinsulinaemia and hyperaminoacidaemia, improved immune function, reduced tumour-associated symptoms and extended survival rates in lung cancer patients (Turnbull and Ward 1995; Bottin et al. 2012; Fritz et al. 2015; Cherta-Murillo et al. 2020).

### Conclusion

Mycelium-based technology represents a unique and low-cost method to recycle agricultural waste into sustainable biomaterials. Mycelium-based materials offer a lightweight and environmentally friendly alternative to synthetic foams, but there are several challenges related to large-scale production. Despite rapid growth in our understanding of mycelium-based technology, there are crucial knowledge gaps. Therefore, future research is likely to focus on various fields including the standardization of the production processes. Only a small number of species have been used to develop mycelium-based materials and the study of a larger number of fungal species is likely to reveal multiple new applications in the furniture, agriculture, medicine, pharmacology, and cosmetics industries.

## Growing morels in China

True morels (*Morchella* spp., Pezizales, Ascomycota) are highly sought after and prized edible mushrooms, renowned for their great economic and scientific value (Du and Yang 2021; Loizides et al. 2022). Wild morels, mostly distributed in temperate regions of the Northern Hemisphere, have been reported to have a variety of ecological types, including saprotrophic, pyrophilic, and ectomycorrhizal (Pilz et al. 2004; Tan et al. 2019; Hussain and Sher 2021). They are distinguished by honeycomb-appearance, and typically fruit for only a few weeks each spring, with the exception of some autumn species (Matočec et al. 2014; Taşkin et al. 2015). Morels have strong health promoting abilities, because they are rich in nutrients, and their fruiting bodies or metabolites have anti-tumor, anti-inflammatory, antioxidant, neuroprotective and immunomodulatory effects (Dissanayake et al. 2021).

**Fig. 16:**
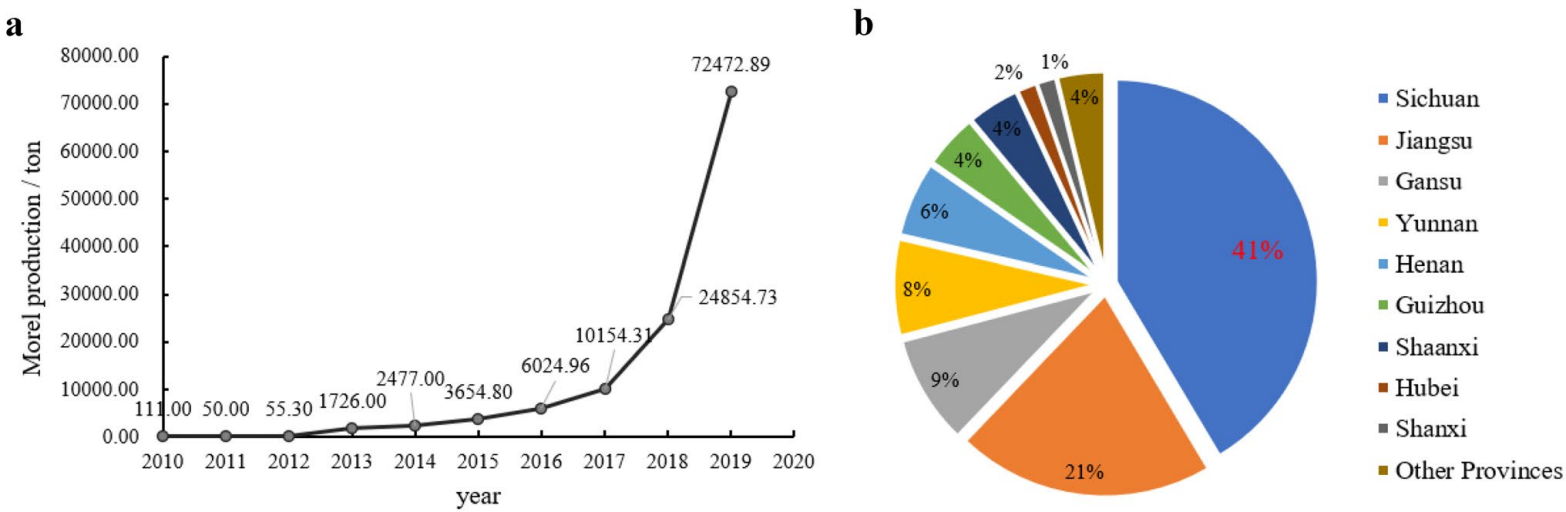
**a** Production of morels in China from 2010 to 2019, **b** Morel production in different provinces of China in 2019. Data sources: http://bigdata.cefa.org.cn/index.html (Accessed date: 21 Mar. 2022)

In light of morels subtle morphological features and high phenotypic plasticity, they are difficult to distinguish, and morphological species recognition of morels is questionable (Du and Yang 2021; Loizides et al. 2022). Since 2010, genealogical concordance phylogenetic species recognition (GCPSR) based on multi-locus sequences (ITS, *TEF*, *RPB*1 and *RPB*2) has become the most effective method for species identification within *Morchella* (O’Donnell et al. 2011; Du et al. 2012; Kuo et al. 2012). To date, over 80 species-level lineages of *Morchella* have been inferred by molecular phylogenetics, and they form three easily distinguishable evolutionary clades, i.e., the basal Brunnea clade, the Esculenta clade, and the Elata clade (includes semifree capped morels) (O’Donnell et al. 2011; Du et al. 2012; Kuo et al. 2012). Morel collection is of economic value, which provides an economic source for rural communities in Asia (Raut et al. 2019; Kakakhel 2020). In Nepal, 1.7 to 6.5 tons of dried morels are exported annually, mainly to Belgium, France, Germany, the Netherlands and Switzerland (Raut et al. 2019). Before 2011, wild morels dominated the Chinese market, despite their low output. Increasing market demand, short fruiting season, habitat fragmentation and excessive collection of wild resources have prompted the morel cultivation to become a hot spot in research at home and abroad (Du and Yang 2021; Zhao et al. 2021). With the explosive development of morel cultivation in China industry, from 2011 to 2019, the cultivated area rapidly expanded from 200 to 10,000 ha, and the output of fresh morels increased from ≤ 750 kg/ha to 15,000 kg/ha. The annual output in 2019 reached more than 70,000 tons (Fig. 16). The harvest of morels is mainly in Sichuan, Gansu, Yunnan, and Henan Provinces of China, with output of 30,049.70 tons (41%), 14,987.60 tons (21%), 6348.00 tons (9%), 5586.60 tons (8%) and 4360.60 tons (6%), respectively (Fig. 16). The international price of high-grade edible morels is about US $200–350/kg, the price of domestic artificially grown dried products is US $250–315/kg, and the price of wild dried products is even more expensive, about US $470-790/kg.

### History of artificial morel cultivation

Due to the highly desirable flavour and short fruiting season of morels, the artificial cultivation industry of morels has maintained rapid growth to meet the commercial demand. The first report on the outdoor cultivation of morels came from France (Roze et al. 1882), and since then, many specialists have attempted various ways to cultivate morels. Successful cultivation of morels was reported in apple compost in 1904 (Stott al. 2004). Later, Ower (1982) published a short description of how to artificially grow morels, and then patents (US Patents 4594809, 4757640) were issued for cultivation of morels that drew immediate attention in the world (Ower 1982; Ower et al. 1986, 1988). Their works were technology-centered (e.g., temperature, humidity and ventilation), and nutrient regimes to grow and prepare sclerotia for controlled germination into fruiting bodies. Moreover, Ower emphasized the important role of sclerotia in fruiting body development (Ower et al. 1986, 1988). Although these cultivation techniques were based on “*Morchella esculenta*” that it was later presumed to be *M. rufobrunnea* based on photographs (Kuo 2008), their patents claimed that these methods were suitable for all *Morchella* species. Unfortunately, it was difficult to apply these methods to the successful large-scale indoor cultivation of morels.

In China, the earliest record of morel cultivation can be traced back to the 1950s. During the period of 1950–2010, some researchers and farmers explored the process of artificial cultivation of morels, and there were intermittent reports on morel cultivation and the successful acquisition of fruiting bodies, but with no commercial morels in the market. Since 2010, the morel cultivation industry has developed rapidly due to the breeding of several black morel varieties with improved fruiting yield and stability, and the development and wide application of exogenous nutrient bags (Liu et al. 2017). With the development of these technologies, successful morel cultivation not only alleviated the shortage of wild morels in the market, but also greatly promoted the local economic development (Liu et al. 2017, 2018; Tan et al. 2019). At present, morel cultivation covers almost all areas in China.

### Morel species currently under cultivation

In China, at least eight phylogenetically distinct species have currently been cultivated artificially, i.e., *Morchella eximia*, *M. exuberans*, *M. importuna*, *M. oweri*, *M. sextelata*, *M. tomentosa*, *Mel*-13 and *Mel*-21 (Du and Yang 2021). Among them, the main cultivated species are *M. eximia*, *M. importuna* and *M. sextelata*, which account for more than 95% of the cultivated area, with high productivity and good stability (Zhao et al. 2021). *Morchella tomentosa* is only distributed in North America, and it is the basal species of the Elata Clade, corresponding to the phylogenetic species *Mel*-1 (Stefani et al. 2010; O’Donnell et al. 2011). *Morchella exuberans*, corresponding to *Mel*-9, has an intercontinental distribution range (Richard et al. 2015). These two species, as well as *Mel*-13 and *Mel*-21 (two undescribed phylogenetically distinct species) have been successfully domesticated and commercially developed in China. Only the cultivation of these morels is not carried out on a large scale.

### ***Morchella eximia*** Boud.

*Morchella eximia* corresponds to phylogenetic species *Mel*-7, although whether *M. eximia* and *M. septimelata* are conspecific has not yet been conclusively determined (Loizides et al. 2022). In China, *M. eximia* has a common name “Qimei Yangdujun”. Some patented methods have been shown to promote the production of primordium and improve its yield and quality (Zhao and Yang 2018, 2020). However, at present, the large-scale cultivation of *M. eximia* is still in the domestication and testing stage.

### ***Morchella importuna*** M. Kuo, O’Donnell & T.J. Volk

The black morel, *Morchella importuna,* is an intercontinental species, which is widely distributed in Asia, Europe and North America. It is a kind of facultative fire-adapted species that corresponding to phylogenetic species Mel-10 (O’Donnell et al. 2011; Kuo et al. 2012). In China, Zhao and Yang (2019a, 2019b) described the cultivation method of *M. importuna*. In their patents, they introduced in detail the strain production methods and the key technologies of field cultivation. In recent years, with the development and wide application of exogenous nutrient bags, the artificial cultivation of *M. importuna* was successful and the scale of cultivation was rapidly expanded in China.

### ***Morchella oweri*** X.H. Du [as '*owneri*']

*Morchella oweri* is morphologically similar to *M. sextelata*, *M. exuberans*, and *M. importuna* in distinctive capitate elements on the sterile ridges, darkening edges and a floccose stipe (Du et al. 2019b). However, it apparently does not have post-fire adaptability and is currently only distributed in northern China associated with *Pinus* at low altitudes. This species has also been domesticated and bred in China.

### ***Morchella sextelata*** M. Kuo

*Morchella sextelata*, an obligate fire-adapted species, corresponds to the phylogenetic species Mel-6 in O’Donnell et al. (2011). At present, some varieties of *M. sextelata* have recently been popularized in China, including “Kunzhi morel No.1” and “Kunzhi morel No.2” selected and bred by Kunming Institute of Botany (KIB), Chinese Academy of Sciences; “Guiyun No. 58” and “Guiyun No. 105” selected by Guizhou Institute of Technology and KIB; and “G” series of *Morchella sextelata* selected and bred by associate researcher Fang-He Tan of Sichuan Academy of Forestry Sciences (Zhao et al. 2021).

### Key techniques in the field cultivation of morels

At present, farmland and forest farming are the main morel cultivation methods. The cultivation protocol consists of spawn production, land preparation and spawning, an exogenous nutrition supply, fruiting management and harvesting (Liu et al. 2017, 2018; Zhao et al. 2021).

#### Spawn production

**Fig. 17:**
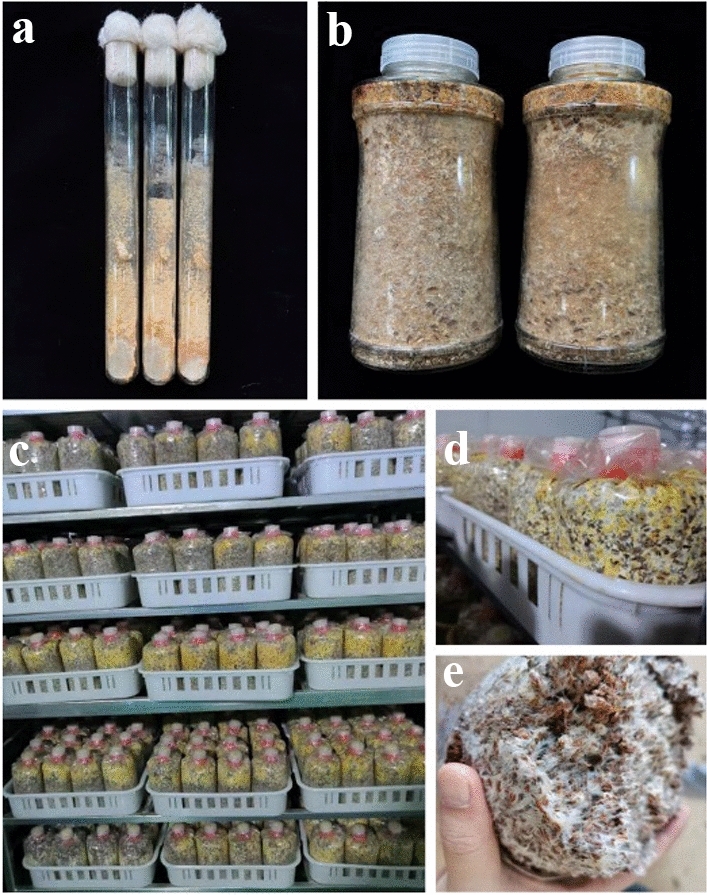
Morel spawn. **a** mother culture. **b** mother spawn. **c**, **d** final spawn with numerous sclerotia. **e** white stout mycelia of morels in the final spawn

High quality morel spawn is the most critical factor in successful cultivation. Morel spawns are divided into three types, i.e., mother cultures (Fig. 17a), mother spawn (Fig. 17b), and final spawn (Fig. 17c, d, and e).

#### Land preparation

Growing morels in the field is easily affected by external factors, such as the temperature and humidity of air and soil, light, water, the soil physicochemical property, and the soil microbial community. Normally, before cultivating, it is necessary to test the soil physicochemical properties and microflora. Besides, pesticides, herbicides and other chemical reagents also needs to be tested.

#### Spawning

**Fig. 18:**
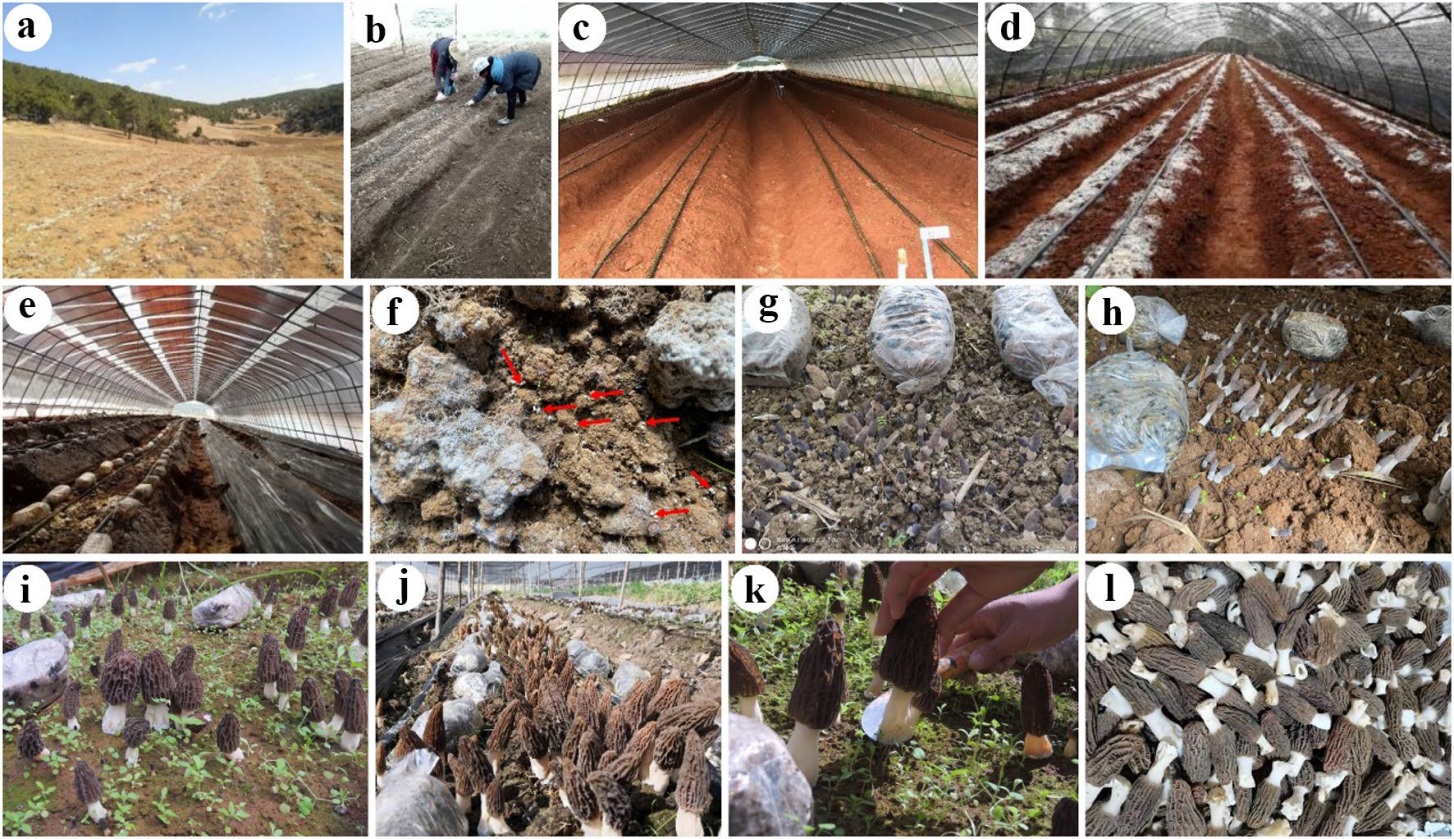
The morel cultivation protocol. **a** land preparation. **b** bedding and ditching. **c, d** spawning, casing, and watering. **e** exogenous nutrition aiding. **f, g** primordium. **h** nascent fruiting bodies. **i, j** mature fruiting bodies. **k** harvesting. **l** commercial morels

Spawning is usually carried out when the local maximum temperature is ≤ 20 °C, and the soil moisture should be maintained at 50–60%. The amount of final spawn is about 3000–4500 kg/ha (Fig. 18b). The soil is immediately covered after sowing, about 2–3 cm thick (Fig. 18c). The covered soil must be weed-free, stone-free, grainy, permeable, and retain moisture.

#### Exogenous nutrient bag addition

About 7–15 d after spawning, a vast expanse of “powdery mildew” appears on the surface of the mushroom bed, which is composed of mycelia and conidia of morels (Fig. 18d). At this time, exogenous nutrient bags can be added. The bags are 120 mm × 270 mm, net worth ≥ 300 g/bag, and the usage is 36,000–45,000 bags/ha. A mulch film is immediately added after placing exogenous nutrient bags (Fig. 18e).

#### Fruiting and harvesting

During the morel cultivation process, the humidity on the soil surface should be maintained at more than 50%. Primordia and fruiting bodies form in large quantities 55–70 days and 70–120 days after spawning. When morels are mature, the fruiting bodies grow to 7–12 cm with an obvious ridge and sinus, and the color deepens, the fruiting bodies can be harvested (Fig. 18i–l). Fruiting bodies can be sold fresh or dried at low temperature for later sale.

### Disease on cultivated morels

**Fig. 19:**
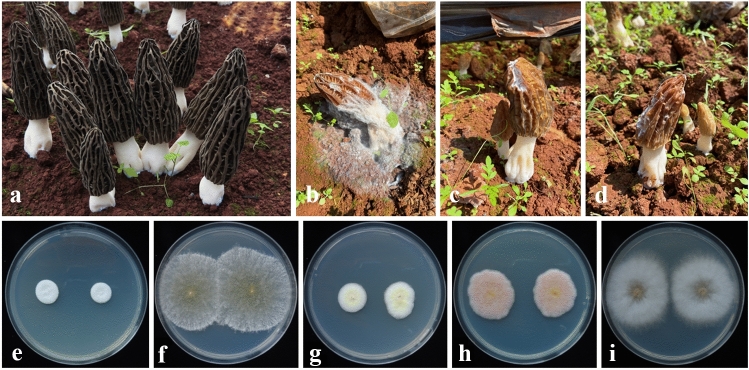
Symptoms of fungal disease on *Morchella sextelata* (**b-d**). Colony of fungal disease on PDA medium that isolated from infected morels (**e-i**). **a** Healthy fruiting bodies. **b** Diseased fruiting body infected *Cladobotryum mycophilum*. **c, d** Diseased fruiting body infected *Diploöspora longispora*. **e** *Diploöspora longispora*. **f** *Cladobotryum mycophilum*. **g** *Clonostachys rosea*. **h** *Fusarium* sp. **i** *Fusarium sambucinum*

With the expansion of cultivation range and density of morel production, disease have become the main factor limiting its yield. Pileus rot disease (He et al. 2018a), stipe rot disease (Guo et al. 2016), white mold disease (He et al. 2017; Wang et al. 2020; Chen et al. 2021), and cobweb disease (Lan et al. 2020) are currently considered to be the four most serious diseases in morel industry (Fig. 19). These fungal diseases occur to varying degrees in most cultivated areas at any time, threatening the production of morels and causes economic losses.

Pileus rot disease is caused by *Diploöspora longispora*, which leads to the malformed fruiting bodies (Fig. 19c, d). The infected morel tissues (mainly the pileus) are initially covered by white and velvety mycelia. Then, under the conditions of high temperature (≥ 25 ℃) and high relative humidity (≥ 90%), the disease spots quickly spread to the whole pileus and surrounding healthy fruit bodies, causing withering and decay (He et al. 2018a).

Cobweb disease is characterized by the rapid growth of cobweb-like mycelium over the affected mushrooms (Fletcher and Gaze 2007). This disease has become very common, and it is a serious cause of crop loss, causing great damage to various edible mushrooms including *Agaricus bisporus* (Back et al. 2010), *Ganoderma sichuanensis* (Zuo et al. 2016), *Hypsizygus marmoreus* (Back et al. 2012), *Morchella importuna* (Lan et al. 2020), *Pleurotus ostreatus* (Gea et al. 2019). Some species within *Cladobotryum* have historically been considered as the most common cause of cobweb disease (Fletcher and Gaze 2007). Lan et al. (2020) reported that *Cladobotryum protrusum* caused cobweb disease on cultivated *M. importuna*. The typical symptoms of this disease on morels are: white coarse mycelia appear on the soil surface and the base of stipe, which rapidly colonize and spread to the whole fruiting bodies. These symptoms can lead morels to wither and rot (Lan et al. 2020).

White mold disease is another major disease on morels that infected by *Paecilomyces penicillatus*. Once a morel farm is infected with white mold disease, 80% of morel production will be lost because *P. penicillatus* spreads uncontrollably rapidly (Wang et al. 2020). Dual culture assays showed that certain contact-independent soluble compounds secreted by *P. penicillatus* can inhibit the mycelial growth of *M. sextelata* (Wang et al. 2020). Genomics data demonstrated that *P. penicillatus* encodes a large number of fungal cell wall degradation enzymes (Wang et al. 2020), and transcriptome analysis showed that the genes involved in diphthamide biosynthesis, aldehyde reductase, and NAD (P)H-hydrate epimerase in *P. penicillatus* were up-regulated during the process of this fungus infection of *M. importuna* (Chen et al. 2021).

Stipe rot disease of *Morchella importuna* caused by *Fusarium incarnatum*–*F. equiseti* species complex (Guo et al. 2016). The symptoms mainly occur on the stipe of morels: at first, small, watery circular scars appear on the lower part of stipe; then the lesions develop into dark brown and sunken patches with sparse white hyphae on the surface; finally, under the condition of high temperature and humidity, these lesions expand rapidly, leading to rotting and shrinking of morel fruiting bodies (Guo et al. 2016). In addition, other species within *Fusarium*, such as *F. avenaceum*, *F. chlamydosporum*, *F*. *nematophilum*, *F. oxysporum* and *F. redolens*, have been reported to cause morel disease and affect yield and quality.

### Conclusion

In recent ten years, gratifying progress has been made in the artificial cultivation of morels. Although various aspects related to morel cultivation have been studied, including their reproductive and trophic modes (Du et al. 2017; Du and Yang 2021), interspecific hybridization and breeding (Du et al. 2016, 2019a; He et al. 2020), cultivation conditions (He et al. 2018b), and morel-associated microbial communities (Yu et al. 2022), there are still gaps between cultivation practices and basic knowledge of morel biology (Du and Yang 2021). In China, more than 70% of the growers cannot obtain stable profits in fact, and large-scale cultivation of morels (contiguous farms ≥ 3 ha) is still a high-risk project (Zhao et al. 2021). At present, the main morel cultivation methods are farmland farming and forest farming, morel yield and quality are great influenced by cultivation substrates (soil nutrients, microbial communities, and microbial metabolic components), and environment (light, temperature, water, humidity, and oxygen). In addition, the life histories and genetic characteristics of morels are still unclear, the cultivation mode is not standardized, and the management method is unscientific, which seriously limit the healthy and sustainable development of morel industry.

Facing the challenge of a "fast, scattered and chaotic" cultivation of morels in China, a sustained and steadily developing morel industry is called for, which must be strengthened by scientific and technological support, standard policy, standardized development and orderly promotion. It is particularly important for government functional departments to intervene in the control of the qualification certification of strains and the standardization of industry management. Moreover, scientists should focus on solving the bottlenecks encountered in the cultivation of morels, including: (1) designing an evaluation system for morel spawn quality; (2) breeding more cultivars; (3) systematically investigating the microbial diseases, and taking preventive and control measures and (4) improving, practicing and popularizing the industrialized cultivation of morels. Therefore, it is necessary for government departments to guide individuals, scientific research institutions and commercial organizations to jointly build a credible and authoritative third-party platform to supervise and guide the healthy development of morel industry.

## Fungal genera degrading synthetic plastic polymers

### The plastic wastes disposal problem

Global annual production of synthetic plastic polymers was 380 million tons in 2015 (Geyer et al. 2017). This has been divided into chemical types by previous publications (e.g., Danso et al. 2019). But they typically leave out some petroleum-based types (e.g., polycarbonate and acrylonitrile butadiene styrene). They also have excluded sustainable (bio)plastics derived from biomass (Zhu et al. 2016), and herein we add the amounts of those sources (Table 5), as available data allow.

**Table 5:** Global production rates of synthetic plastic polymers, and numbers of fungal genera capable of degrading them. Fungal genera shown to degrade each polymer are enumerated in Table 6

Polymer types	Million tons /yr	Fungal genera shown to degrade	Reference for polymer production rates
Bioplastics	2	54	https://www.european-bioplastics.org/global-market-for-bioplastics-to-grow-by-20-percent/#:~:text=The%20global%20bioplastics%20production%20capacity%20is%20set%20to,growth%20in%20the%20field%20of%20bio-based%2C%20biodegradable%20plastics
Polyethylene	116	21	Danso et al. (2019)
Polypropylene	68	6	Danso et al. (2019)
Polyvinyl chloride^1^	38	19	Danso et al. (2019)
Polyethylene terephthalate	33	6	Danso et al. (2019)
Polyurethane	27	34	Danso et al. (2019)
Polystyrene	14	12	Danso et al. (2019)
Acrylonitrile Butadiene Styrene^2^	12	0	https://www.statista.com/statistics/856670/acrylonitrile-butadiene-styrene-global-production-capacity/
Polycarbonate	8	6	Danso et al. (2019)
Polyaramids (nylon)^2^	8	2	Danso et al. (2019)
Polyvinyl acetate	7	8	http://www.polyvinylacetate.cn/pvac-news/2019_polyvinyl_acetate_market_report.html
Polyester	0.54	8	https://www.statista.com/statistics/912301/polyester-fiber-production-worldwide/
Ethylene vinyl acetate	< 1?	0	Proprietary^3^
Polyacrylic acid	< 1?	2	Proprietary^3^
Polyacrylamide^2^	< 1?	2	Proprietary^3^

Polymers containing chlorine^1^ or nitrogen^2^ are unsuitable for bioenergy production (see text). Production rates of some minor polymers^3^ are only available by purchasing market reports

### Global fates of discarded synthetic plastic polymers

According to Geyer et al. (2017), by 2020, 8.2 billion tons of plastics would have been generated, with 6.4 discarded as waste, 1.4 burned, and 1 recycled. It is notable that nitrogen- and chlorine-containing plastic polymers are unsuitable for bioenergy because they produce toxic combustion byproducts (Shen et al. 2016; Datta and Włoch 2017). For land-filled plastics, the absence of UV light (for photodecomposition) and oxygen (for biodegradation) results in persistence for centuries or longer (Glaser 2019). About 3% of plastic wastes escape to oceans (Jambeck et al. 2015).

Potential environmental damage from discarded synthetic plastic polymers have been examined for marine and terrestrial environments (e.g., Bergmann et al. 2015; Pawar et al. 2016; Iqbal et al. 2020; Rillig and Lehmann 2020; Rillig et al. 2021). While those are beyond our scope here, we stress that uncertainties remain large. Therefore, ways are being sought to minimize these amounts and risks.

### Potential solutions provided by fungi

Fungi are capable of decomposing essentially all carbon-containing polymers that nature or man has developed by means of extracellular enzymes (Tortella et al. 2005). The best natural example is decomposition of lignin (Floudas et al. 2012), a randomly ordered polymer second only to cellulose in global production, and far more than all plastic polymers combined. It seems reasonable to suppose that no plastic polymer can avoid fungal decomposition under appropriate conditions.

Many fungal genera have been shown to decompose many plastic polymers. From citations in literature reviews (Howard 2011; Kale et al. 2015; Ahmed et al. 2018; Wierckx et al. 2018; Paço et al. 2019; Raddadi and Fava 2019; Ghatge et al. 2020; Lee and Liew 2020; Magnin et al. 2020; Ru et al. 2020; Sánchez 2020; Inderthal et al. 2021; Kundungal et al. 2021; Taghavi et al. 2021) and further literature searches with Microsoft Academic, we developed Table 6. The number of fungal genera degrading each plastic polymer type are also shown in Table 5, along with annual production rates where those have been disclosed.

**Table 6:** Plastic polymer types demonstrated to be degradable by fungal genera of the phyla Ascomycota, Basidiomycota, and Mucoromycota

Polymer type	Phyla	Genera count	Genera	Data sources
Bioplastics—poly(butylene adipate-coterephthalate), poly(butylene succinate), poly(lactic acid), poly-3-hydroxyalkanoates, polycaprolactone	Ascomycota	45	*Acremonium, Acrostalagnus, Alternaria, Arthrinium, Aspergillus, Asteromyces, Aureobasidium, Beauveria, Bionectria, Botrytis, Camarosporium, Candida, Cephalosporium, Chaetomium, Cladosporium, Clonostachys, Colletotrichum, Curvularia, Debaryomyces, Diaporthe, Doratomyces, Emericellopsis, Fusarium, Gliomastix, Ilyonectria, Lanatonectria, Lophiostoma, Metapochonia, Metarhizium, Nectria, Neofusicoccum, Paecilomyces, Penicillium, Plectosphaerella, Pseudogymnoascus, Rhynchosporium, Sarcopodium, Sarocladium, Talaromyces, Thermoascus, Thermomyces, Trichothecium, Trichoderma Tritirachium, Verticillium*	Abe et al. (2010), Abou-Zeid et al. (2001), Darby and Kaplan (1968), Geweely and Ouf (2011), Gonda et al. (2000), Jarerat and Tokiwa (2001), Kasirajan and Ngouajio (2012), Kim et al. (2000), Lee et al. (2005), Maeda et al. (2005), Matavulj and Molitoris (1992), Matavuly and Molitoris (2009), Mergaert et al. (1993), Muhamad et al., (2015), Oda et al., (1995), Sanchez et al. (2000), Sowmya (2019), Szumigaj et al. (2008), Tokiwa et al. (2009), Torres et al. (1996), Weinberger et al. (2020)
	Basidiomycota	6	*Cryptococcus, Phanerochaete, Pleurotus, Polyporus, Pseudozyma, Rhodosporidium*	Abdel-Motaal et al. (2014), da Luz et al. (2013), Geweely and Ouf (2011), Gonda et al. (2000), Hidayat and Tachibana (2012), Matavulj and Molitoris (1992), Seo et al. (2007)
	Mucoromycota	3	*Mortierella, Mucor, Rhizopus*	Iwamoto and Tokiwa (1994), Matavulj and Molitoris (1992), Shah et al. (2008), Tokiwa et al. (2009), Weinberger et al. (2020)
	Total genera→	54		
Polyurethane (PU)*	Ascomycota	30	*Alternaria, Arthrographis, Aspergillus, Aureobasidium, Bionectria, Candida, Chaetomium, Cladosporium, Curvularia, Edenia, Emericella, Fusarium, Geomyces, Gliocladium, Guignardia, Lasiodiplodia, Leptosphaeria, Monascus, Nectria, Paecilomyces, Penicillium, Pestalotiopsis, Phaeosphaeria, Phoma, Plectosphaerella, Pleosporales, Thermomyces, Trichoderma, Xepiculopsis, Zopfiella*	Álvarez-Barragán et al. (2016), Barratt et al. (2003), Boubendir (1993), Brunner et al. (2018), Cangemi et al. (2006), Cooney (1969), Cosgrove et al. (2007), Cosgrove et al. (2010), Crabbe et al. (1994), Danso et al. (2019), Darby and Kaplan (1968), Edmonds and Cooney (1968), El-Morsy et al. (2017), Fernandes et al. (2016), Filip (1979), Gunawan et al. (2020), Howard (2002), Ibrahim et al. (2009), Ibrahim et al. (2011), Kanavel et al. (1966), Khan et al. (2017), Loredo-Treviño et al. (2011), Magnin et al. (2019), Mathur and Prasad (2012), Matsumiya et al. (2010), Oprea and Doroftei (2011), Oprea et al. (2016), Osman et al. (2018), Raghavendra et al. (2016), Russell et al. (2011), Shuttleworth and Seal (1986), Sowmya (2019)
	Basidiomycota	3	*Apiotrichum, Cryptococcus, Phanerochaete*	Gunawan et al. (2020), Sharari et al. (2013), Zicht (2017)
	Mucoromycota	1	*Mortierella*	Cosgrove et al. (2010), Gunawan et al. (2020)
	Total genera→	34		
Polyethylene (PE)	Ascomycota	16	*Acremonium, Alternaria, Aspergillus, Chaetomium, Cladosporium, Curvularia, Fusarium, Gliocladium, Lasiodiplodia, Paecilomyces, Penicillium, Phialophora, Phoma, Trichoderma, Verticillium, Zalerion*	Abraham et al. (2017), Ameen et al. (2015), Balasubramanian et al. (2014), Bonhomme et al. (2003), Das and Kumar (2014), Deepika and Jaya (2015), Esmaeili et al. (2013), Gajendiran et al. (2016), Grover et al. (2015), Karlsson et al. (1988), Kathiresan (2003), Koutny et al. (2006), Manzur et al. (2004), Muhonja et al. (2018), Munir et al. (2018), Nowak et al. (2011a), Ojha et al. (2017), Paço et al. (2017), Raghavan et al. (1990), Raghavendra et al. (2016), Restrepo-Flórez et al. (2014), Sakhalkar and Mishra (2013), Sangale et al. (2019), Shah et al., (2009), Sindujaa et al. (2011), Singh and Gupta (2014), Singh et al. (2012a, b), Sowmya et al. (2015), Sowmya, (2019), Volke-Sepúlveda et al. (2002), Wadood et al. (2018), Yamada-Onodera et al. (2001), Zahra et al. (2010), Zhang et al. (2020a, b, c, d)
	Basidiomycota	2	*Phanerochaete, Trametes*	Iiyoshi et al. (1998), Orhan and Buyukgungor (2000), Shah et al. (2008), Sowmya et al. (2015)
	Mucoromycota	3	*Cunninghamella, Mortierella, Mucor*	Nowak et al. (2011a), Restrepo-Flórez et al. (2014), Sakhalkar and Mishra (2013), Singh and Gupta (2014)
	Total genera→	21		
Polyvinyl chloride (PVC)	Ascomycota	14	*Alternaria, Aspergillus, Aureobasidium, Chaetomium, Cladosporium, Cochliobolus, Emericella, Fusarium, Kluyveromyces, Paecilomyces, Penicillium, Phaeococcomyces, Phoma, Taphrina*	Ali et al. (2014), Gumargalieva et al. (1999), Moriyama et al. (1993), Sabev et al. (2006), Sakhalkar and Mishra (2013), Sumathi et al. (2016), Vivi et al. (2019), Webb et al. (1999), Webb et al. (2000)
	Basidiomycota	4	*Lentinus, Phanerochaete, Pleurotus, Rhodotorula*	Ali et al. (2014), Khatoon et al. (2019), Klrbas et al. (1999), Webb et al. (2000)
	Mucoromycota	1	*Mucor*	Sakhalkar and Mishra (2013)
	Total genera→	19		
Polystyrene (PS)	Ascomycota	6	*Aspergillus, Engyodontium, Exophiala, Gliocladium, Penicillium, Sporothrix*	Atiq (2011), Cox (1995), Cox et al. (1996), de Jong et al. (1990), Jeyakumar et al. (2013), Paca et al. (2001), René et al. (2010), Tian et al. (2017)
	Basidiomycota	5	*Bjerkandera, Gloeophyllum, Phanerochaete, Pleurotus, Trametes*	Atiq (2011), Braun-Lüllemann et al. (1997), Danso et al. (2019), Jeyakumar et al. (2013), Krueger et al. (2015, 2017)
	Mucoromycota	1	*Rhizopus*	Atiq (2011)
	Total genera→	12		
Polyester	Ascomycota	0	*–-*	
	Basidiomycota	8	*Agaricus, Inonotus, Irpex, Phanerochaete, Pleurotus, Polyporus, Pseudozyma, Stropharia*	Sasek et al. (2006), Shah et al. (2008), Shinozaki et al. (2013)
	Mucoromycota	0	*–-*	
	Total genera→	8		
Polyvinyl acetate (PVA)	Ascomycota	4	*Aspergillus, Fimetariella, Fusarium, Galactomyces*	Sowmya (2019)
	Basidiomycota	4	*Flammulina, Phanerochaete, Pycnoporus, Trichosporon*	Larking et al. (1999), Sowmya (2019), Tsujiyama et al. (2011)
	Mucoromycota	0	*–-*	
	Total genera→	8		
Polypropylene (PP)	Ascomycota	3	*Aspergillus, Engyodontium, Lasodiplodia*	Jeyakumar et al. (2013), Pandey and Singh (2001), Sheik et al. (2015)
	Basidiomycota	2	*Bjerkandera, Phanerochaete*	Butnaru et al. (2016), Jeyakumar et al. (2013)
	Mucoromycota	1	*Rhizopus*	Iwamoto and Tokiwa (1994)
	Total genera→	6		
Polycarbonate	Ascomycota	5	*Chrysosporium, Engyodontium, Fusarium, Penicillium, Ulocladium*	Arefian et al. (2013), Artham and Doble (2010)
	Basidiomycota	1	*Phanerochaete*	Artham and Doble (2010)
	Mucoromycota	0	–-	
	Total genera→	6		
Polyethylene terephthalate (PET)	Ascomycota	6	*Candida, Fusarium, Humicola, Penicillium, Thermomyces, Yarrowia*	da Costa et al. (2020), Danso et al. (2019), Liebminger et al. (2007), Nimchua et al. (2007), Nowak et al. (2011b), Ronkvist et al. (2009), Sepperumal et al. (2013)
	Basidiomycota	0	*–-*	
	Mucoromycota	0	*–-*	
	Total genera→	6		
Nylon (polyaramids)*	Ascomycota	1	*Fusarium*	Tachibana et al. (2010)
	Basidiomycota	1	*Trametes*	Chonde et al. (2012)
	Mucoromycota	0	*–*	
	Total genera→	2		
Polyacrylamide*	Ascomycota	0	*–*	
	Basidiomycota	2	*Phanerochaete, Pleurotus*	Mai et al. (2004)
	Mucoromycota	0	*–*	
	Total genera→	2		
Polyacrylic acid	Ascomycota	0	*–*	
	Basidiomycota	2	*Phanerochaete, Pleurotus*	Mai et al. (2004), Sutherland et al. (1997)
	Mucoromycota	0	*–*	
	Total genera→	2		

Presented in decreasing order of total numbers of fungal genera degrading each type, with totals shaded yellow. Polymers containing nitrogen are labeled with *. Not included here are acrylonitrile butadiene styrene* (ABS) and ethylene vinyl acetate (EVA) because no fungi have been identified that degrade those polymers

### Competence of fungi to degrade plastic polymers

Fifty-five fungal genera have been shown to degrade bioplastics, more than for any petroleum-derived polymers (Table 5). Demonstrated ranges of fungi degrading polypropylene, polyethylene terephthalate, acrylonitrile butadiene styrene, and polyaramids seem low compared to their production rates (Table 5). We suppose this is more likely due to incomplete examination, than from non-degradable polymer chemical structures.

Genera of Ascomycota or Basidiomycota have been shown to be capable of degrading all petroleum-based plastic polymers except for acrylonitrile butadiene styrene (ABS) and ethylene vinyl acetate (EVA) (Table 6). ABS and EVA exceptions are more likely due to lack of examination, than from non-degradable chemical structures. Genera of Ascomycota or Basidiomycota are capable of degrading all biomass-based plastic (bio)polymers. Perhaps surprisingly, more genera of Ascomycota (75) than Basidiomycota (19) can degrade plastic polymers. In Ascomycota, *Aspergillus, Fusarium* and *Penicillium* degrade seven plastic types, with other genera having narrower ranges. In Basidiomycota, *Phanerochaete* can degrade ten plastic types, *Pleurotus* six, with other genera having narrower ranges. This capability of *Pleurotus*, along with other genera containing edible mushrooms is interesting, as it raises the possibility that bioremediation of plastics might be combined with production of edible sporocarps. This does not seem to have been researched. In Mucoromycota, four genera can degrade two or three types of plastic polymers (Table 6).

It is also important to note that cited studies examined polymers individually. Solving the global plastic-polymer waste problem requires developing systems (based on single microbes or consortia) that can degrade polymer mixtures, which will also include many other types of discarded materials. No study is apparent that explores fungal biodegradation of mixed wastes arriving at landfills. Such biodegradation research has been aimed at publishable results, not solutions for real-world problems. Degradation rates of pure polymers are reported in some studies, but by varying techniques that preclude comparisons. Most importantly, few attempts are made to optimize degradation rates, and mixed sources have never been examined.

### Products of fungal biodegradation of plastic polymers

Reverting plastic polymers to CO_2_ is the (generally unstated) goal, but uncaptured CO_2_ is not valuable. Reverting these to useable hydrocarbon monomers would have value. Such research has been carried out with fungi, bacteria, and their derived enzymes acting on (some pure) polymer source streams and is outside our scope here. We can say at least that fungi show great potential to reduce accumulations of all types of plastic debris, and reduce harm that may result from them. Research into fungal degradation of plastic polymers began decades ago, but 54% of references cited in Table 6 are from 2010 or are more recent.

Another interesting aspect is that some insects consume and degrade plastic polymers (Ali et al. 2021), with a few demonstrated to do so by way of gut fungi (Khan et al. 2021). Most insects that have been examined were chosen because they are used as feed sources for fish and chickens. This could be ‘waste upcycling’ if it were commercialized (Khan et al. 2021).

### Enzymes involved in degradation of plastic polymers

Fungi degrade plastic polymers with extracellular enzymes, and the breadth of such processes has been recently reviewed by Srikanth et al. (2022), Temporiti et al. (2022) and Devi et al. (2016). The summarized findings from those reviews are given in Table 7. Fungal extracellular enzyme roles in plastic polymer degradation remain poorly known, both in terms of ranges of capable fungi and their plastic polymer targets. Fungi can play a large role in addressing the plastics-waste problem, but substantial knowledge gaps remain.

**Table 7:** Enzyme classes and types known to degrade plastic polymers

Polymer type	Enzyme class	Enzyme type	Data sources
Bioplastics—poly(butylene succinate (PBS)	Hydrolytic	Lipases (EC 3.1.1.3)	Srikanth et al. (2022)
Bioplastics—poly(butylene succinate (PBS)	Hydrolytic	Cutinases (EC 3.1.1.74)	Devi et al. (2016)
Bioplastics -polybutylene succinate (PBS), polybutylene succinate-co-adipate (PBSA)	Hydrolytic	Lipases (EC 3.1.1.3)	Srikanth et al. (2022)
Bioplastics -polycaprolactone (PCL)	Hydrolytic	Lipases (EC 3.1.1.3)	Srikanth et al. (2022), Devi et al. (2016)
Bioplastics -polycaprolactone (PCL)	Hydrolytic	Cutinases (EC 3.1.1.74)	Devi et al. (2016)
Bioplastics -polylactic acid (PLA)	Hydrolytic	Esterases (EC 3.1.1.x)	Srikanth et al. (2022)
Polyurethane (PUR)	Hydrolytic	Esterases (EC 3.1.1.x)	Temporiti et al. (2022), Srikanth et al. (2022)
	Hydrolytic	Lipases (EC 3.1.1.3)	Temporiti et al. (2022), Srikanth et al. (2022)
	Hydrolytic	Cutinases (EC 3.1.1.74)	Temporiti et al. (2022)
	Hydrolytic	Urease (EC 3.5.1.5)	Devi et al. (2016)
	Hydrolytic	Serine hydrolase (EC 3.4.16)	Devi et al. (2016)
Polypethylene (PE)	Oxidoreductase	Laccase (EC 1.10.3.2)	Srikanth et al. (2022), Devi et al. (2016)
	Oxidoreductase	Manganese peroxidase (EC 1.11.1.13)	Srikanth et al. (2022), Devi et al. (2016)
	Oxidoreductase	Lignin peroxidase (EC 1.11.1.14)	Srikanth et al. (2022)
Polyvinyl chloride (PVC)	Oxidoreductase	Peroxidase (EC 1.11.1.14)	Temporiti et al. (2022)
	Oxidoreductase	Laccase (EC 1.10.3.2)	Temporiti et al. (2022), Srikanth et al. (2022)
Polystyrene (PS)	Hydrolytic	Esterases (EC 3.1.1.x)	Temporiti et al. (2022)
Polyethylene terephthalate (PET)	Hydrolytic	Cutinases (EC 3.1.1.74)	Temporiti et al. (2022)
	Hydrolytic	Lipases (EC 3.1.1.3)	Temporiti et al. (2022)
	Hydrolytic	Carboxylesterases (EC 3.1.1.1)	Temporiti et al. (2022)

Follows polymer types as in Table 6 and excludes those without enzyme information. Enzyme types include standard EC nomenclature

### Conclusion

Fungi can probably degrade all plastic polymers, however sourced, with CO_2_ as the end product (not always stated). Green (bio)plastics may be not dramatically more susceptible to fungal biodegradation than petroleum-based plastics, under optimized conditions. Precise conclusions are not possible because biodegradation studies have used varied methods. The use of single taxa or consortia in waste disposal treatments however has not been well-researched. This is an important area for future studies.

## Discussion

We have written about what we consider to be the ten most important decadal advances in fungal biology leading towards human well-being, but there are many more significant discoveries, and we discuss a few below.

## Fungal diversity

Over the past ten years there has been a colossal advance in the classification and description of novel species of fungi. This has mainly been due to the use of molecular tools, but also more research efforts being carried out in prosperous, previously developing nations, such as Brazil, China and Thailand (Hyde et al. 2018b, 2021; Boonmee et al. 2021; He and Zhao 2022). Publication outlets such as Fungal Diversity Notes (Hyde et al. 2020a; Yuan et al. 2020; Boonmee et al. 2021), Fungal Planet (Crous et al. 2020a,b, 2021a; b) and Mycosphere notes (Pem et al. 2019; Hyde et al. 2021) have introduced more than 2000 novel taxa. The change from dual nomenclature to a single name for holomorphic genera and species has been pivotal. This has resulted from the realization that molecular data can link taxa of different sexually (Karunarathna et al. 2017; Wanasinghe et al. 2017; Jayasiri et al. 2019; Phookamsak et al. 2019; Devadatha et al. 2020; Maharachchikumbura et al. 2021; Senanayake et al. 2022). It has also been realized that many plant pathogenic genera contain numerous species complexes with each comprising numerous taxa which may infect different hosts (Bhunjun et al. 2020, 2021; Jayawardena et al. 2021a). The work towards the classification of the fungi (Maharachchikumbura et al. 2015; Thambugala et al. 2015; Tian et al. 2016; Daranagama et al. 2018; Senwanna et al. 2019; Dong et al. 2021) culminated in the first outline of the Fungi and fungus-like organisms (Wijayawardene et al. 2020, 2022) and more detailed classifications of various classes, including basal fungi (Hurdeal et al. 2021), basidiomycetes (He et al. 2022), Dothideomycetes (Hongsanan et al. 2020), Sordariomycetes (Hongsanan et al. 2017; Hyde et al. 2020c) amongst others (Ekanayaka et al. 2019; Johnston et al. 2019). Jeewon and Hyde (2016) provideded guidleines for describing a new species and the need for polyphyletic approaches was emphasised in the special issue, *What is a species?* (Boekhout et al. 2021; Cao et al. 2021a; Chethana et al. 2021b; Jayawardena et al. 2021b; Lücking et al. 2021; Maharachchikumbura et al. 2021; Manawasinghe et al. 2021; Pem et al. 2021; Voigt et al. 2021). The important repositories for taxa were developed earlier but in the last decades numerous new websites have been developed including sites on insect (Wei et al. 2022, https://invertebratefungi.org/references.php), freshwater (Calabon et al. 2020, https://freshwaterfungi.org/) and marine fungi (https://www.marinefungi.org/). There are also sites on fungi of the United Kingdom, the Greater Mekong Subregion (Chaiwan et al. 2021, https://www.gmsmicrofungi.org/) and Italian ascomycetes (Wijesinghe et al. 2021, https://italianmicrofungi.org/) and sites on the Genera of fungi (Monkai et al. 2019, https://fungalgenera.org/) and Fungalpedia (https://fungalpedia.org/).

## Whole genome sequencing

Whole-genome sequencing has been carried out for numerous species in the past decade. Advances made from these data mainly include phylogenomics for taxonomic organization, designing effective therapies against targeted fungal pathogens, and the development of new antibiotics, pharmaceuticals and secondary metabolites necessary for industrial applications, among many others. The use of whole-genomes in phylogenomic studies provides sufficient data to elucidate relationships deeper in geological time, as well as to resolve relationships that evolved in short divergence times, which may lead to resolve problems in taxonomy (James et al. 2020). Further studies on these whole-genome sequences allow to perform functional genomic studies on the genes predicted from the whole genome sequences, thus providing new knowledge to predict their lifestyles (Gómez-Pérez and Kemen 2021). The availability of genomic data also enables to assess micro- and macro variations within a species in a population to determine their genomic evolution. Furthermore, mining whole-genome sequences allows the identification of proteins responsible for host interactions and secondary metabolites for various applications. The knowledge produced from gene functions and their metabolic pathways is important for designing therapies as an alternative to drugs (Guo and Wang 2014). Identification of genes involved in host interactions, specifically in plant pathogenic fungi, produces new knowledge required to predict the emergence of fungal diseases and the surveillance of plant health. This is not only limited to phytopathogenic fungi but also applies to fungal diseases in humans, where they detect and monitor the spread of the disease, determine the distribution of the pathogen, predict outbreaks, and their evolution during outbreaks (Cuomo 2017). Furthermore, information on endophytic fungal genomes facilitates the development of alternatives for pesticides and fertilizers. In addition, advances in whole-genome studies facilitate the genotyping of pathogenic species for diagnostic purposes. These diagnostics have been applied to both human and plant pathogens for their precise and rapid detection and identification, which is crucial to managing the diseases effectively (Kidd et al. 2020; Hariharan and Prasannath 2021).

## Biological control of pests

Biological control can be defined as the inhibition of growth, infection or reproduction of one organism using another organism (Cook 1993). This can involve the use of microbial inoculants to suppress a single type of plant disease as well as managing soil to promote the soil and plant-associated organism that can contribute to the general suppression of disease (Cook 1993). This method is environmentally friendly and sometimes the only option available (Barratt et al. 2018; Hyde et al. 2019). A comprehensive understanding of the complex interactions among plants and the environment is needed when implementing biological control. Biocontrol of the unwanted organisms can achieve through antibiosis, competition, metabolite production, and mycoparasitism (Xu et al. 2011).

During the past decade, much research has been conducted to identify potential fungal species that can be used as bio-control agents against plant diseases (Thambugala et al. 2020). *Acremonium alternatum*, *Acrodontium crateriforme*, *Ampelomyces quisqualis*, *Cladosporium oxysporum* and *Trichoderma virens* can hyperparasitize the powdery mildew pathogens (Milgroom and Cortesi 2004). Fungal epiphytes of banana namely *Clonostachys byssicola*, *Curvularia pallescens*, *Penicillium oxalicum* and *Trichoderma harzianum* showed antagonistic activity against the banana crown-rot causing pathogens *Thielaviopsis paradoxa*, *Colletotrichum musae*, and *Fusarium verticillioides* significantly affected the mycelial growth and conidial germination of the pathogens (Alvindia and Natsuaki 2008). Endophytic fungi have been shown to have an antagonistic activity towards pathogenic fungi as well as influence the host resistance (Hyde et al. 2019).

So far, species of *Trichoderma* have proven to be the most effective biocontrol agents (Alvindia and Acda 2012). *Trichoderma* species are filamentous fungi, found in a variety of ecosystems (Jayawardena et al. 2019a, b) and use mycoparasitism to attack the host and with various enzymes degrading the target cell (Benítez et al. 2004; Sood et al. 2020). Secondary metabolites produced by this group of fungi have antibiotic properties (Vinale et al. 2014) which helps the plants to fight against diseases. *Trichoderma* species can be used as nematicidal agents (*T. asperellum*, *T. brevicompactum*, *T. citrinoviride*, *T. harzianum* and *T. viride*), insecticides (*T. longibrachiatum*) and as fungicides (*T. asperellum*, *T. viride*, *T. harzianum*, *T. koningii, T. longibrachiatum*) (Ferreira and Musumeci 2021; Poveda 2021).

Recently there has been a renewed interest in fungal pathogens of insects due to their potential as biocontrol agents. More than 750 species of fungi have been identified to be pathogenic to insects offering a great potential for pest management (Sharma and Sharma 2021; Poveda 2021). *Beauveria bassiana*, *B. brongnia*rtii, *Cladosporium oxysporium*, *Metarhizium anisopliae*, *Hirsutella thompsonii*, *Isaria fumosorosea* and *Lecanicillium* spp., are among the species that are already used in formulated mycoinsecticides (Maina et al. 2018). Another successful application of fungi can be seen in the application of *Beauveria bassiana* for the control of pine moths (*Dendrolimus* spp.) in China (Kovač et al. 2020). *Beauveria bassiana* (strain Bb-147) is used as a registered product in Europe to control the European corn borer (*Ostrinia nubilalis*) and the Asiatic corn borer (*O. furnacalis*) (Batool et al. 2020).

## Beneficial use of a toxin: gliotoxin

Another interesting decadal advance lies in gliotoxin, although this may never be taken up by pharmaceutical companies as they already have a swathe of drugs to treat acquired immunodeficiency syndrome (AIDS). However, it does show the potential of fungi.

AIDS is a well-known sexually or blood-transmitted viral disease which, despite improved antiviral medication, still causes half a million deaths each year. Upon infection, the human immunodeficiency virus (HIV) enters and kills the T-helper (CD4^+^) immune cell in the process of replicating. With this impaired adaptive immunity, the patient becomes susceptible to a plethora of pathogenic and opportunistic microorganisms. Current and very successful treatment is a combination of inhibitors of HIV integrase, reverse transcriptase and proteases known as c-ART (combination AntiRetroviral Therapy). This has effectively reduced the burden of disease in Western countries, to such an extent that patients can live with the infection for decades, and in this part of the world AIDS is no longer seen as a threat, but rather HIV infection has become a chronic disease. However, the death toll outside the industrialized world where access to medication is limited is still unacceptably high. In addition, the disease is suppressed rather than eradicated, because the virus remains dormant in a small reservoir of infected cells, a process known as latency. Much research has therefore been devoted to get the virus out of the cells, using latency reversal agents (LRAs). The virus then becomes in reach for elimination by the immune system and presence of c-ART therapy prevents new rounds of infection. Numerous compounds have been proposed, but most of these were either toxic, or insufficiently effective and have failed thus far to make a significant impact on the latent HIV reservoir or lead to cure (reviewed in Stoszko et al. 2019).

**Fig. 20:**
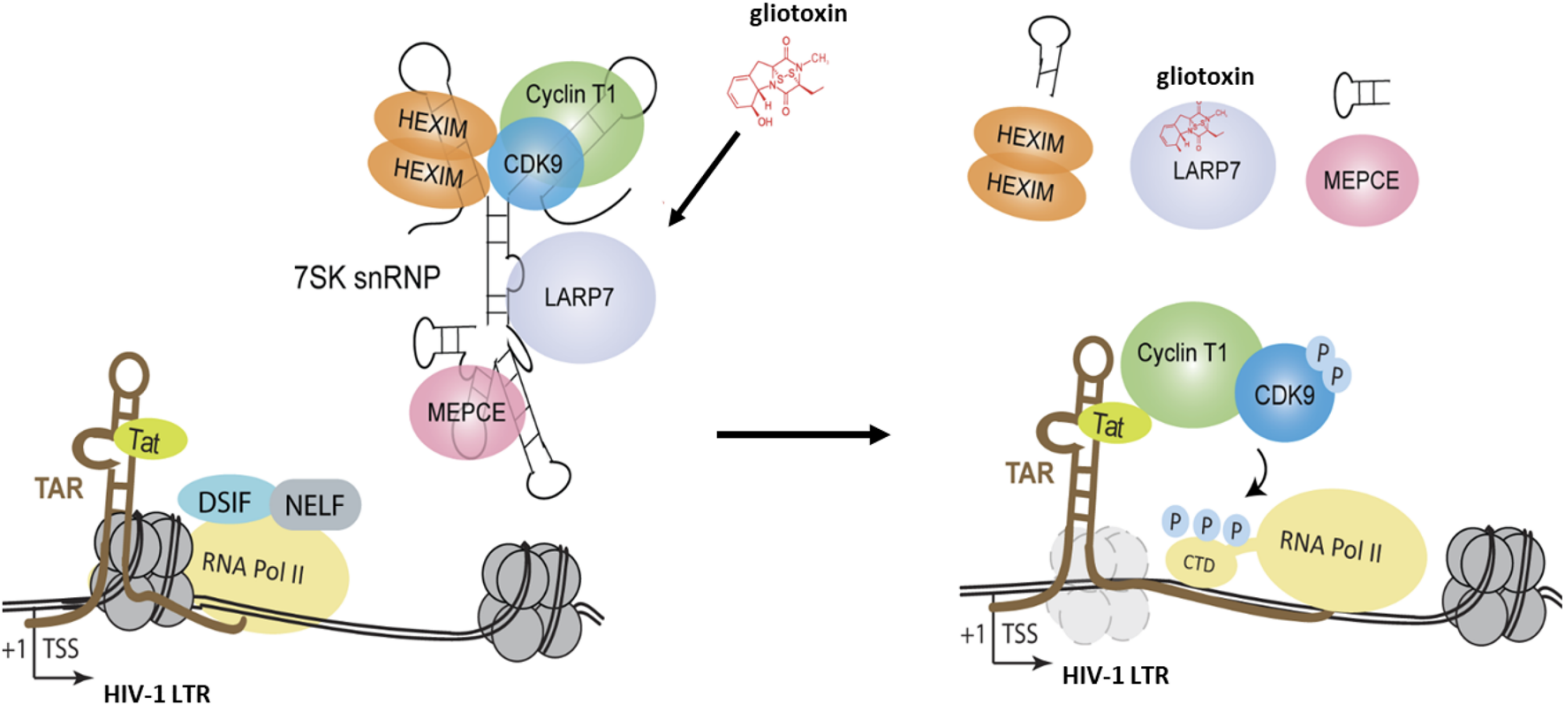
Proposed model of gliotoxin disruption of 7SK snRNP, causing release of P-TEFb and activation of the latent HIV-1 LTR via release of CDK9 from the 7SK snRNP complex. Free P-TEFb is then recruited to the HIV-1 Tat-TAR axis, leading to phosphorylation of RNA Pol II and subsequent stimulation of transcription elongation. *CDK9* cyclin-dependent kinase 9; *DSIF* 5,6-dichloro-1-beta-d-ribofuranosylbenzimidazole sensitivity inducing factor, *CTB* non-toxic B-subunit of cholera toxin, *HEXIM* hexamethylene bis-acetamide inducible 1; *LARP* La ribonucleoprotein domain family, *LTR* latency reversal, *MEPCE* methylphosphate capping enzyme, *NELF* negative elongation factor, *Pol* polymerase, *snRNP* small nuclear ribonucleic protein, *TAR* trans-activation response, *Tat* trans-activation of transcription, *TEF* transcription elongation factor; Modified after Stoszko et al. (2020)

Fungi produce a wide diversity of bioactive compounds that is largely unexplored. With this in in mind, Stoszko et al. (2020) conducted a study in search of novel LRAs. The authors screened a large diversity of fungi: 115 species belonging to 28 orders (43 families) dispersed over the fungal kingdom were included. Low and medium throughput screening systems of crude extracts of supernatants were dissected by orthogonal fractionation and mass spectrometry (MS) coupled to nuclear magnetic resonance (NMR). Extracts were stepwise tested in HIV latency reversal bioassays. Out of tens of thousands of compounds, finally gliotoxin (GTX) was identified as a novel LRA. Gliotoxin is a fungal extralite which is produced by *Aspergillus fumigatus* and some other species. The mechanism of action of GTX in reversal of latency in HIV-infected CD4^+^ T-cells is by disrupting 7SK snRNP, a complex that sequesters the positive transcription elongation complex (PTEFb), which is required for efficient HIV gene expression. When released from 7SK snRNP, PTEFb is then recruited to the HIV promoter by the viral Tat protein and phosphorylates RNA Pol II CTD, leading to increased HIV transcription (Fig. 20). Stoszko et al. (2020) employed several biochemical assays and transcriptome analyses to unravel the steps targeted by GTX to reverse latency. Also, the efficacy of synergistic combinations of GTX with other known LRAs was analyzed, and synergistic effects of caffeic phenethyl ester (CAPE), pyrimethamine (PYR) and macrolactams with gliotoxin were assessed. Activity and toxicity of GTX was further determined using model systems of HIV-1 latency using cells obtained from HIV-1 positive patients under c-ART therapy, and it was found that GTX latency reversal is reached at very low, non-toxic concentrations of GTX. Potential pleiotropic effects on other immune cells, such as CD8^+^ T-cells responsible for eliminating the infected CD4^+^ T-cells, remained absent.

Gliotoxin is a secondary metabolite of the diketopiperazines class. It is a well-known mycotoxin produced by species of *Aspergillus, Penicillium, Fusarium* and *Trichoderma,* fungi which are eutrophic and reside in nutrient-rich habitats such as composting debris. In these microbe-rich environments they have to compete for survival against a large diversity of fungi and bacteria by rapid growth and production of toxic metabolites Toxic effects of GTX against fungi (Carberry et al. 2012) and bacteria (Esteban et al. 2021) are due to redox-cycling of a disulphide-bridge. Among the toxic effects are expression of proteins, disturbance of enzymes, and leakage of mitochondrial membranes. In humans, it has immunomodulatory functions by interference with neutrophils and macrophages and impairs T-cell activation. This may contribute to the fact that several of the above saprobes also frequently occur as opportunistic pathogens (de Hoog et al. 2020). The action of GTX as LRA is achieved at concentrations far below the level of toxicity. Stoszko et al. (2020) convincingly showed that GTX provides a promising novel treatment option, which for in a pharmacological combination for HIV therapy, may move towards a cure rather than just suppression of the disease.
